# Temporal trends in late-pregnancy exposure to ambient temperature and risk of preterm birth in Japan, a nationwide study from 1979 to 2023

**DOI:** 10.1016/j.lanwpc.2026.101894

**Published:** 2026-06-04

**Authors:** Alton Quan Cao, Lei Yuan, Sophearen Ith, Yui Tomo, Chris Fook Sheng Ng, Daisuke Yoneoka, Masahiro Hashizume

**Affiliations:** a Department of Global Health Policy, Graduate School of Medicine, The University of Tokyo, Tokyo, Japan; b Department of Epidemiology, National Institute of Infectious Diseases, Japan Institute for Health Security, Tokyo, Japan; c Department of Global Environmental Health, Graduate School of Medicine, The University of Tokyo, Tokyo, Japan; d Department of Global Health, School of Tropical Medicine and Global Health, Nagasaki University, Nagasaki, Japan

**Keywords:** Preterm birth, Reproductive health, Non-optimal temperature, Prenatal exposure, Maternal exposure

## Abstract

**Background:**

Evidence linking heat exposure with preterm birth (PTB) has grown amid climate change. However, evidence remains limited across the temperature spectrum or temporal changes of susceptibility.

**Methods:**

To assess the temporal and spatial variation in the short-term association between ambient temperature and PTB in Japan from 1979 to 2023, we applied a two-stage design using a time-stratified case-crossover and conditional Poisson model incorporating a 28-day distributed lag non-linear model. Prefecture-specific estimates were pooled using random-effects meta-analysis. To explore temporal changes in susceptibility, the data were split into four decadal subperiods, and mixed-effects meta-regression examined prefecture-level predictors of changing risk.

**Findings:**

Between 1979 and 2023, 2,604,733 singleton PTBs (4.9% of 52,749,360 live births) occurred. Compared to the minimum morbidity temperature, 17.7 °C (56th percentile), relative risks (RRs) were 1.13 [95% CI: 1.10–1.15] for heat (99th percentile) and 1.11 [95% CI: 1.09–1.14] for cold (1st percentile). Nationally, 4.7% [4.3–5.0%] of PTBs (102,266 cases) were attributable to non-optimal temperatures (1.9% heat; 2.8% cold). Heat-related RR declined from 1.16 [1.12–1.21] in 1979–1989 to 1.07 [1.03–1.12; *p*: 0.0084] in 2001–2011 and then plateaued, while cold-related risk remained stable. Air conditioning prevalence was positively associated with MMT across periods.

**Interpretation:**

Over four decades, Japan experienced modest early declines in heat-related risk, with no further improvement in recent decades, alongside persistent cold-related PTB risk, suggesting limited heat adaptation. Pregnant women remain underrepresented in climate adaptation planning, underscoring the need for gender-inclusive resilience strategies.

**Funding:**

Japan’s Environmental Restoration and Conservation Agency.

**Research in context**

**Evidence before this study**

Rising temperatures pose increasing risks to maternal and neonatal health. Understanding how the risk of preterm birth (PTB) associated with ambient temperature has evolved over time is essential for anticipating the health impacts of climate change and guiding adaptation policy. In Japan, maintaining high-quality health services is increasingly challenging within a national system strained by aging and thinning rural communities, economic stagnation, and widening inequality. These pressures, coupled with the improbability of improvements given the advanced and ubiquitous nature of Japan’s maternal and child health services may exacerbate vulnerability to temperature extremes. Although evidence linking temperature to PTB has expanded rapidly, most studies have focused only on heat, and few have assessed the full temperature spectrum, or how susceptibility has changed over time. We searched English language publications in PubMed up to October 2025 using the terms (“temporal” OR “temporal trends” OR “longitudinal” OR “subperiod”) AND “temperature” AND (“preterm” OR “birth”). From this search, only one single-city study conducted in Australia (1994–2013) examined temporal variation in the ambient temperature-related PTB risk. To the best of our knowledge, no nationally representative studies have assessed how temporal and spatial heterogeneity shape temperature-related PTB risk.

**Added value of this study**

This study provides the first comprehensive, nationwide assessment of temperature-related PTB risk across more than four decades in Japan, using birth data from national vital statistics. By examining regional climatic, demographic, and socioeconomic factors, we also identified contextual drivers of temporal and spatial variation in susceptibility. The findings provide evidence of heat adaptation; however, both heat- and cold-related PTB risks and attributable burdens persisted in comparison to the extensive declines in temperature-related mortality observed globally across high-income settings. These findings highlight the enduring sensitivity of birth outcomes to temperature and the challenges of achieving reproductive health resilience in a changing climate.

**Implications of all the available evidence**

Despite advances in heat adaptation in this high-income context, we still found that expecting mothers remain insufficiently protected from the heat impacts of climate change. The persistent burden of both heat- and cold-related PTB risk underscores the need to integrate maternal and child health explicitly within climate adaptation agendas. Centering maternal and reproductive health within national climate and development legal frameworks will be essential to achieving the Sustainable Development Goals on health, gender equality and climate action, and to ensure equitable climate resilience.

## Introduction

Preterm birth (PTB), defined as live births at fewer than 37 weeks of gestation, is a critical health indicator, considered the leading cause of under-five mortality globally,^1^ and associated with lifelong health disorders.^2^^,^^3^ Every year, 13.4 million babies are born preterm,^1^ and while global adverse birth outcomes continue to decline due to investments into antenatal health interventions in low- and middle-income countries, progress has stagnated, and rates are rising in many higher-income settings.^4^^,^^5^ Japan, despite its renowned maternal and child health system and under-five mortality rate of 2.36 per 1,000,^5^ faces growing concern regarding a persistently high prevalence of low birth weight, the second highest in the OECD at 9.4% in 2022, and PTB, which rose until stabilizing around 2008.^6^

Global surface temperatures have increased substantially over recent decades, raising concern that climate change may increasingly influence pregnancy outcomes. Non-optimal temperatures (heat and cold) may trigger PTB through thermoregulatory and biochemical pathways, particularly in late pregnancy when fetal growth demands peak, disrupting uteroplacental function and hormonal regulation, increasing the risk of PTB.^7^, ^8^, ^9^ In recent years, there has been mounting global evidence that exposure to non-optimal temperatures, particularly extreme heat, can exacerbate risks of PTB, especially in the last several weeks of pregnancy, which is particularly concerning in the context of climate change. The most recent systematic reviews have found 12% increased odds (95% confidence interval [CI]: 1.06–1.18) of PTB at high heat exposure at up to 28 days before birth (n = 39 studies), although substantial between-study heterogeneity, measured by the I-squared index (*I*^*2*^), was reported (*I*^*2*^: 92%),^10^ and a more limited number of studies (n = 5) showing a 16% (95% CI: 1.12–1.20, *I*^*2*^: 39%) increased risk of PTB with cold exposure at the last trimester of pregnancy.^9^ This heterogeneity likely reflects differences in exposure definitions, climatic contexts, and study design, highlighting important limitations of the existing evidence base.

Furthermore, despite this growing body of evidence, few epidemiological studies have assessed risks across the full temperature spectrum, particularly for cold exposure, in the Japanese population, or over extended time periods. Only one study has been conducted directly on the association between ambient temperature and PTB within Japan (excluding Okinawa) which found 8% increased heat-related risk of PTB (95% CI: 1.00–1.17) and 15% increased cold-related risk (95% CI: 1.05–1.25),^11^ and one single city-study in Brisbane, Australia examined temporal changes in the susceptibility to temperature related PTB, finding attenuated heat-related risk and strengthened cold-related PTB risk between 1994–2013.^12^ However, given that few countries have maintained longitudinal birth records over decades, no nationally representative analyses of temporal changes in temperature-related PTB risk have been conducted to date. Evidence from other health outcomes suggests that temperature-related risks do not necessarily decline monotonically over time, with some studies reporting early reductions followed by stagnation, highlighting potential limits to population-level adaptation.^13^

Japan offers a uniquely valuable setting for conducting a long-term, nationwide temporal and spatial investigation, having maintained a standardized system of maternal and antenatal health services since 1965, collecting high-quality birth records for more than four decades. This provides an opportunity to generate global relevant evidence, given the scarcity of settings where temporal changes in climate-related health risks can be rigorously examined. Moreover, amid Japan’s low fertility and growing rates of adverse newborn health indicators, there remains significant impetus to investigate environmental influences on PTB from a long-term perspective. Therefore, the objective of this study was to assess the temporal and spatial variation in the association between ambient temperature during late-pregnancy and PTB across all 47 prefectures in Japan.

## Methods

### Study design

We utilized a two-stage study design to evaluate the short-term association between daily mean temperature and PTB. At the first stage, we estimated the relative risks (RR) and associated 95% CI of PTB at the prefecture-level before pooling the reduced coefficients at the second stage to calculate national-level heat- and cold-related RRs. To test for temporal variation, subperiod analyses with the same two-stage modeling were conducted by dividing the data into four decadal subperiods (1979–1989, 1990–2000, 2001–2011, 2012–2023). Linear mixed-effects meta-regression was further applied to examine the role of prefecture-level variables in explaining temporal differences in the minimum morbidity temperature (MMT), and heat- and cold-related PTB risks.

### Data collection

Vital statistics data for all 47 prefectures of Japan from January 1, 1979, to December 31, 2023, were obtained from the Ministry of Health, Labour, and Welfare.^14^ Records with missing date of birth, missing or implausible gestational age (<22 or >44 weeks) or weight (<500 g), and birthplace outside of Japan were excluded; data cleaning steps and the resulting analytical sample are summarized in Supplementary Fig. S1. We extracted the infant’s birth date, gestational age, prefecture of birth, sex assigned at birth, paternal ages, and parity for the analysis. Our analyses were restricted to singleton births due to differential risk of PTB in multiple pregnancies which may confound further analyses.^15^ PTB severity was categorized as moderate to late preterm (32–36 gestational weeks), very preterm (28–31 gestational weeks), and extremely preterm (<28 gestational weeks), per World Health Organization definitions.^16^

Daily 24-h mean ambient temperature (Celsius (°C)) and relative humidity (%) were obtained for each prefecture from the Japan Meteorological Agency. These data were obtained by averaging hourly measurements from weather stations at the prefectural capital to adequately capture prefecture-wide weather patterns.

To explore how time-varying factors could influence PTB susceptibility to non-optimal temperature across the observational period, we also compiled a range of prefecture-level variables (Supplementary Table S1) including demographic and socioeconomic measures (population, births, income and household savings), health systems capacity metrics (number of doctors, nurses, hospitals, and hospital beds per capita), and household air conditioning (A/C) prevalence. Subperiod averages for each of these metrics were calculated for use in analyses.

### Statistical analysis

At the first stage, a time-stratified case-crossover study design with a conditional quasi-Poisson regression model was used to estimate prefecture-specific associations between daily mean temperature and PTB. This design was chosen because it allows for the estimation of short-term temperature associations while controlling for seasonality, long-term trends, and slowly varying confounders through time stratification, without requiring explicit modeling of the baseline risk. Quasi-Poisson regression models were used to account for overdispersion in daily prefecture-level PTB counts. The model structure was specified as follows:

$$\ln\left[\mu_{p,i,s}\right]=\alpha_{p,s}+\beta_{p}^{T}\sum_{l=0}^{28}W\left(T_{p,i-l},l\right)+{\gamma_{p}X}_{\text{Holiday},i}$$

where $\mu_{p,i,s}=E\left[Y_{p,i,s}\;|\text{covariates}\;\text{within}\;\text{prefecture}\;p\;\text{and}\;\text{stratum}\;s\right]$ denotes the conditional expectation of PTB counts $Y_{p,i,s}$ in prefecture p on day i with stratum s, $T_{p,i}$ represents the mean temperature, $X_{\text{Holiday},i}$ a binary dummy variable indicating national holidays in Japan with $\gamma_{p}$ of the corresponding regression coefficient parameter, and $\alpha_{p,s}$ is the stratum-specific intercept parameter. Under the mean-variance assumption of quasi-Poisson, the conditional variance of $Y_{p,i,s}$ on covariates is specified as $\phi_{p}\mu_{p,i,s}$ with the dispersion parameter $\phi_{p}$. The stratum s was defined as the interaction between the day of the week (DOW), month, and year to adjust the model for the DOW, seasonality, and long-term trends. W(T,l) denotes a nine by one cross-basis function vector consisting of three basis functions for temperature T and three basis functions for lag period l, specifying a distributed lag non-linear model (DLNM).^17^ For the exposure-response curve, we used a natural cubic spline with two knots at the 33rd and 66th percentiles of the prefecture-specific temperature distribution, and a natural cubic spline with two internal knots placed at equally spaced values in the log scale was used for the lag-response curve. To assess sensitivity to knot placement, we evaluated multiple alternative specifications for the temperature spline, including internal knots at the 50th percentile; at the 33rd and 66th percentiles; at the 25th, 50th, and 75th percentiles; and at the 10th, 75th, and 90th percentiles of the prefecture-specific temperature distribution. The final knot specification was selected from model performance based on the quasi-Akaike information criterion (QAIC) (Supplementary Table S4). $\beta_{p}$ is the corresponding nine by one coefficient vector. We set a lag period of 28 days prior to birth based on literature review,^10^ that we confirmed through exploratory analyses showing that PTB risk associated with non-optimal temperatures attenuated within this period (Supplementary Fig. S2). Heat-related PTB risk attenuated rapidly within approximately the first 10 days, while cold-related effects persisted for up to approximately four weeks. This lag length was therefore sufficient to capture both heat and cold effects within a unified modeling framework.

At the second stage, a random-effects meta-analysis based on the quasi-maximum likelihood was used to pool the prefecture-specific estimates ${\hat{\beta }}_{p}$ and obtain the national level estimates $\hat{\beta }$, and to calculate the best linear unbiased prediction (BLUP) for each prefecture. The *I*^*2*^ was used to assess between-prefecture heterogeneity. The MMT denoted as $T_{\text{MMT}}$ was obtained from the minimum of the nationally pooled exposure-response curve: $\exp\left\{\;{\hat{\beta }}^{T}\sum_{l=0}^{28}W\left(T,l\right)+\text{Constant}\right\}$. The national-level RR function of temperature T compared with MMT is then defined as:

$$\text{RR}\left(T\right)=\exp\left\{{\hat{\beta }}^{T}\left[\sum_{l=0}^{28}W\left(T,l\right)-\sum_{l=0}^{28}W\left(T_{\text{MMT}},l\right)\right]\right\}$$

For RR estimation, “heat” and “cold” were defined at the 99th and 1st percentiles of the mean temperature distribution relative to the MMT, respectively; corresponding RRs were estimated at $\text{RR}\left(T^{99\text{th}}\right)$ and $\text{RR}\left(T^{1\text{st}}\right)$. Prefecture-level MMTs were obtained in the same way using the BLUP with a search restriction from the 1st to 99th percentile of their prefecture specific temperature distribution. MMT uncertainty intervals were estimated with parametric bootstrapping, which identified the MMT of simulated spline coefficient sets (n = 10,000) from the estimated covariance matrix, and derived the 95% confidence interval and standard error from the resulting empirical distribution.^18^ Attributable fractions (AF) and attributable numbers (AN) were estimated using a forward perspective, quantifying the proportion and number of PTB cases attributable to non-optimal temperature exposure. Using RRs for the entire lag period, daily contributions from temperatures above and below the prefecture-specific MMT were summed to estimate heat- and cold-associated burdens^19^; this is in contrast to percentile-based RR estimation. The CIs of AF values were then estimated empirically with Monte Carlo simulations (n = 1000), sampling the cross-basis coefficients from its assumed normal distribution based on the model’s estimates and covariance matrix. To identify vulnerable groups with regards to the temperature-PTB association, subgroup analyses were also conducted for several key factors including PTB severity, parity, parental ages, sex, and region. The *p*-values for differences between two RR estimates were obtained from the *Z*-test comparing the difference between the log-transformed estimates to the combined standard error.^20^

Finally, univariate mixed-effects meta-regression models were used to examine how prefecture-level, time-varying predictors (averaged by subperiod) modified the association between non-optimal temperature and PTB risk. Each predictor was modeled as a fixed effect nested in prefecture.^21^ Separate models were fitted for each outcome (heat- and cold-related RR, and MMT). Coefficients for each predictor variable were standardized per one standard deviation (SD) for comparability of effect estimates. Results were reported as the change in log-RR or MMT per one SD increase in the predictor, along with 95% CI and *p*-values (significance was set at *p* < 0.01 to account for multiple comparisons). These models were used to explore how prefecture-level variables may contribute to temporal differences in heat- and cold-related PTB risk and MMT; however, because predictors were examined in separate univariate models, the results should be interpreted as exploratory and not as evidence of independent causal effects.

All analyses were performed in R (version 4.3.1; R Development Core Team) with the *dlnm* and *mixmeta* packages.

### Sensitivity analysis

For sensitivity analysis, adjustment for humidity as a time-varying confounder in the main model was tested using a three-day moving average for relative humidity modeled with a natural cubic spline with three degrees of freedom. Two subperiods of about 20 years (1979–2000, 2000–2023) were also alternatively tested for subperiod analysis.

### Role of funding source

The funders of this project had no role in study design, data collection, data analysis, interpretation, or writing of the report.

### Ethics approval

The Ethics Review Committee for Life Science and Medical Research Involving Human Subjects of the National Institute of Infectious Diseases formally evaluated this study (No. 1909) and issued a documented exemption on February 26, 2025, as this study utilized secondary, anonymized data that did not contain any personal identifiers, and no direct interaction with human participants occurred. Individual informed consent was therefore not required.

## Results

Across the 45-year study period, 2,604,733 singleton PTBs were identified, representing 4.94% of 52,749,360 live births. Descriptive statistics for meteorological variables, births, and subgroup distributions are shown in Table 1. Mean temperature increased by 1.36 °C from 14.61 °C in 1979–1989 to 15.97 °C in 2012–2023. PTB prevalence rose from 4.16% (n = 669,499) to 5.53% (n = 674,655) by 2001–2011, then stabilized at 5.49% (n = 610,168). The modest decline in extremely and very PTBs indicated that rising prevalence was mainly driven by moderate to late PTBs, rising from 87.42% of PTB cases in 1979–1989 to 88.87% in 2012–2023. The proportion of mothers aged ≥35 years increased from 10.59% (n = 70,908) to 34.32% (n = 209,385), reflecting demographic shifts in Japan’s reproductive population (see Supplementary Table S2).

**Table 1:** Summary statistics of subperiod stratified meteorological data, birth data, and preterm birth subgroups in Japan, 1979–2023.

Measure	Overall (1979–2023)	1979–1989	1990–2000	2001–2011	2012–2023
Meteorological data					
Mean temperature (°C)	15.40 (8.62)	14.61 (8.59)	15.39 (8.49)	15.57 (8.65)	15.97 (8.69)
1st percentile	−2.00	−2.80	−1.60	−1.90	−1.80
99th percentile	30.20	29.20	30.10	30.20	30.60
Mean relative humidity (%)	69.89 (12.56)	70.79 (12.56)	70.10 (12.43)	68.43 (12.20)	70.21 (12.88)
Birth data					
Total live births	52,749,360 (100%)	16,088,250 (100%)	13,354,755 (100%)	12,200,706 (100%)	11,105,649 (100%)
Term singleton births	49,655,616 (94.14%)	15,278,188 (94.96%)	12,577,936 (94.18%)	11,412,275 (93.54%)	10,387,217 (93.53%)
Preterm singleton births	2,604,733 (4.94%)	669,499 (4.16%)	650,411 (4.87%)	674,655 (5.53%)	610,168 (5.49%)
Subgroup distribution of preterm births (PTB)					
PTB severity					
Extremely PTB	94,445 (3.63%)	23,335 (3.49%)	25,371 (3.90%)	25,121 (3.72%)	20,618 (3.38%)
Very PTB	219,680 (8.43%)	60,910 (9.10%)	56,873 (8.74%)	54,574 (8.09%)	47,323 (7.76%)
Moderate to late PTB	2,290,608 (87.94%)	585,254 (87.42%)	568,167 (87.36%)	594,960 (88.19%)	542,227 (88.87%)
Infant sex					
Male	1,475,855 (56.66%)	382,084 (57.07%)	370,100 (56.90%)	378,774 (56.14%)	344,897 (56.52%)
Female	1,128,878 (43.34%)	287,415 (42.93%)	280,311 (43.10%)	295,881 (43.86%)	265,271 (43.48%)
Maternal age					
35+	530,146 (20.35%)	70,908 (10.59%)	91,094 (14.01%)	158,759 (23.53%)	209,385 (34.32%)
25–34	1,706,961 (65.53%)	469,395 (70.11%)	451,639 (69.44%)	435,833 (64.60%)	350,094 (57.38%)
15–24	367,342 (14.10%)	129,135 (19.29%)	107,609 (16.54%)	79,981 (11.86%)	50,617 (8.30%)
Paternal age					
35+	839,016 (32.21%)	152,895 (22.84%)	189,132 (29.08%)	232,444 (34.45%)	264,545 (43.36%)
25–34	1,496,096 (57.44%)	446,720 (66.72%)	383,705 (58.99%)	370,746 (54.95%)	294,925 (48.34%)
15–24	193,801 (7.44%)	53,940 (8.06%)	60,934 (9.37%)	48,901 (7.25%)	30,026 (4.92%)
Regions					
Northern Japan	1,546,757 (59.38%)	385,770 (57.62%)	385,577 (59.28%)	405,952 (60.17%)	369,458 (60.55%)
Southern Japan	1,057,976 (40.62%)	283,729 (42.38%)	264,834 (40.72%)	268,703 (39.83%)	240,710 (39.45%)
Parity					
Primiparous	1,100,699 (42.26%)	266,589 (39.82%)	282,900 (43.50%)	291,233 (43.17%)	259,977 (42.61%)
Multiparous	1,504,034 (57.74%)	402,910 (60.18%)	367,511 (56.50%)	383,422 (56.83%)	350,191 (57.39%)

Note: Standard deviations are documented for mean temperature and relative humidity. For birth data, percentages are taken relative to total live births. For preterm birth subgroup distributions, percentages are taken relative to total preterm singleton births. Birth data were cleaned to remove births missing birth dates, missing or with abnormal gestational ages < 22 week and >44 weeks, location of birth outside of Japan, and abnormally low weights < 500 g. Preterm birth severity is divided into moderate to late preterm (32–36 gestational weeks), very preterm (28–31 gestational weeks), and extremely preterm (<28 gestational weeks) per WHO definitions. Northern Japan consists of Japanese Industrial Standard JIS X 0401 prefecture codes 01–23, and Southern Japan 24–47.

**Fig. 1:**
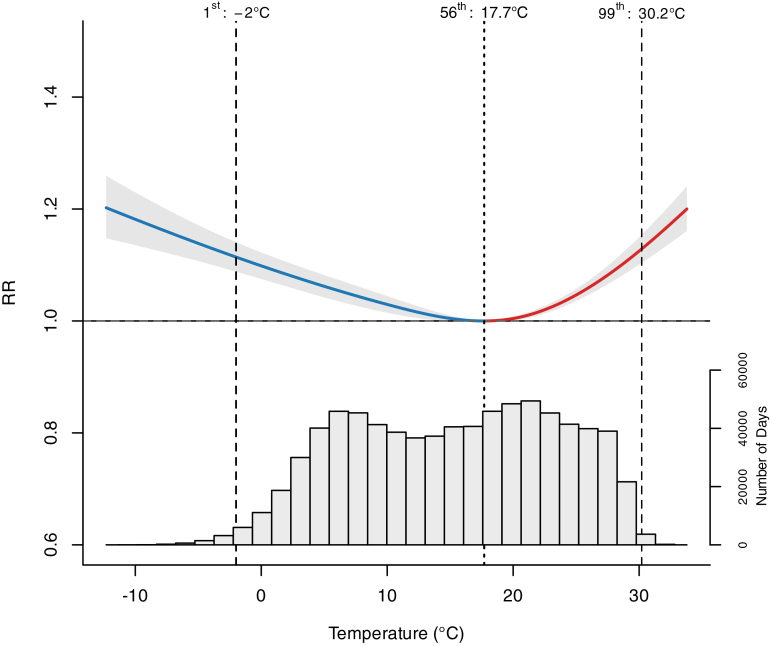
Lag-cumulative national pooled relative risk (RR) curve for mean temperature and preterm birth, with a histogram of the frequency of temperature events from 1979 to 2023 in Japan.

Fig. 1 shows the nationally pooled exposure-response curve, revealing a U-shaped relationship with an MMT at the 56th temperature percentile (17.7 °C), consistent with the previous study in Japan.^11^ The heat-related RR was 1.13 [95% CI 1.10–1.15; AF: 1.9% (1.7–2.1%)], and the cold-related RR was 1.11 [95% CI 1.09–1.14; AF: 2.8% (2.4–3.1%)]. Testing for heterogeneity, the *I*^*2*^ was 24.9%. Sensitivity analyses including relative humidity showed negligible influence on main and subperiod models; detailed estimates and model optimization results are presented in Supplementary Tables S3 and S4.

Table 2 summarizes the pooled, national-level results of the mean temperature-PTB relationship (MMT, cold-related RR, and heat-related RR) across subperiods and subgroups. For the subperiod analysis, a notable decline in heat-related RR was observed from 1.16 [95% CI: 1.12–1.21] in 1979–1989 to 1.07 [95% CI: 1.03–1.12; *p*: 0.0084] in 2001–2011 and plateauing at 1.09 [95% CI: 1.05–1.14; *p*: 0.039] in 2012–2023, while cold-related RR did not significantly change. The absolute value of the MMT remained relatively stable from 17.8 °C in 1979–1989 to 17.6 °C in 2012–2023, but the MMT percentile decreased from the 59th percentile to the 53rd percentile as the overall temperature distribution shifted higher.

**Table 2:** Minimum morbidity temperature percentile (MMTP), minimum morbidity temperature (MMT), cold- and heat-related relative risks (RR) stratified by subperiods and subgroups with 95% confidence intervals (CI).

Subgroup	*I*^*2*^	MMT		Cold-related		Heat-related	
		Percentile (95% CI)	MMT (°C) (95% CI)	RR (95% CI)	*p*-value for difference	RR (95% CI)	*p*-value for difference
Main model (1979–2023)	24.9%	56th (51st–60th)	17.70 (16.20–18.70)	1.11 (1.09–1.14)	–	1.13 (1.10–1.15)	–
Subperiods							
1979–1989	9.1%	59th (54th–63rd)	17.80 (16.30–18.80)	1.11 (1.06–1.16)	Reference	1.16 (1.12–1.21)	Reference
1990–2000	5.2%	49th (37th–59th)	15.60 (11.70–18.30)	1.10 (1.05–1.14)	0.38	1.13 (1.09–1.18)	0.26
2001–2011	1.4%	55th (40th–66th)	17.60 (12.80–20.40)	1.11 (1.06–1.16)	0.40	1.07 (1.03–1.12)	**0.0084∗**
2012–2023	0.0%	53rd (40th–61st)	17.40 (13.50–19.80)	1.14 (1.09–1.18)	0.27	1.09 (1.05–1.14)	**0.039∗**
Subgroups							
Severity							
Extremely PTB	0.4%	1st (1st–99th)	−2.00 (−2.00–30.20)	1.00 (1.00–1.00)	Reference	1.06 (0.91–1.23)	Reference
Very PTB	0.0%	1st (1st–99th)	−2.00 (−2.00–30.20)	1.00 (1.00–1.00)	N/A	1.05 (0.94–1.18)	0.40
Moderate to late PTB	23.2%	55th (51st–59th)	17.40 (16.10–18.40)	1.14 (1.11–1.17)	**<0.0001∗**	1.15 (1.12–1.18)	0.23
Infant sex							
Male	14.1%	55th (48th–61st)	17.40 (15.30–18.90)	1.09 (1.06–1.12)	Reference	1.11 (1.08–1.15)	Reference
Female	14.0%	56th (49th–61st)	17.70 (15.70–19.10)	1.15 (1.11–1.18)	**0.015∗**	1.15 (1.11–1.19)	0.13
Maternal age							
35+	0.0%	44th (31st–99th)	14.10 (9.60–30.20)	1.08 (1.04–1.12)	Reference	1.03 (0.98–1.08)	Reference
25–34	23.0%	57th (52nd–60th)	17.90 (16.40–18.80)	1.12 (1.09–1.16)	0.14	1.14 (1.11–1.17)	**<0.0001∗**
15–24	0.0%	55th (47th–61st)	17.40 (15.00–18.90)	1.12 (1.07–1.19)	0.22	1.23 (1.16–1.30)	**<0.0001∗**
Paternal age							
35+	0.0%	54th (42nd–62nd)	17.10 (13.40–19.20)	1.12 (1.08–1.17)	Reference	1.09 (1.05–1.13)	Reference
25–34	20.0%	58th (51st–62nd)	18.20 (16.30–19.30)	1.12 (1.09–1.15)	0.40	1.13 (1.10–1.17)	0.13
15–24	0.0%	51st (1st–60th)	16.20 (−2.00–18.60)	1.06 (0.99–1.14)	0.16	1.26 (1.17–1.37)	**0.0019∗**
Region							
Northern Japan	40.7%	57th (49th–62nd)	17.90 (15.60–19.30)	1.13 (1.09–1.17)	Reference	1.15 (1.11–1.19)	Reference
Southern Japan	0.0%	54th (45th–61st)	17.10 (14.30–19.00)	1.10 (1.06–1.14)	0.23	1.11 (1.07–1.14)	0.14
Parity							
Primiparous	19.9%	59th (52nd–63rd)	18.50 (16.60–19.60)	1.14 (1.10–1.17)	Reference	1.12 (1.09–1.16)	Reference
Multiparous	12.6%	53rd (44th–59th)	16.80 (13.90–18.40)	1.09 (1.06–1.13)	0.056	1.13 (1.10–1.17)	0.37

Note: RR estimates are presented with 95% confidence intervals (CI). *p*-values for difference were considered significant *p* < 0.05. Statistically significant p-values were bolded and noted with an asterisk. Preterm birth severity is divided into moderate to late preterm (32–36 gestational weeks), very preterm (28–31 gestational weeks), and extremely preterm (<28 gestational weeks) per WHO definitions. Northern Japan consists of Japanese Industrial Standard JIS X 0401 prefecture codes 01–23, and Southern Japan 24–47.

Subgroup analyses revealed heterogeneity in vulnerability to non-optimal temperatures across severity of PTB and parental demographic characteristics. Significant vulnerability to non-optimal temperatures was observed among moderate to late PTBs, while insignificant for very or extremely PTB subgroups. Compared to male infants [RR = 1.09, 95% CI: 1.06–1.12], female infants showed greater cold-related susceptibility [RR = 1.15, 95% CI: 1.11–1.18; *p*: 0.015]. Regarding parental age, younger mothers and fathers showed significantly higher susceptibility to heat exposure. The heat-related risk of PTB was highest for mothers aged 15–24 years [RR = 1.23, 95% CI: 1.16–1.30; *p*: < 0.0001] compared to for mothers aged ≥35 years [RR = 1.03, 95% CI: 0.98–1.08]. Similarly for paternal age, the highest heat-related risk was in fathers aged 15–24 years [RR = 1.26, 95% CI: 1.17–1.37; *p*: 0.0019] compared to fathers aged ≥35 years [RR = 1.11, 95% CI: 1.09–1.13]. Exposure-response curves illustrating these subgroup-specific associations are presented in Supplementary Fig. S3.

Supplementary Table S5 and Supplementary Fig. S4 present the AFs and corresponding ANs of PTBs associated with heat, cold, and total non-optimal temperatures, stratified by subperiod. Overall, an estimated 102,266 PTBs were attributable to non-optimal temperatures, corresponding to a total AF of 4.7% [95% CI: 4.3–5.0%]. Of these, 41,739 cases were attributed to heat exposure [heat-related AF: 1.9%, 95% CI: 1.7–2.1%], and 60,527 cases to cold exposure [cold-related AF: 2.8%, 95% CI: 2.5–3.0%]. A declining trend was observed, with a heat-related AF of 2.3% [95% CI: 2.0–2.6%] in 1979–1989, compared to 1.5% [95% CI: 1.3–1.7%] in 2012–2023.

**Fig. 2:**
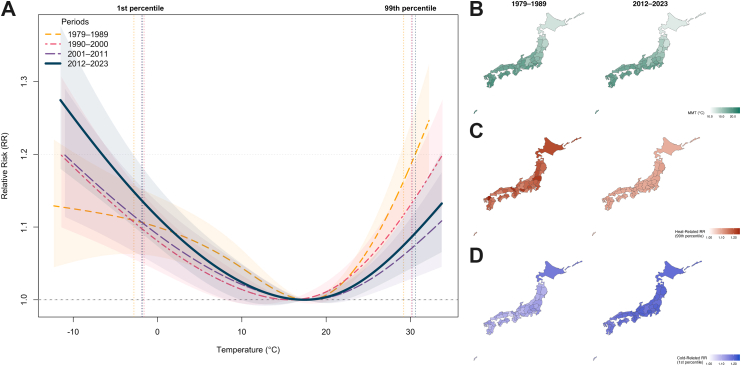
(*A*) Lag-cumulative relative risk (RR) curves for mean temperature and preterm birth stratified by time periods (1979–1989, 1990–2000, 2001–2011, 2012–2023). (*B*) Prefecture-specific minimum morbidity temperature (MMT) in 1979–1989 and 2012–2023. (*C*) Prefecture-specific heat-related RR in 1979–1989 and 2012–2023. (*D*) Prefecture-specific cold-related RR in 1979–1989 and 2012–2023.

Subperiod stratified temperature-PTB RR curves are illustrated in Fig. 2A, and the spatial distribution of MMTs and the heat- and cold-related RRs across Japan are mapped in Fig. 2B–D. Overall, RRs from extreme heat (>30 °C) to moderate cold (5 °C) decreased in the most recent subperiod, consistent with the significant reduction in heat-related AFs. A modest increase in risk at very cold temperatures (<5 °C) was observed in more recent decades compared with earlier periods; however, the CIs were wide, and differences in the RRs at the 1st percentile and in the overall cold-related AFs were not statistically significant. Spatially, changes in heat- and cold-related RRs appeared relatively homogeneous across prefectures, suggesting consistent temporal patterns nationwide. MMTs remained stable between the two periods, with a north-to-south gradient of increasing MMTs reflecting expected climatic variation.

Table 3 presents the univariate associations between prefecture-level time-varying factors and changes in the MMT, heat- and cold-related risks. Among all predictors evaluated, climate variables and A/C prevalence were significantly associated with MMT variation [*p* < 0.01]. Mean, maximum, and minimum temperatures were each independently positively associated with higher MMTs [β per SD increase: 2.05, 95% CI: 1.74–2.36; 2.07, 1.75–2.40; and 1.96, 1.67–2.26, respectively; all *p* < 0.0001], whereas temperature range demonstrated a negative association [−1.68, −1.96 to −1.41; *p* < 0.0001] indicating that regions with narrower temperature variability tended to have higher MMTs. A/C prevalence was also positively associated with the MMT (1.98, 1.23–2.72; *p* < 0.0001), suggesting that greater access to cooling may have shifted the temperature-PTB exposure-response curve rightwards.

**Table 3:** Associations between prefectural meta-predictors and minimum morbidity temperature (MMT), and cold- and heat-related relative risks (RR) with 95% confidence intervals (CI).

Meta-predictor	MMT		Cold-related RR		Heat-related RR	
	Coefficient (95% CI)	*p*-value	Coefficient (95% CI)	*p*-value	Coefficient (95% CI)	*p*-value
Meteorological						
Mean temperature	2.050 (1.742, 2.358)	**<0.0001∗**	−0.007 (−0.014, −0.000)	0.045	0.001 (−0.005, 0.006)	0.78
Maximum temperature	2.071 (1.745, 2.396)	**<0.0001∗**	−0.006 (−0.013, 0.000)	0.067	0.002 (−0.004, 0.007)	0.54
Minimum temperature	1.963 (1.666, 2.260)	**<0.0001∗**	−0.007 (−0.013, −0.000)	0.040	0.000 (−0.005, 0.006)	0.96
Temperature range	−1.682 (−1.959, −1.405)	**<0.0001∗**	0.004 (−0.003, 0.010)	0.25	0.001 (−0.005, 0.006)	0.78
Relative humidity	−0.429 (−0.972, 0.114)	0.12	−0.001 (−0.007, 0.006)	0.85	0.001 (−0.005, 0.006)	0.82
Demographic						
Population	0.121 (−0.438, 0.680)	0.67	0.000 (−0.006, 0.007)	0.92	−0.001 (−0.006, 0.004)	0.79
Births	0.175 (−0.436, 0.786)	0.58	−0.001 (−0.007, 0.006)	0.88	−0.001 (−0.007, 0.005)	0.74
Medical resources						
Hospitals	0.370 (−0.214, 0.955)	0.21	−0.004 (−0.011, 0.003)	0.26	0.001 (−0.004, 0.007)	0.67
Hospital beds	0.208 (−0.369, 0.784)	0.48	−0.004 (−0.011, 0.002)	0.19	0.002 (−0.004, 0.007)	0.57
Doctors	0.776 (−0.014, 1.565)	0.054	−0.008 (−0.018, 0.002)	0.10	0.002 (−0.006, 0.010)	0.62
Nurses	0.347 (−0.668, 1.362)	0.50	−0.011 (−0.024, 0.003)	0.11	−0.001 (−0.013, 0.011)	0.84
Socioeconomic						
Income	−0.794 (−1.456, −0.131)	0.019	0.008 (−0.002, 0.017)	0.10	0.010 (0.002, 0.017)	0.011
Savings	−1.438 (−2.649, −0.226)	0.020	0.014 (−0.003, 0.031)	0.11	0.016 (0.003, 0.029)	0.017
Air conditioning	1.975 (1.232, 2.717)	**<0.0001∗**	−0.004 (−0.015, 0.006)	0.44	0.002 (−0.005, 0.010)	0.54

Note: Estimates are presented as the change in log-RR or MMT per one standard deviation increase in the predictor, along with 95% confidence intervals (CI) and *p*-values. Statistical significance was defined as *p* < 0.01 to account for multiple comparisons. Statistically significant *p*-values were bolded and noted with an asterisk.

In the sensitivity analysis, the results for the model with two longer subperiods (1979–2000 and 2001–2023) were consistent with the trends from the main model. The heat-related RR declined from 1.15 [95% CI: 1.11–1.18] in 1979–2000 to 1.09 [95% CI: 1.05–1.12; *p*: 0.024] in 2001–2023, indicating the same pattern of reduced heat-related risk over time. The cold-related RR remained largely unchanged between the two periods, similar to the main model. In the accompanying meta-regression of time-varying predictors, climate variables and AC prevalence remained significantly associated with the MMT. Full results from this two-period sensitivity analysis are provided in Supplementary Fig. S5, and Supplementary Tables S6 and S7.

## Discussion

This study provides the first nationally representative assessment of long-term temporal changes in temperature-related PTB risk over four decades (1979–2023) in Japan. Using a widely applied and robust time-stratified case-crossover framework, this analysis extends prior work by examining both heat- and cold-related PTB risks over an extended national time series, addressing key gaps in the existing literature. Given Japan’s exposure to both heat and cold extremes, these findings can be relevant to a wide range of climatic contexts. We observed a decline in the heat-related PTB risk and AF across all prefectures, followed by a plateau since the 2000s. While early reductions parallel declines in heat-related mortality observed in Japan and other high-income countries, where adaptation and public health measures have reduced mortality risks,^22^^,^^23^ this progress appears to have stalled for PTB in recent decades. In Japan specifically, heat-related mortality risk declined to near negligible levels in recent decades, with the AF falling to 0.5%.^24^ In contrast, pregnant individuals appear to have benefited less from these population-level gains, as heat-related PTB risk persisted despite broader population adaptation. This divergence suggests that existing heat adaptation strategies may have reached their limits for protecting pregnant individuals, despite access to a highly advanced and accessible maternal health system and gains for other populations.

The same temporal reduction was not observed for cold-related PTB risk or MMT. Although MMTs remained stable, declining MMT percentiles suggest that adaptation has not kept pace with rising temperatures. Positive associations between MMT and climatic indicators, as well as with A/C prevalence in univariate analyses, would be consistent with partial heat adaptation and a rightward shift of the exposure-response relationship, as seen in Brisbane.^12^ The lack of a corresponding rise in the MMT or decline in cold-related risk indicates limited adaptation to cold temperatures. Behavioral and infrastructural factors, such as long-established clothing and heating norms, may have constrained further improvements.^13^ Social vulnerability factors (e.g., increased maternal employment) may have also increased exposure to outdoor cold. Although prefectural income and savings showed positive associations with heat-related PTB risk in univariate meta-regressions [β per SD increase: 0.010, 95% CI: 0.002–0.017; 0.016, 0.003–0.029; *p*: 0.011 and 0.017, respectively], these did not reach our pre-specified significance threshold of *p* < 0.01. Higher-income prefectures tend to be more urbanized, and are thus subject to greater urban heat island effects,^25^ with commuting patterns involving more walking and public transit use that may increase personal outdoor heat exposure.^26^ These features of the urban environment, rather than a direct socioeconomic effect, may explain the observed direction of association. Taken together, these findings suggest that while heat-related PTB risk has declined over time, likely reflecting multiple adaptive processes, susceptibility to cold-related PTB has remained largely unchanged.

Stratified subgroup analyses identified several populations with increased vulnerability to non-optimal temperatures. The association between temperature and PTB was most evident for moderate to late PTBs, whereas very and extremely PTBs showed no significant association. This is physiologically plausible, as earlier PTBs are more likely driven by non-environmental etiologies such as obstetric complications and infections,^27^ while late PTBs may be more sensitive to acute environmental stressors that precipitate labor through endocrine or vascular mechanisms.^8^ Parental age also modified temperature-related susceptibility, with younger parents showing greater heat-related risk, possibly reflecting socioeconomic, occupational, or educational disparities influencing exposure patterns, awareness, and access to adaptive resources such as cooling and healthcare.

Female infants also exhibited higher cold-related vulnerability than males which is consistent with a limited number of previous studies which have found higher cold-related PTB risk for female infants despite higher overall higher baseline PTB risk for male infants.^28^^,^^29^ This may be related to sex-specific fetal evolutionary strategies, where female fetuses’ have higher adaptive capacity, and male fetuses prioritize faster growth even under environmental stress, rendering them more prone to fetal death.^30^ Consequently, female infants may be overrepresented among PTBs following temperature extremes, given they are more likely to survive to delivery following environmental insult.

### Implications

These findings highlight pregnant women as an underrecognized vulnerable population in climate adaptation planning, with consequences that extend beyond childbirth. Importantly, PTB is associated with long-term adverse outcomes for children, including impaired neurodevelopment, cardiometabolic disease, and reduced life-course health, indicating that climate-related PTB risk may have enduring intergenerational consequences.^2^ The lack of improvement in heat-related PTB risk since the 2000s, together with the persistence of cold-related risk, even in Japan, a high-income country with universal health insurance coverage, high A/C prevalence, and heat adaptation policies (e.g., the national Heat Stroke Alert system implemented in 2011),^13^ suggests that current population-level strategies insufficiently address the physiological vulnerability and exposure contexts of pregnancy. Unlike older adults, adaptation interventions have not been tailored for expectant mothers. Despite national progress in reducing heat-related mortality in Japan, maternal health remains absent from Japan’s national climate adaptation plan,^31^ and physician education on planetary health is limited.^32^ Globally, nearly half of national heat-health action plans omit pregnant women.^33^ While Japan’s disaster guidelines have started to recognize pregnant women as a vulnerable group,^34^ broader public and policy recognition is urgently needed.

Strengthening maternal climate resilience will require coordinated strategies across multiple sectors. These include training medical professionals on temperature-related risks and integrating counseling into antenatal care through Japan’s maternal and child health handbooks and home-visit programs. Beyond healthcare, expanding adaptive infrastructure, such as mitigating urban heat islands with shade and green space, improving building insulation, and subsidizing home cooling and heating for low-income households who are more reluctant to use A/C,^13^ could reduce exposure. Stricter occupational regulations, including shorter shifts, flexible hours, and remote work during extreme weather, may also protect pregnant workers. Embedding these measures into Japan’s existing climate and health adaptation programs can provide a framework to safeguard maternal and fetal health under climate change.

Subgroup differences highlight inequities in adaptive capacity shaped by broader social and structural determinants of health. Younger parents may face greater occupational or outdoor exposure, while older, financially secure parents may benefit from better housing and stable employment. These disparities show how socioeconomic vulnerability intersects with biological sensitivity to amplify risks among groups lacking the capacity to mitigate exposure. Targeted adaptation strategies, such as integrating climate risk screening into maternal health programs and expanding social protection for younger and lower-income families, could reduce temperature-related disparities and strengthen equity-focused climate adaptation.

### Limitations

With regards to limitations, first, although the time-stratified case-crossover design controls for time-invariant confounders, bias from time-varying factors should be considered. Consistent air pollution data were unavailable for the full 1979–2023 period, and given potential mediation between temperature and pollutants, their inclusion could have introduced bias or instability in multipollutant lag models. Because our focus was on the temperature-attributable burden of PTB and its temporal changes, we excluded pollutants to preserve causal interpretability and policy relevance. Second, the time-stratified case-crossover design relies on assumptions that should be considered in the context of PTB. In particular, the baseline risk of PTB varies with gestational age and the occurrence of a PTB precludes subsequent events within the same pregnancy. Although these assumptions do not strictly hold at the individual level, our analysis was conducted using aggregated daily prefecture-level counts, which mitigates, though does not eliminate, potential bias by averaging risk across large populations and time strata. Resulting bias is therefore more likely to attenuate short-term associations rather than produce spurious effects. Furthermore, our design defines strata based on the interaction of year, month, and DOW, ensuring that comparisons are made within one month. Within such a short period, the variation in baseline risk is considered to be small. Previous studies suggest that the bias resulting from the changing baseline risk is negligible in time-stratified designs with short strata.^35^ Thus, residual bias is unlikely to substantially alter the estimated short-term associations. Third, temperature exposures were assigned at the prefecture level, which may introduce Berkson-type measurement error.^36^ Such error is expected to increase uncertainty without systematically biasing effect estimates. Fourth, incomplete data on maternal characteristics limited subgroup analyses, and the univariate meta-regression approach constrained statistical power and model flexibility. As predictors were examined in separate univariate meta-regression models, observed univariate associations should also be interpreted cautiously and cannot be taken as evidence of independent causal adaptation mechanisms. Fifth, temporal variation was assessed using decadal subperiods rather than shorter time windows. While finer temporal stratification could capture more minute changes in susceptibility, further subdividing the data would substantially reduce statistical power for this relatively low-incidence outcome and result in unstable estimates. Therefore, our analysis prioritizes the identification of longer-term temporal patterns over short-term fluctuations, and more dynamic changes within shorter periods may not be fully captured. Finally, generalizability beyond Japan may be limited. Japan’s overall high prevalence of A/C may attenuate indoor heat exposure compared to populations in low- and middle-income settings, potentially resulting in lower estimates of heat-related PTB risk compared to those contexts.^37^ Japan’s high antenatal care coverage may also modify the relationship between temperature exposure and PTB by enabling earlier clinical intervention,^38^ which may not be replicable in settings with more limited obstetric infrastructure. Additionally, population-level vulnerability to PTB, which may be influenced by health factors such as infections,^39^ differs across settings in ways that could modify both the magnitude and direction of the temperature-PTB association. Findings should therefore be interpreted carefully when extrapolating to populations with substantially different climatic, socioeconomic, or health system contexts.

### Conclusion

Leveraging a national birth dataset spanning four decades (1979–2023), we found that while heat-related PTB risk and attributable burden have modestly declined, reflecting population-level adaptation to heat, cold-related risk has remained largely unchanged. Despite broader gains in heat resilience and declining mortality, expecting mothers have not experienced comparable protection, with the heat-related AF for PTB remaining several-fold higher than that for mortality. The plateauing of progress since the 2000s and persistent cold-related burden indicate that pregnant women remain a neglected population in climate adaptation planning. Integrating maternal health into resilience policies, through medical education, built environment investments, and occupational protections, could advance gender equality and health equity while informing globally relevant, gender-inclusive climate strategies.

## Contributors

Conceptualization: AC, LY, MH; Data curation: AC, SI, DY; Formal analysis: AC; Funding acquisition: MH; Methodology: AC, LY, MH; Project administration: MH; Resources: MH; Supervision: LY, MH; Validation: AC, LY; Visualization: AC, LY; Writing – original draft: AC; Writing – review & editing: all authors. AC, LY SI, and DY accessed and verified the underlying data. All authors have read and approved of the final version of this manuscript.

## Data sharing statement

Birth data analyzed in this study were provided by Japan’s Ministry of Health, Labour and Welfare under a restricted use agreement and cannot be shared by the authors. Researchers wishing to access these data may apply directly to the Vital, Health and Social Statistics Office of the Ministry of Health, Labour and Welfare of Japan.

## Declaration of interests

None.
