# Supplementary material for: Temporal trends in late-pregnancy exposure to ambient temperature and risk of preterm birth in Japan, a nationwide study from 1979 to 2023
Source: Lancet Reg Health West Pac. 2026 Jun 4;71:101894. doi: 10.1016/j.lanwpc.2026.101894 (PMC13254787; doi:10.1016/j.lanwpc.2026.101894)
Supplement: Supplementary Figs. S1–S7 and Tables S1–S7 [file mmc1.docx]

**Supplementary Material**

**Temporal trends in late-pregnancy exposure to ambient temperature and risk of preterm birth in Japan, a nationwide study from 1979–2023**

**Authors**

Alton Quan Cao, MHSc,^1*^ Lei Yuan, PhD,^1^ Sophearen Ith, PhD,^2,3^ Yui Tomo, PhD,^2^ Chris Fook Sheng Ng, PhD,^1^ Daisuke Yoneoka, PhD,^2^ Masahiro Hashizume, MD, PhD^1,4^

**Affiliations**

1. Department of Global Health Policy, Graduate School of Medicine, The University of Tokyo, Tokyo, Japan

2. Department of Epidemiology, National Institute of Infectious Diseases, Japan Institute for Health Security, Tokyo, Japan

3. Department of Global Environmental Health, Graduate School of Medicine, The University of Tokyo, Tokyo, Japan

4. Department of Global Health, School of Tropical Medicine and Global Health, Nagasaki University, Nagasaki, Japan

**Table of Contents:**

**Figure S1**: Live birth data inclusion criteria flow chart.

**Table S1**: List of prefectural meta-predictors.

**Table S2**: Subgroup summary statistics for documented live births in Japan, 1979-2023.

**Figure S2**: The lag-response curve for the hot- and cold-related relative risks (RR) of the temperature-preterm birth association.

**Table S3**: Minimum morbidity temperature percentile (MMTP), minimum morbidity temperature (MMT), cold- and heat-related relative risks (RR) with 95% confidence intervals (CI) stratified by subperiods for the sensitivity analysis model adjusting for relative humidity.

**Table S4**: Main model specification parameter search with quasi-Akaike information criterion (QAIC).

**Figure S3**: Lag-cumulative relative risk (RR) curves for mean temperature and preterm birth stratified by subgroups: (*A*) Preterm birth severity; (*B*) Infant Sex; (*C*) Maternal Age; (*D*) Paternal Age; (*E*) Region; (*F*) Parity.

**Table S5**: Attributable fractions (AF) and numbers (AN) with 95% confidence intervals (CI) associated with heat, cold, and total non-optimal temperatures, stratified by subperiod.

**Figure S4:** Prefecture-specific heat- and cold-attributable fractions in 1979–1989 and 2012-2023.

**Figure S5:** *(A)* Lag-cumulative relative risk (RR) curves for mean temperature and preterm birth stratified by time periods (1979–2000, 2001–2023). *(B)* Prefecture-specific minimum morbidity temperature (MMT) in 1979–2000 and 2001–2023. *(C)* Prefecture-specific heat-related RR in 1979–2000 and 2001–2023. *(D)* Prefecture-specific cold-related RR in 1979–2000 and 2001–2023.

**Table S6**: Minimum morbidity temperature percentile (MMTP), minimum morbidity temperature (MMT), cold- and heat-related relative risks (RR) with 95% confidence intervals (CI) stratified by two subperiods (1979–2000, 2001–2023).

**Table S7:** Associations between prefectural meta-predictors and minimum morbidity temperature (MMT), and cold- and heat-related relative risks (RR) for the two subperiod model (1979–2000, 2001–2023).

**Figure S6:** Prefecture specific lag-cumulative relative risk (RR) curves for mean temperature and preterm birth stratified by time periods (1979–2023 [entire study period], 1979–1989, 1990–2000, 2001–2011, 2012–2023) using best linear unbiased predictions (BLUPs).

**Figure S7:** Prefecture specific lag-cumulative relative risk (RR) curves for mean temperature and preterm birth stratified by time periods (1979–2023 [entire study period], 1979–1989, 1990–2000, 2001–2011, 2012–2023) without best linear unbiased predictions (BLUPs).

**Figure S1:** Live birth data inclusion criteria flow chart.

Note: Birth data were cleaned to remove births missing birth dates, missing or with abnormal gestational ages <22 week and >44 weeks, location of birth outside of Japan, and abnormally low weights <500g. Gestational age was coded in months (instead of weeks) from 1972–1978, so the analytic sample was limited to births from 1979–2023.

**Table S1**: List of prefectural meta-predictors.

| **Meta-predictor** | **Source** | **Available period** | **Notes** |
| --- | --- | --- | --- |
| Meteorological | Japan Meterological Agency | 1979–2023 | Data were obtained from weather stations at the prefectural capital with the exception of Saitama and Shiga, where no appropriate weather station was located at the prefectural capital. Nearby weather stations in Kumagaya and Hikone were utilized. |
| Mean temperature (°C) |  |  |  |
| Maximum temperature (°C) |  |  |  |
| Minimum temperature (°C) |  |  |  |
| Temperature range (°C) |  |  |  |
| Relative humidity (%) |  |  |  |
| Demographic | Statistics Bureau of the Ministry of Internal Affairs and Communications of Japan | 1979–2023 |  |
| Population (thousand persons) |  |  |  |
| Births |  |  |  |
| Medical Resources |  |  |  |
| Hospitals (per thousand persons) | Statistics Bureau of the Ministry of Internal Affairs and Communications of Japan | 1979–2021 |  |
| Hospital beds (per thousand persons) |  | 1979–2021 |  |
| Doctors (per thousand persons) |  | 1980–2020 (biennially) |  |
| Nurses (per thousand persons) |  | 1980–2020 (biennially) |  |
| Socioeconomic | Statistics Bureau of the Ministry of Internal Affairs and Communications of Japan |  |  |
| Income (million yen) |  | 1979–2022 |  |
| Savings (million yen) |  | 1979–2022 |  |
| Air conditioning (%) | Regional statistics  database | 1979–2009 | Annual prevalence of air conditioning for  households of two or more |

Notes: Time-varying predictors were averaged by subperiod for meta-regression analyses. For the 2011–2023 subperiod, which did not have air conditioning statistics, values were interpolated from the most recent data (2009).

**Table S2:** Subgroup summary statistics for documented live births in Japan, 1979-2023.

| **Measure** | **Overall (1979–2023)** | **1979–1989** | **1990–2000** | **2001–2011** | **2012–2023** |
| --- | --- | --- | --- | --- | --- |
| Total live births | 52,749,360 (100%) | 16,088,250 (100%) | 13,354,755 (100%) | 12,200,706 (100%) | 11,105,649 (100%) |
| *Subgroup composition among all live births* |  |  |  |  |  |
| Infant sex |  |  |  |  |  |
| Male | 27,082,979 (51·34%) | 8,269,179 (51·40%) | 6,858,170 (51·35%) | 6,261,663 (51·32%) | 5,693,967 (51·27%) |
| Female | 25,666,381 (48·66%) | 7,819,071 (48·60%) | 6,496,585 (48·65%) | 5,939,043 (48·68%) | 5,411,682 (48·73%) |
| Maternal age |  |  |  |  |  |
| 35+ | 7,628,014 (14·46%) | 986,218 (6·13%) | 1,300,081 (9·73%) | 2,198,700 (18·02%) | 3,143,015 (28·30%) |
| 25 to 34 | 37,128,044 (70·39%) | 12,060,590 (74·97%) | 9,768,423 (73·15%) | 8,363,447 (68·55%) | 6,935,584 (62·45%) |
| 15 to 24 | 7,991,418 (15·15%) | 3,041,142 (18·90%) | 2,285,736 (17·12%) | 1,637,969 (13·43%) | 1,026,571 (9·24%) |
| Paternal age |  |  |  |  |  |
| 35+ | 14,414,246 (27·33%) | 3,048,156 (18·95%) | 3,367,881 (25·22%) | 3,646,561 (29·89%) | 4,351,648 (39·18%) |
| 25 to 34 | 33,368,501 (63·26%) | 11,702,602 (72·74%) | 8,526,658 (63·85%) | 7,274,780 (59·63%) | 5,864,461 (52·81%) |
| 15 to 24 | 4,070,885 (7·72%) | 1,184,110 (7·36%) | 1,267,808 (9·49%) | 1,004,779 (8·24%) | 614,188 (5·53%) |
| Regions |  |  |  |  |  |
| Northern Japan | 31,392,864 (59·51%) | 9,445,921 (58·71%) | 7,950,622 (59·53%) | 7,323,948 (60·03%) | 6,672,373 (60·08%) |
| Southern Japan | 21,356,496 (40·49%) | 6,642,329 (41·29%) | 5,404,133 (40·47%) | 4,876,758 (39·97%) | 4,433,276 (39·92%) |
| Parity |  |  |  |  |  |
| Primiparous | 24,193,755 (45·87%) | 6,827,778 (42·44%) | 6,304,084 (47·20%) | 5,875,407 (48·16%) | 5,186,486 (46·70%) |
| Multiparous | 28,555,605 (54·13%) | 9,260,472 (57·56%) | 7,050,671 (52·80%) | 6,325,299 (51·84%) | 5,919,163 (53·30%) |

Note: Birth data were cleaned to remove births missing birth dates, missing or with abnormal gestational ages <22 week and >44 weeks, location of birth outside of Japan, and abnormally low weights <500g. Preterm birth severity is divided into moderate to late preterm (32–36 gestational weeks), very preterm (28–31 gestational weeks), and extremely preterm (<28 gestational weeks) per WHO definitions. Northern Japan consists of Japanese Industrial Standard JIS X 0401 prefecture codes 01-23, and Southern Japan 24-47.


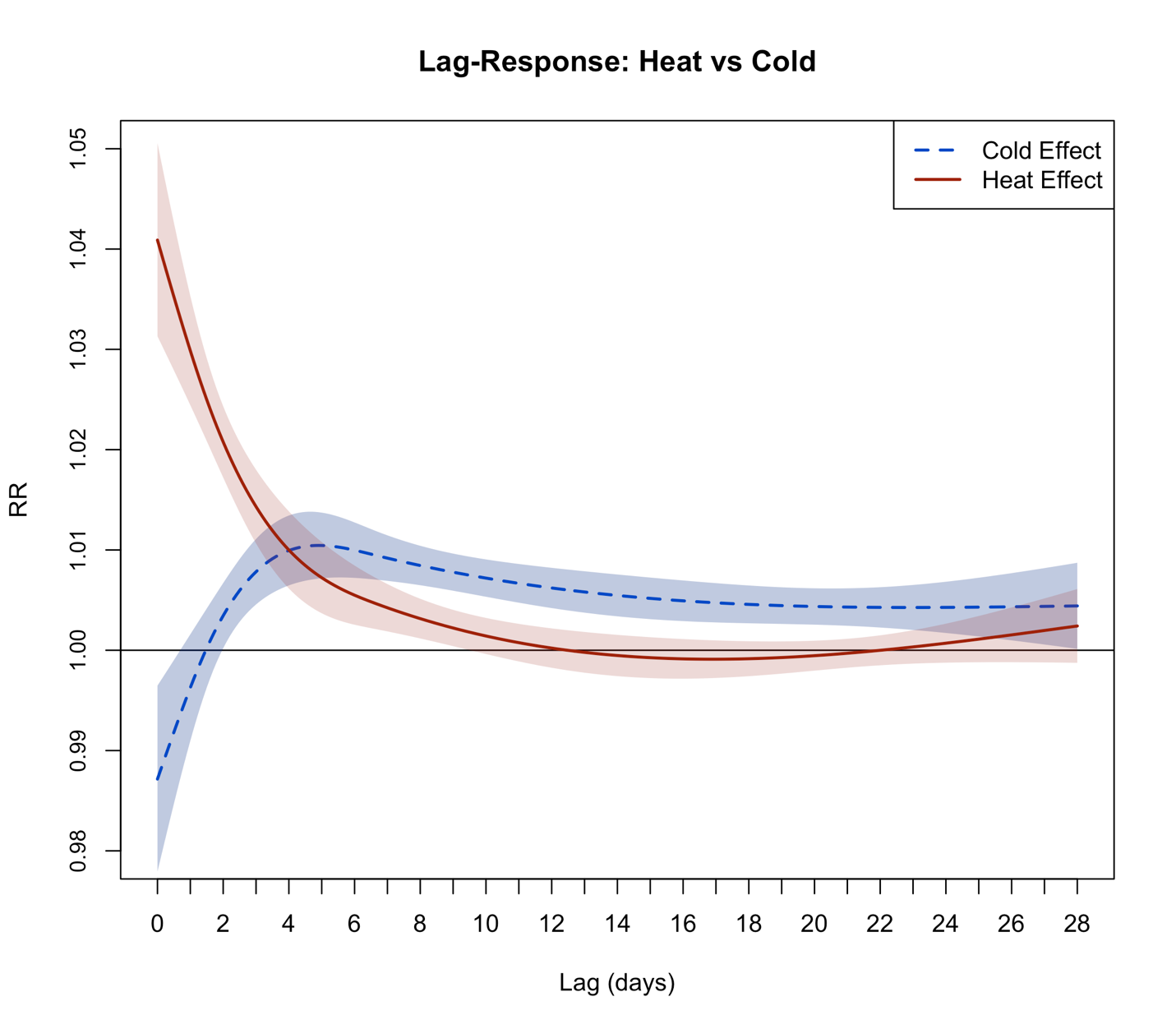


**Figure S2:** The lag-response curve for the hot- and cold-related relative risks (RR) of the temperature-preterm birth association.

**Table S3:** Minimum morbidity temperature percentile (MMTP), minimum morbidity temperature (MMT), cold- and heat-related relative risks (RR) with 95% confidence intervals (CI) stratified by subperiods for the sensitivity analysis model adjusting for relative humidity.

| **Subgroup** | ***I^2^*** | **MMT** | | **Cold-related** | | **Heat-related** | |
| --- | --- | --- | --- | --- | --- | --- | --- |
|  |  | **Percentile (95% CI)** | **MMT (°C) (95% CI)** | **RR (95% CI)** | ***p*-value for difference** | **RR (95% CI)** | ***p*-value for difference** |
| Main model  (1979-2023) | 23·2% | 56th (50th–60th) | 17·70 (16·00–18·70) | 1·11 (1·09–1·14) | - | 1·13 (1·10–1·15) | - |
| *Subperiods* |  |  |  |  |  |  |  |
| 1979-1989 | 9·6% | 59th (54th–63rd) | 17·80 (16·30–18·80) | 1·10 (1·06–1·16) | Reference | 1·17 (1·12–1·22) | Reference |
| 1990-2000 | 6·0% | 49th (37th–59th) | 15·60 (11·70–18·20) | 1·10 (1·05–1·15) | 0·40 | 1·13 (1·09–1·18) | 0·20 |
| 2001-2011 | 0·6% | 54th (39th–65th) | 17·60 (12·40–20·20) | 1·10 (1·05–1·15) | 0·40 | 1·07 (1·03–1·12) | **0·0055*** |
| 2012-2023 | 0·0% | 52nd (40th–62nd) | 17·40 (13·20–19·90) | 1·13 (1·09–1·18) | 0·27 | 1·09 (1·04–1·14) | **0·034*** |

**Table S4:** Main model specification parameter search with quasi-Akaike information criterion (QAIC).

| **Basis function** | **Spline degree** | **Knots used** | **Lag days** | **Lag knots** | **Total QAIC** | **ΔQAIC** |
| --- | --- | --- | --- | --- | --- | --- |
| ns | 1 | 33%, 66% | 28 | 2 | 2,460,068 | 0 |
| ns | 1 | 50% | 28 | 2 | 2,460,071 | 3 |
| ns | 1 | 33%, 66% | 28 | 3 | 2,460,207 | 139 |
| ns | 1 | 50% | 28 | 3 | 2,460,207 | 139 |
| bs | 2 | 50% | 28 | 2 | 2,460,218 | 149 |
| bs | 2 | 33%, 66% | 28 | 2 | 2,460,226 | 158 |
| ns | 1 | 25%, 50%, 75% | 28 | 2 | 2,460,226 | 158 |
| bs | 2 | 25%, 50%, 75% | 28 | 2 | 2,460,342 | 274 |
| ns | 1 | 10%, 75%, 90% | 28 | 2 | 2,460,349 | 281 |
| bs | 2 | 50% | 28 | 3 | 2,460,376 | 308 |
| ns | 1 | 25%, 50%, 75% | 28 | 3 | 2,460,385 | 317 |
| bs | 2 | 33%, 66% | 28 | 3 | 2,460,386 | 318 |
| bs | 2 | 10%, 75%, 90% | 28 | 2 | 2,460,501 | 433 |
| bs | 2 | 25%, 50%, 75% | 28 | 3 | 2,460,550 | 482 |
| ns | 1 | 10%, 75%, 90% | 28 | 3 | 2,460,562 | 493 |
| bs | 2 | 10%, 75%, 90% | 28 | 3 | 2,460,744 | 676 |

**Figure S3:** Lag-cumulative relative risk (RR) curves for mean temperature and preterm birth stratified by subgroups: (*A*) Preterm birth severity; (*B*) Infant Sex; (*C*) Maternal Age; (*D*) Paternal Age; (*E*) Region; (*F*) Parity.

Note: Preterm birth severity is divided into moderate to late preterm (32–36 gestational weeks), very preterm (28–31 gestational weeks), and extremely preterm (<28 gestational weeks) per WHO definitions. Northern Japan consists of Japanese Industrial Standard JIS X 0401 prefecture codes 01-23, and Southern Japan 24-47.

**Table S5:** Attributable fractions (AF) and numbers (AN) with 95% confidence intervals (CI) associated with heat, cold, and total non-optimal temperatures, stratified by subperiod.

| **Subperiod** | **Total** | | **Cold-related** | | **Heat-related** | |
| --- | --- | --- | --- | --- | --- | --- |
|  | **AF (95% CI)** | **AN (95% CI)** | **AF (95% CI)** | **AN (95% CI)** | **AF (95% CI)** | **AN (95% CI)** |
| Main model (1979–2023) | 4·7% (4·3–5·0%) | 102,266 (93,563–108,794) | 2·8% (2·4–3·1%) | 60,527 (51,880–67,012) | 1·9% (1·7–2·1%) | 41,739 (37,345–46,133) |
| 1979–1989 | 5·5% (4·9–6·1%) | 33,577 (29,914–37,240) | 3·2% (2·8–3·6%) | 19,561 (17,116–22,006) | 2·3% (2·0–2·6%) | 14,016 (12,188–15,844) |
| 1990–2000 | 4·7% (4·1–5·2%) | 26,105 (22,772–28,882) | 2·3% (1·7–2·8%) | 12,765 (9,435–15,540) | 2·4% (2·0–2·8%) | 13,340 (11,117–15,563) |
| 2001–2011 | 4·0% (3·6–4·4%) | 21,680 (19,512–23,848) | 2·7% (2·0–3·2%) | 14,357 (10,635–17,016) | 1·4% (0·9–1·8%) | 7,323 (4,708–9,415) |
| 2012–2023 | 4·7% (4·4–4·9%) | 23,343 (21,853–24,336) | 3·2% (2·9–3·4%) | 15,769 (14,291–16,755) | 1·5% (1·3–1·7%) | 7,574 (6,564–8,584) |

**Figure S4:** Prefecture-specific heat- and cold-attributable fractions in 1979–1989 and 2012-2023.

**Figure S5:** *(A)* Lag-cumulative relative risk (RR) curves for mean temperature and preterm birth stratified by time periods (1979–2000, 2001–2023). *(B)* Prefecture-specific minimum morbidity temperature (MMT) in 1979–2000 and 2001–2023. *(C)* Prefecture-specific heat-related RR in 1979–2000 and 2001–2023. *(D)* Prefecture-specific cold-related RR in 1979–2000 and 2001–2023.

**Table S6:** Minimum morbidity temperature percentile (MMTP), minimum morbidity temperature (MMT), cold- and heat-related relative risks (RR) with 95% confidence intervals (CI) stratified by two subperiods (1979–2000, 2001–2023).

| **Subgroup** | ***I^2^*** | **MMT** | | **Cold-related** | | **Heat-related** | |
| --- | --- | --- | --- | --- | --- | --- | --- |
|  |  | **Percentile (95% CI)** | **MMT (°C) (95% CI)** | **RR (95% CI)** | ***p*-value for difference** | **RR (95% CI)** | ***p*-value for difference** |
| Main model (1979-2023) | 24·9% | 56th (51st–60th) | 17·70 (16·20–18·70) | 1·11 (1·09–1·14) | - | 1·13 (1·10–1·15) | - |
| *Subperiods* |  |  |  |  |  |  |  |
| 1979-2000 | 13·8% | 57th (52nd–61st) | 17·50 (16·10–18·50) | 1·10 (1·07–1·14) | Reference | 1·15 (1·11–1·18) | Reference |
| 2001-2023 | 0·0% | 54th (45th–61st) | 17·50 (14·60–19·40) | 1·13 (1·09–1·16) | 0·197 | 1·09 (1·05–1·12) | **0·024*** |

Note: *p*-values for difference were considered significant *p* < 0·05.

**Table S7:** Associations between prefectural meta-predictors and minimum morbidity temperature (MMT), and cold- and heat-related relative risks (RR) with 95% confidence intervals (CI) for the two subperiod model (1979–2000, 2001–2023).

| **Meta-predictor** | **MMT** | | **Cold-related RR** | | **Heat-related RR** | |
| --- | --- | --- | --- | --- | --- | --- |
|  | **Coefficient (95% CI)** | ***p*-value** | **Coefficient (95% CI)** | ***p*-value** | **Coefficient (95% CI)** | ***p*-value** |
| Meteorological |  |  |  |  |  |  |
| Mean temperature | 1·957 (1·762, 2·152) | **<0·0001*** | -0·003 (-0·013, 0·006) | 0·52 | -0·003 (-0·009, 0·002) | 0·22 |
| Maximum temperature | 2·033 (1·823, 2·242) | **<0·0001*** | -0·002 (-0·012, 0·007) | 0·65 | -0·003 (-0·008, 0·003) | 0·30 |
| Minimum temperature | 1·852 (1·666, 2·038) | **<0·0001*** | -0·003 (-0·012, 0·006) | 0·53 | -0·004 (-0·009, 0·001) | 0·15 |
| Temperature range | -1·486 (-1·756, -1·216) | **<0·0001*** | -0·001 (-0·010, 0·008) | 0·80 | 0·003 (-0·002, 0·009) | 0·24 |
| Relative humidity | -0·375 (-0·936, 0·186) | 0·19 | -0·000 (-0·009, 0·009) | 0·98 | 0·001 (-0·004, 0·007) | 0·64 |
| Demographic |  |  |  |  |  |  |
| Population | 0·046 (-0·562, 0·653) | 0·88 | -0·003 (-0·011, 0·006) | 0·51 | 0·000 (-0·005, 0·005) | 0·90 |
| Births | -0·005 (-0·568, 0·557) | 0·99 | -0·007 (-0·015, 0·002) | 0·12 | 0·004 (-0·001, 0·009) | 0·15 |
| Medical resources |  |  |  |  |  |  |
| Hospitals | 0·335 (-0·204, 0·875) | 0·22 | -0·005 (-0·015, 0·004) | 0·26 | 0·003 (-0·003, 0·008) | 0·36 |
| Hospital beds | 0·285 (-0·272, 0·843) | 0·32 | -0·003 (-0·012, 0·006) | 0·52 | -0·001 (-0·006, 0·005) | 0·85 |
| Doctors | 0·232 (-0·133, 0·597) | 0·21 | 0·011 (0·002, 0·021) | 0·016 | -0·016 (-0·022, -0·011) | **<0·0001*** |
| Nurses | 0·120 (-0·203, 0·444) | 0·47 | 0·020 (0·010, 0·029) | **<0·0001*** | -0·024 (-0·030, -0·018) | **<0·0001*** |
| Socioeconomic |  |  |  |  |  |  |
| Income | -0·165 (-0·593, 0·262) | 0·45 | 0·013 (0·004, 0·022) | **0·0061*** | -0·009 (-0·014, -0·004) | **0·00091*** |
| Savings | 0·063 (-0·257, 0·382) | 0·70 | 0·023 (0·014, 0·033) | **<0·0001*** | -0·026 (-0·032, -0·019) | **<0·0001*** |
| Air conditioning | 0·687 (0·324, 1·051) | **<0·0001*** | 0·012 (0·002, 0·022) | 0·016 | -0·016 (-0·022, -0·010) | **<0·0001*** |

Note: Estimates are presented as the change in log-RR or MMT per one standard deviation increase in the predictor, along with 95% CI and *p*-values. Statistical significance was defined as *p* < 0·01 to account for multiple comparisons.


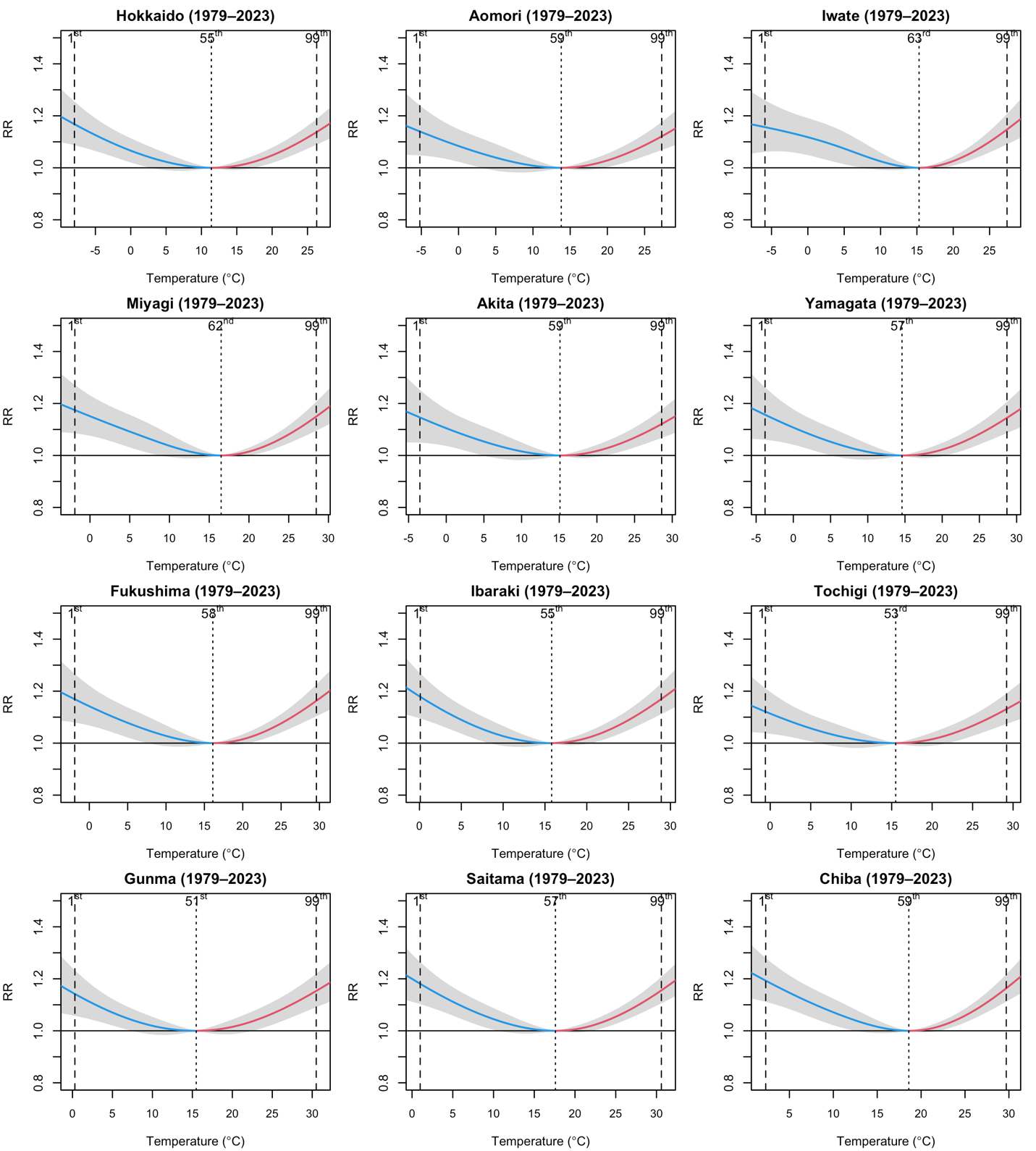


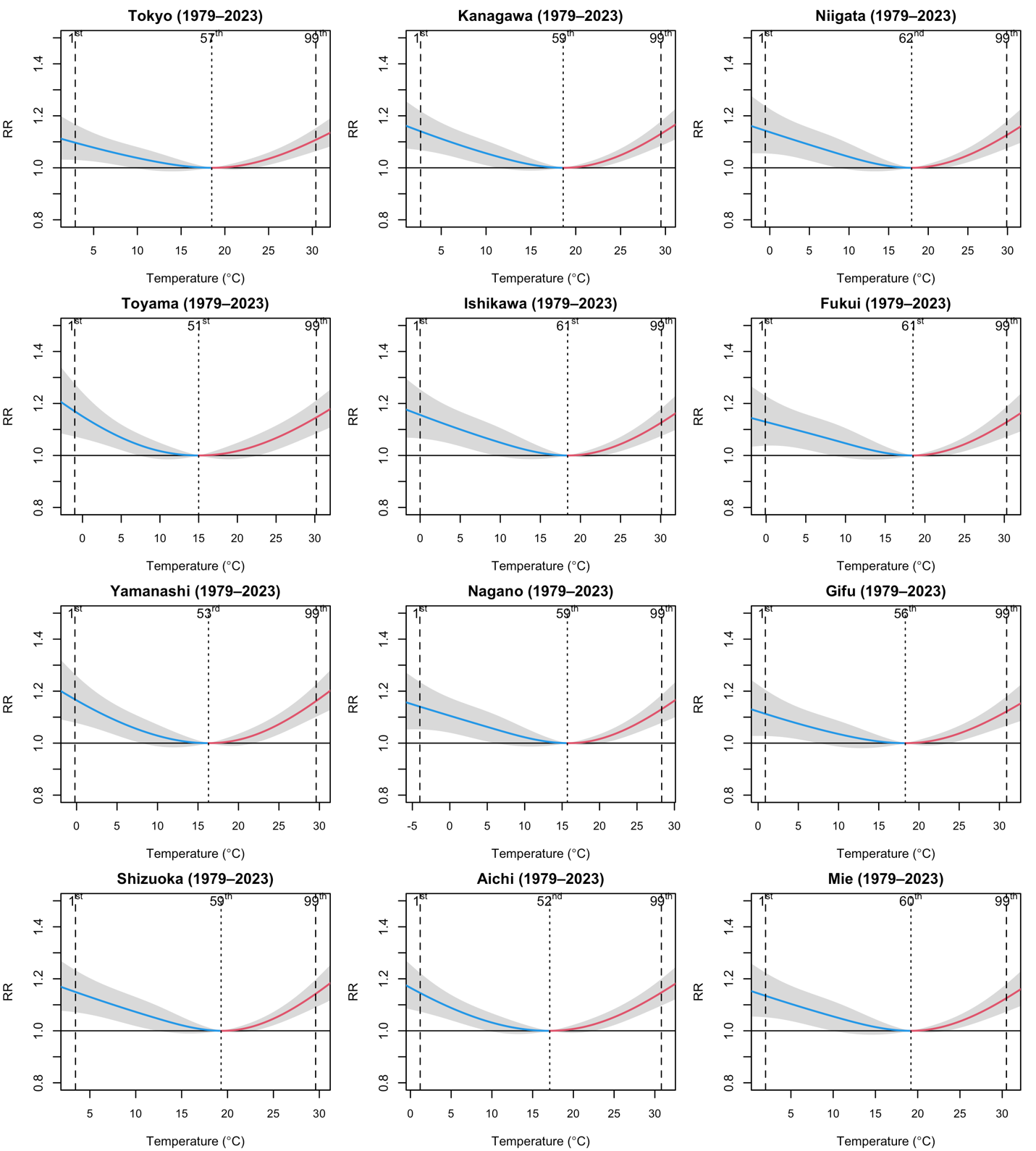


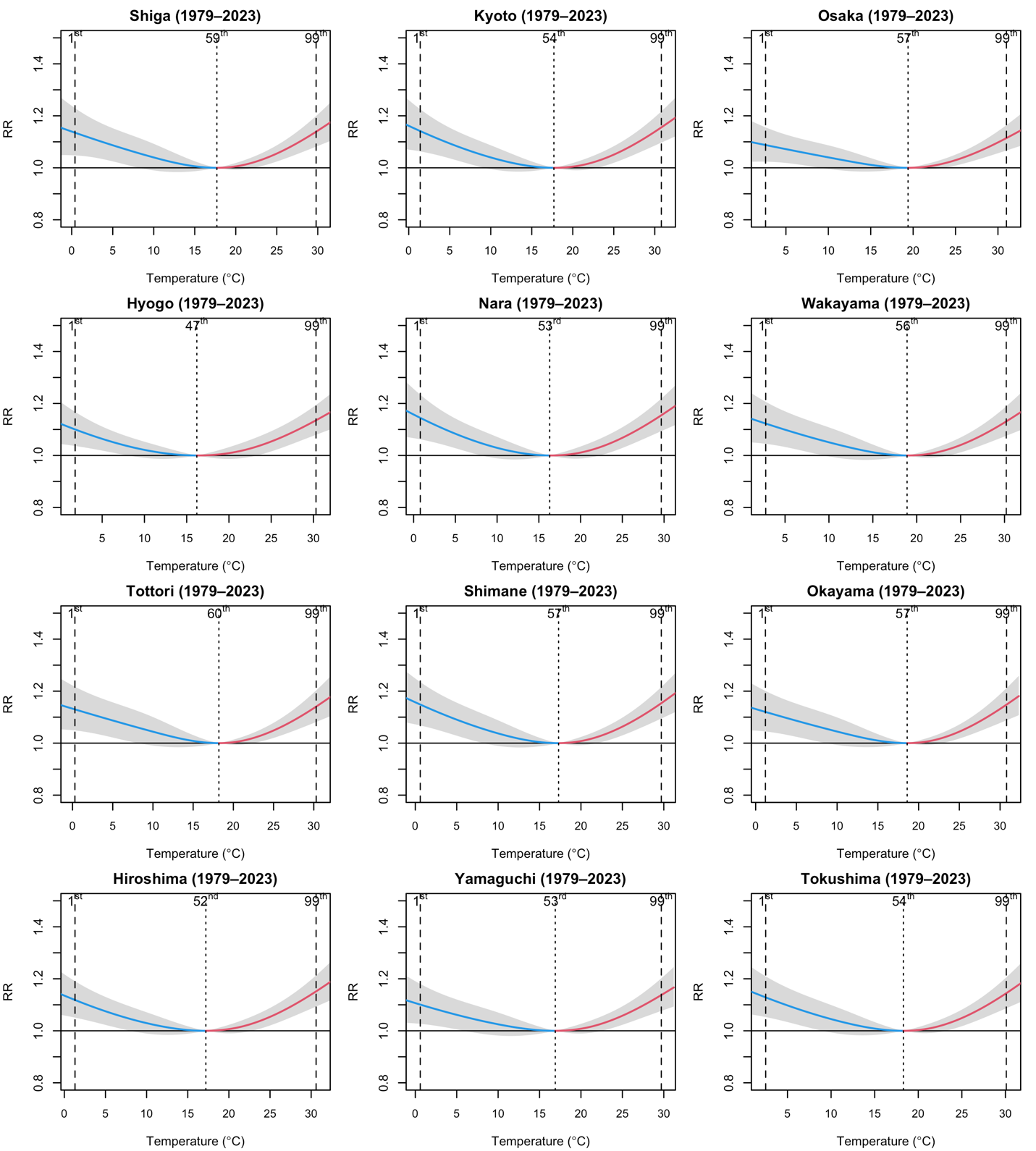


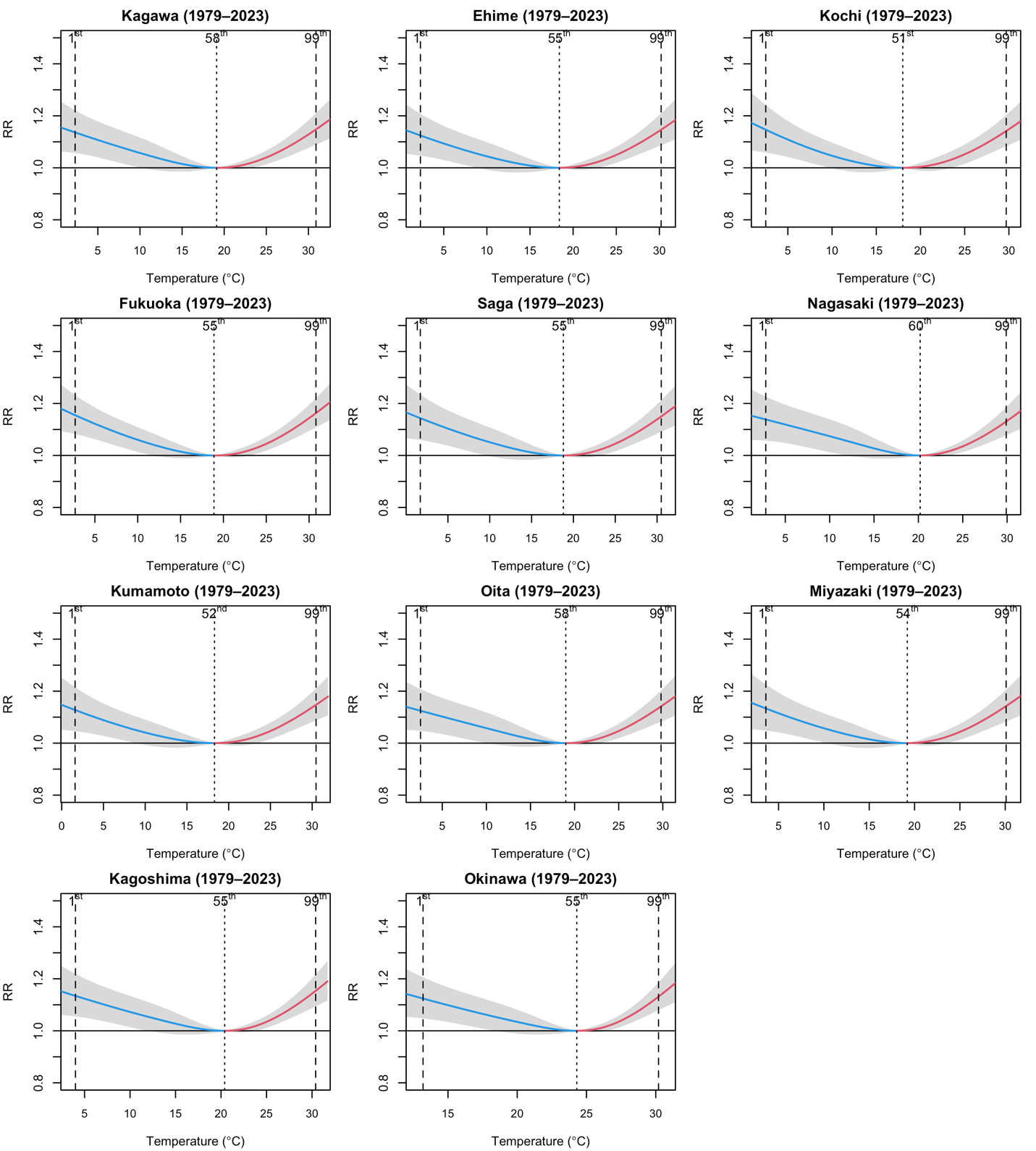


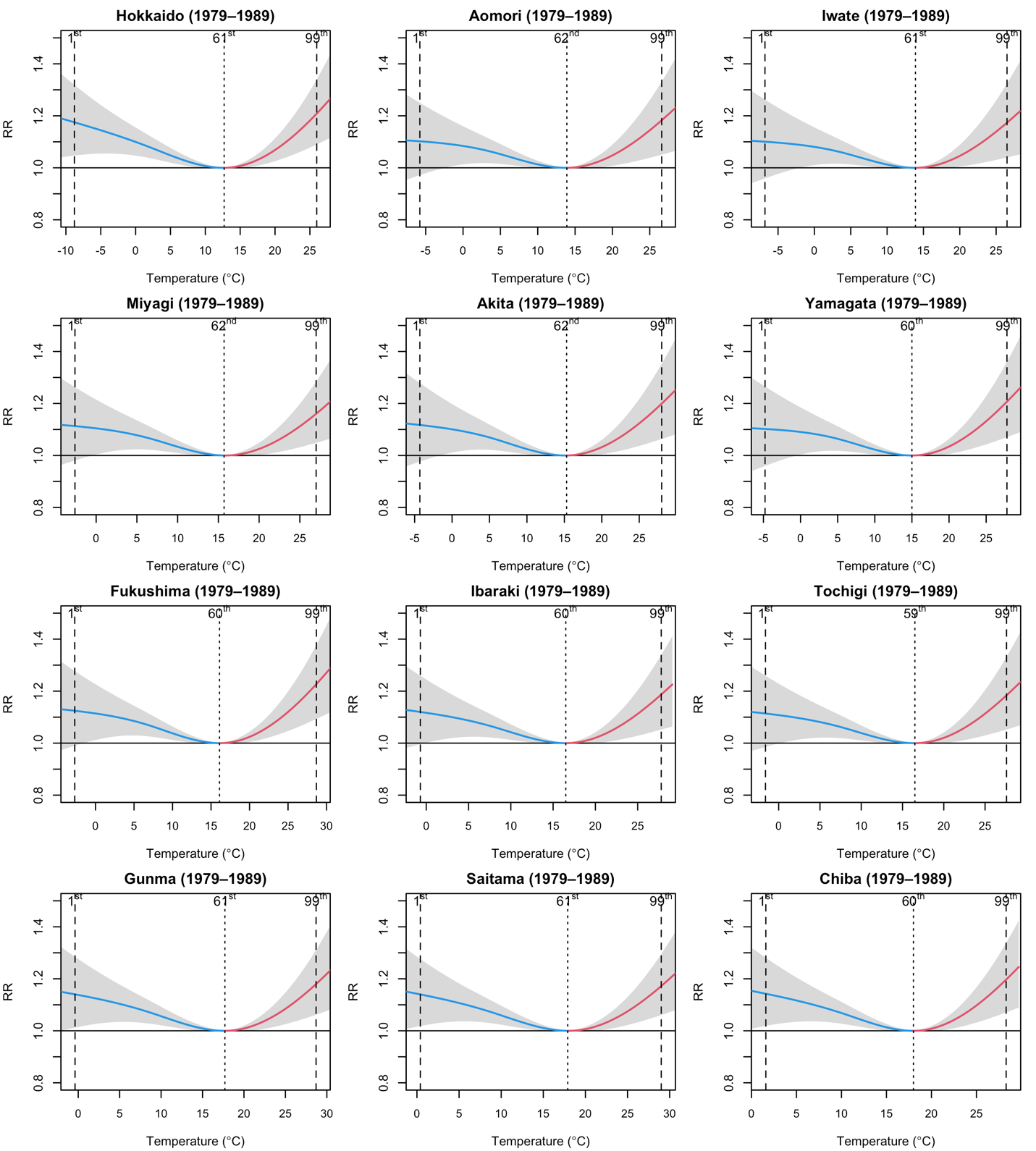


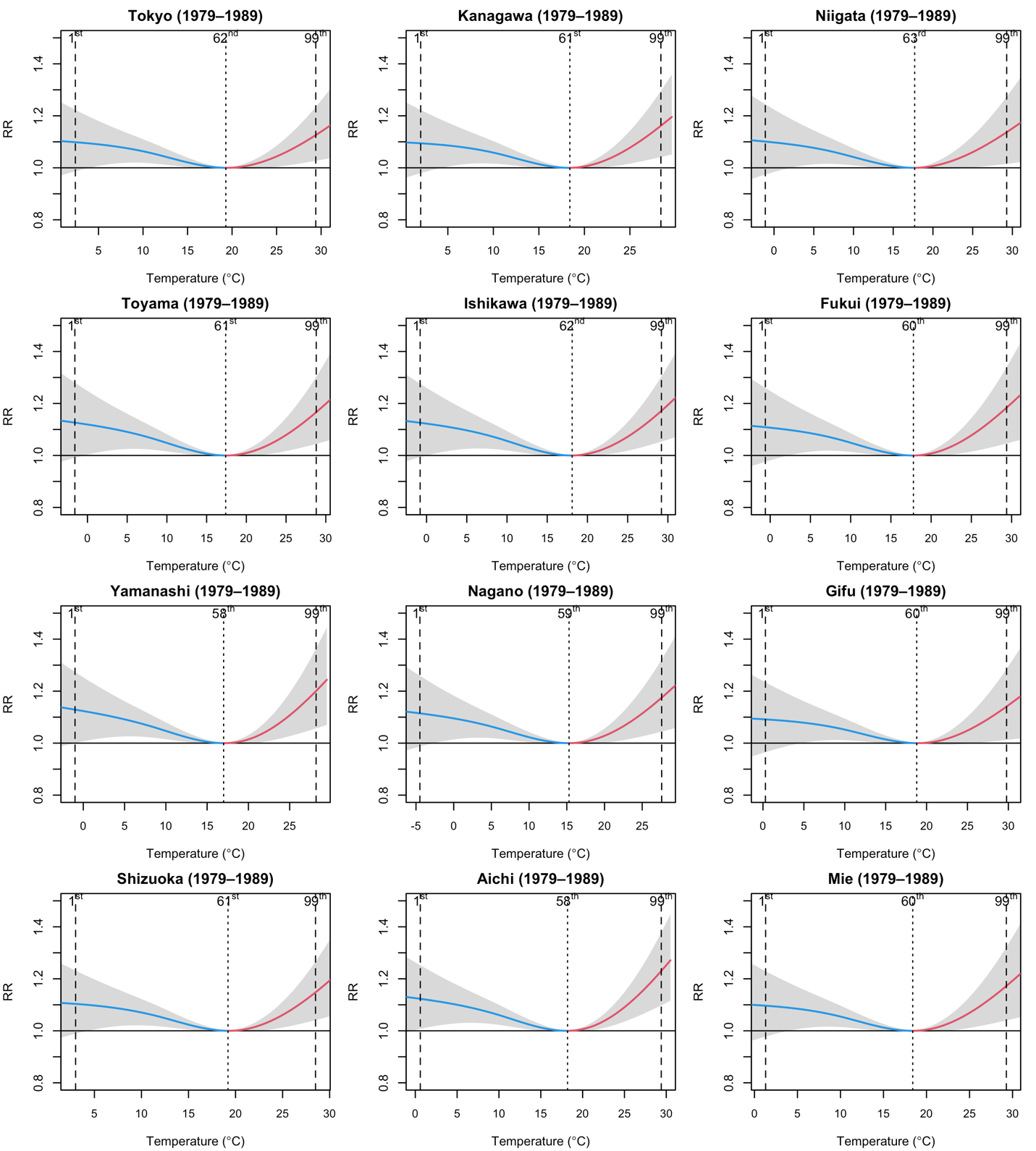


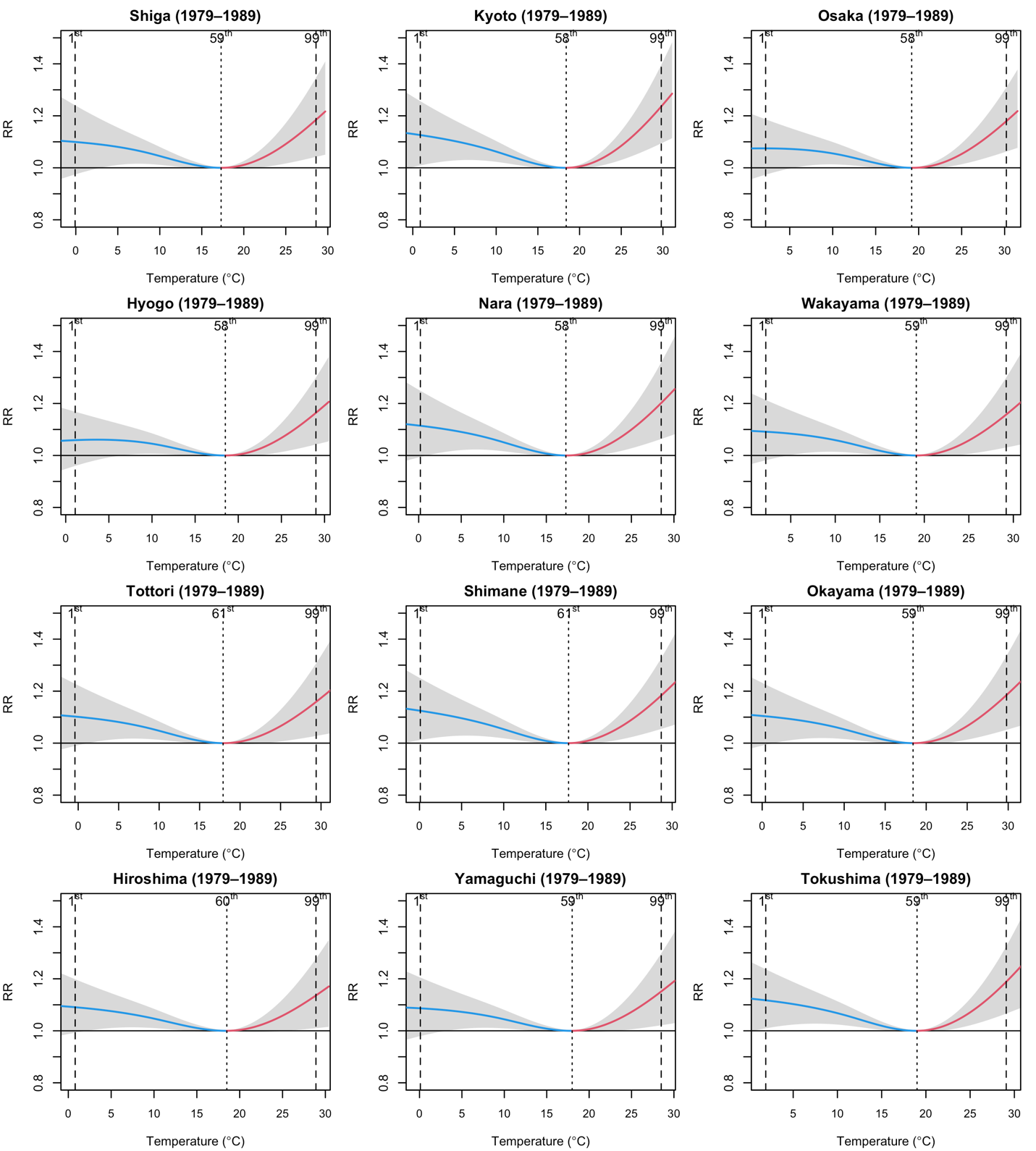


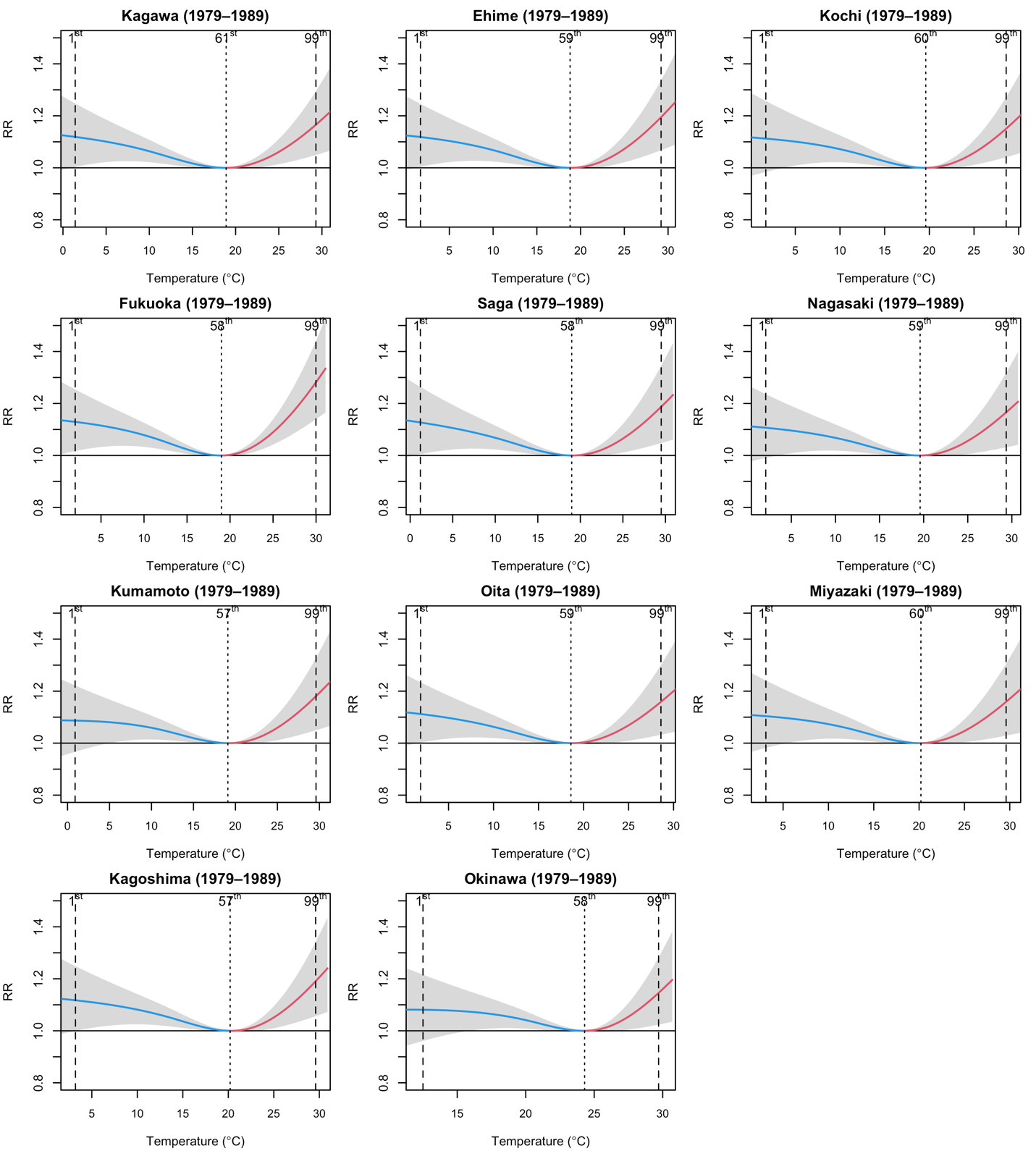


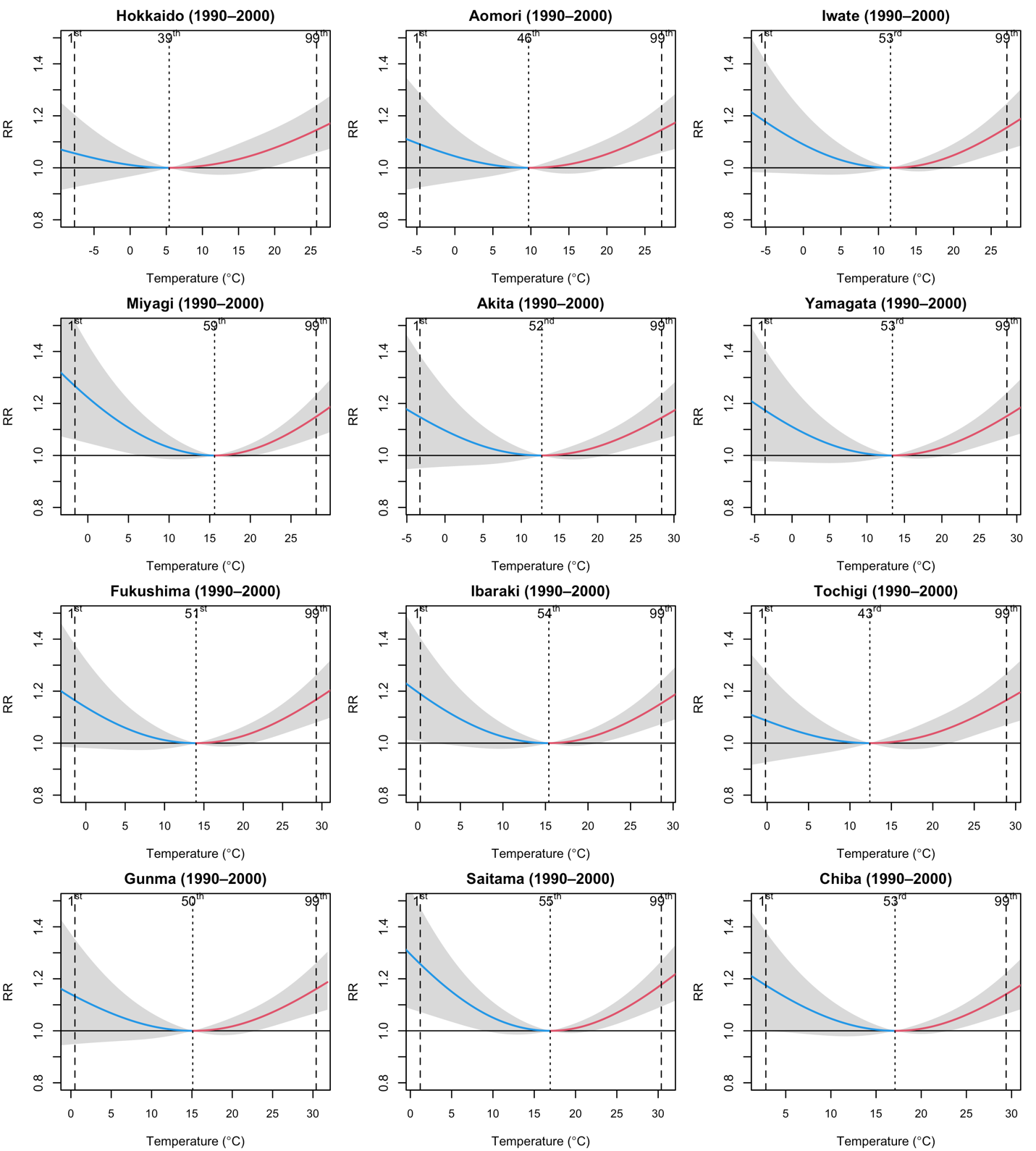


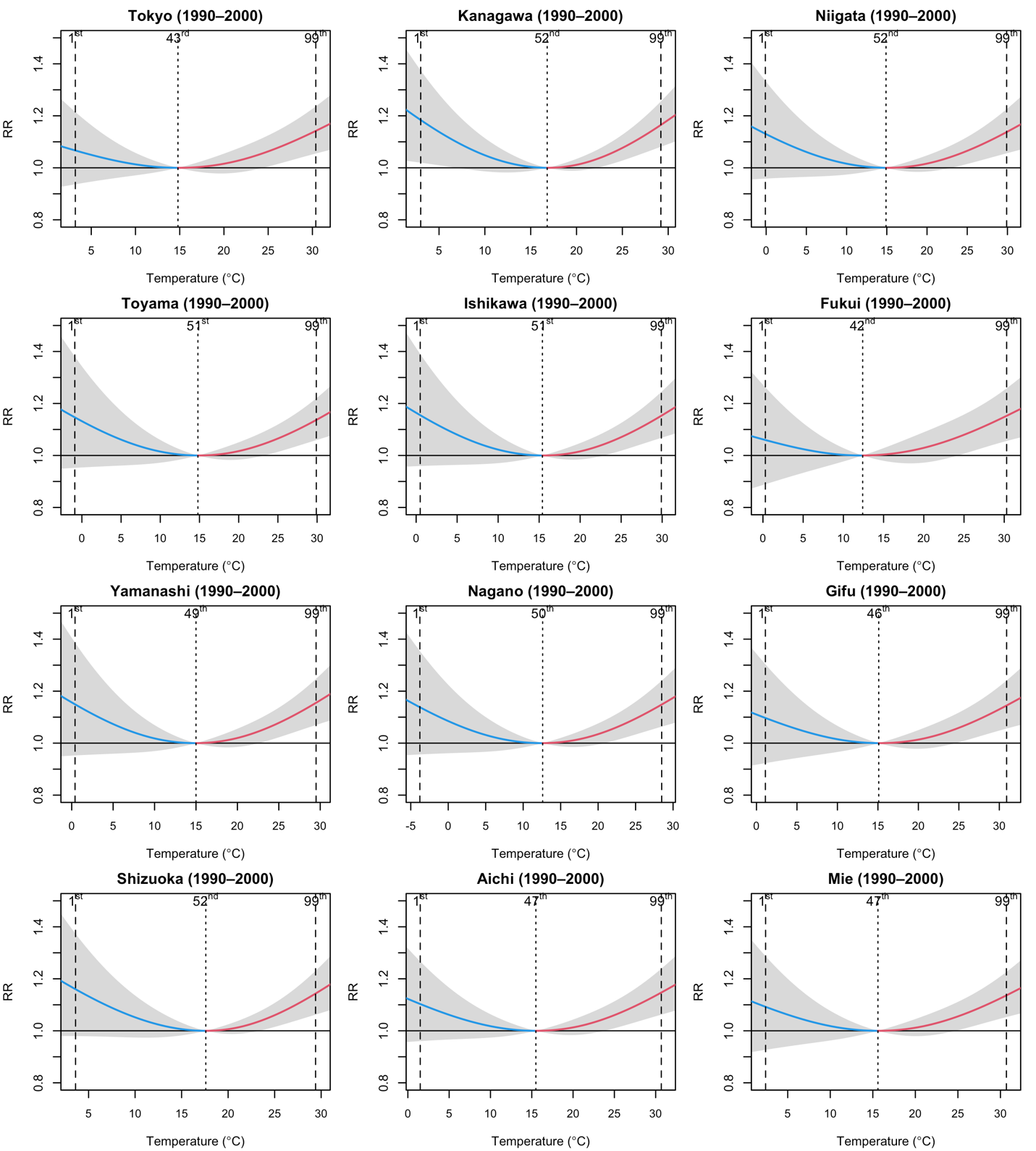


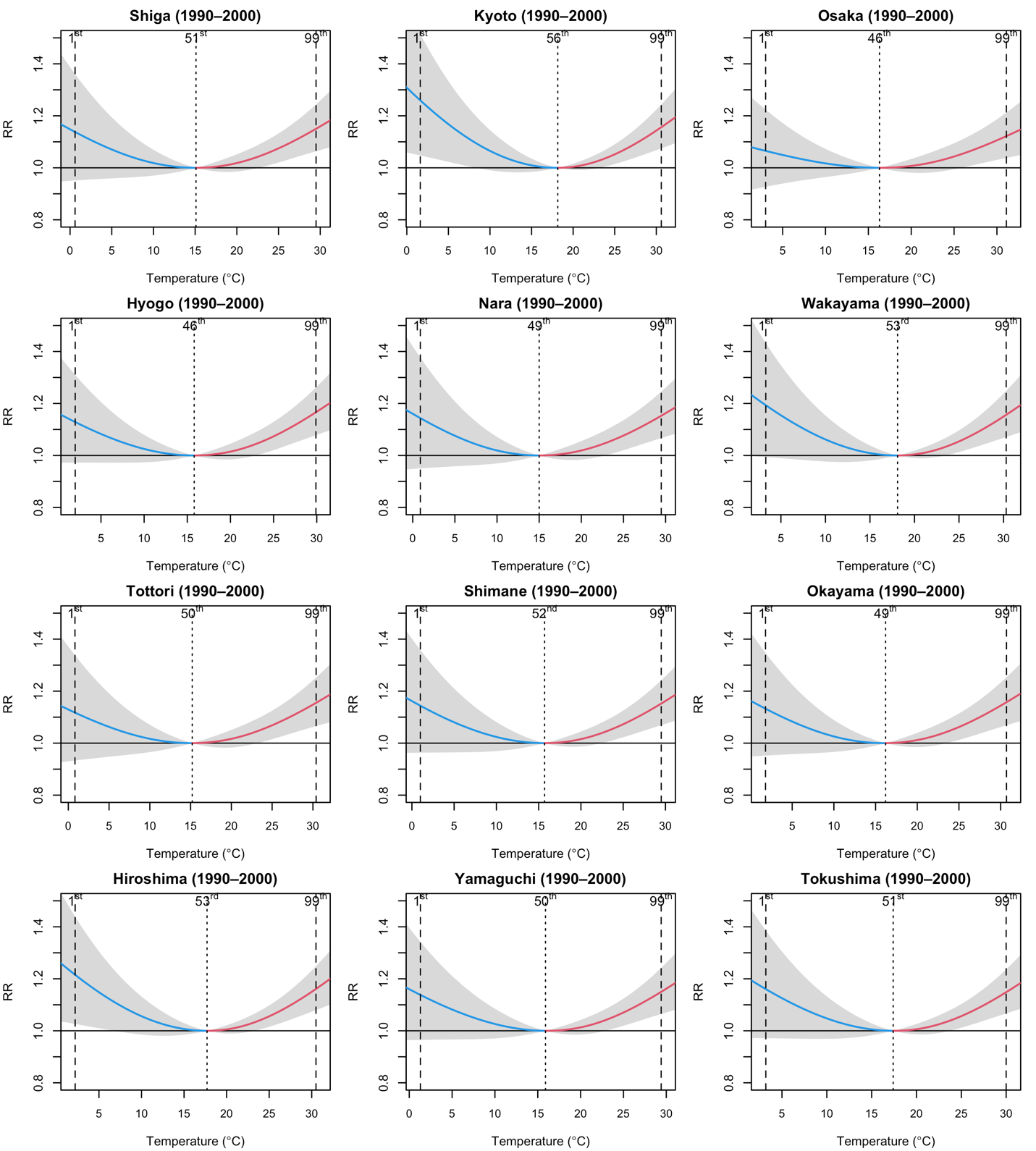


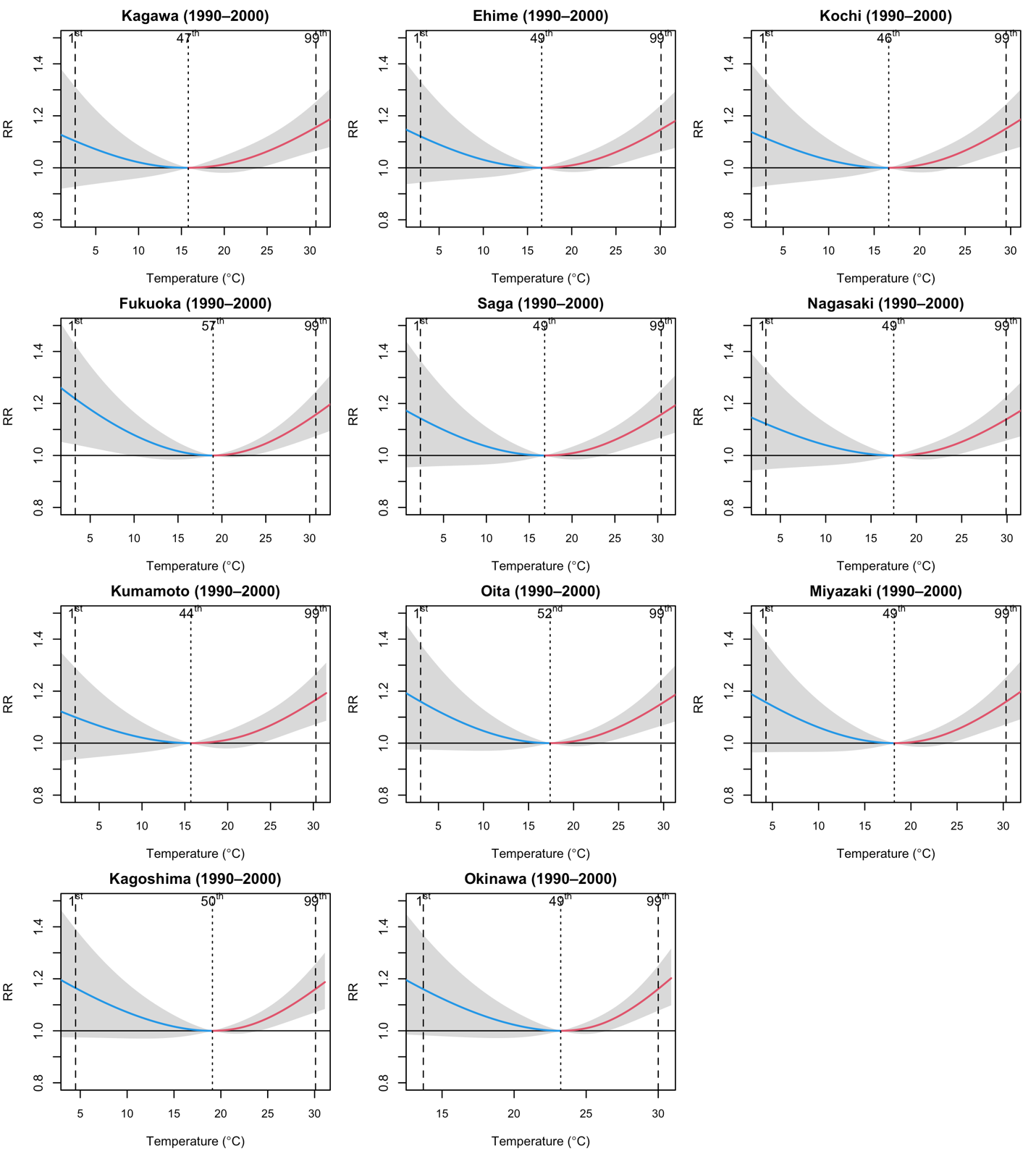


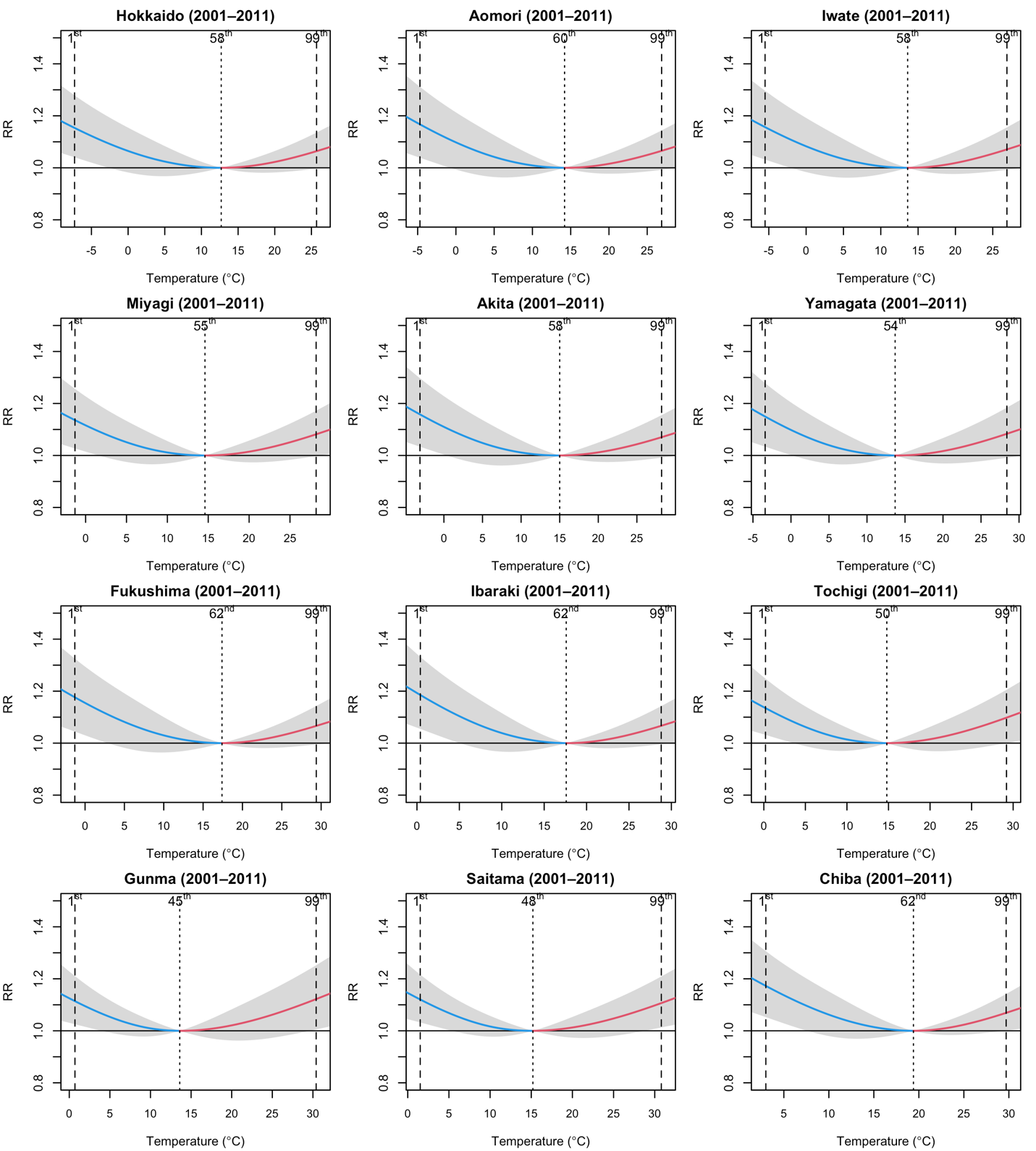


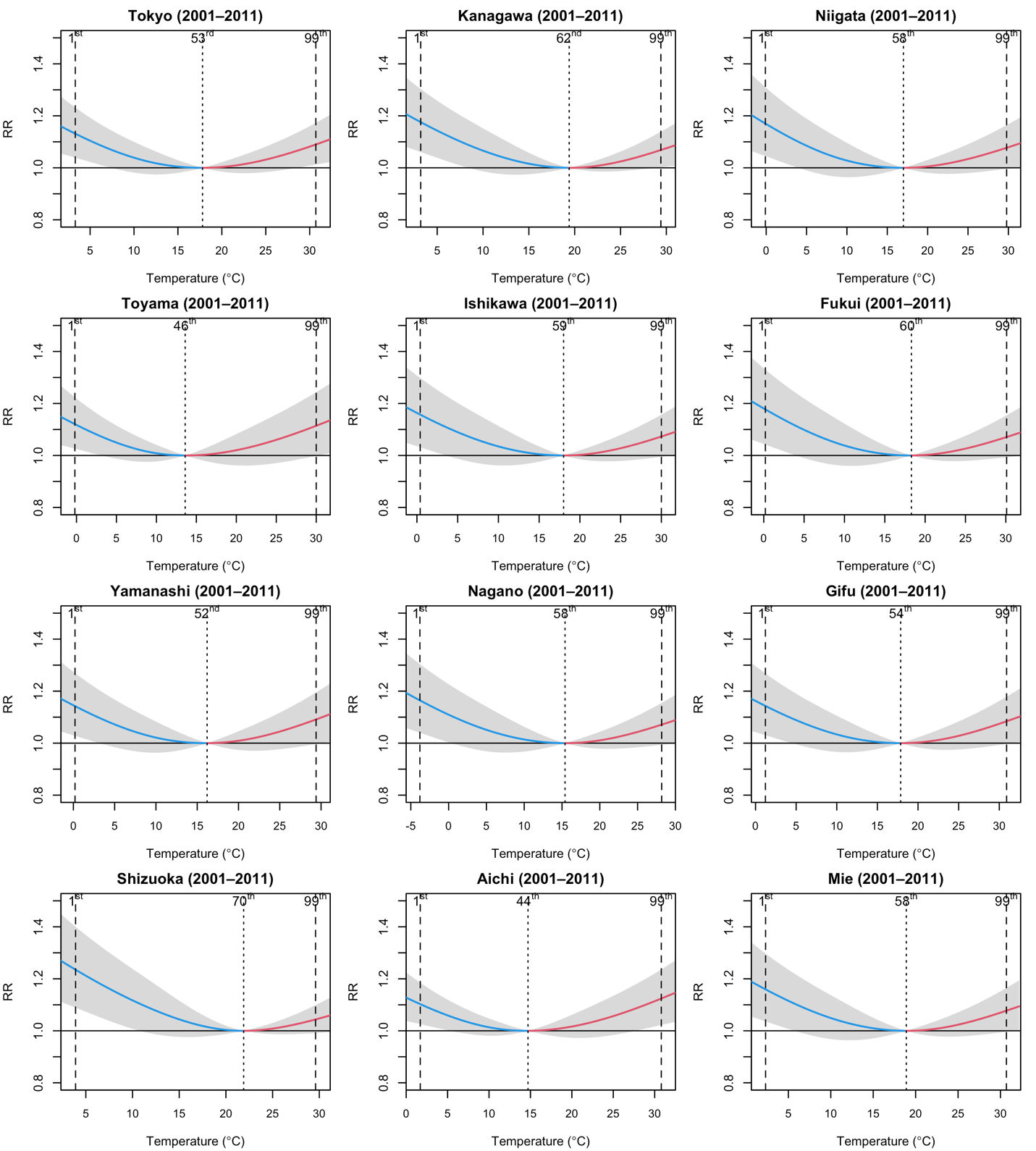


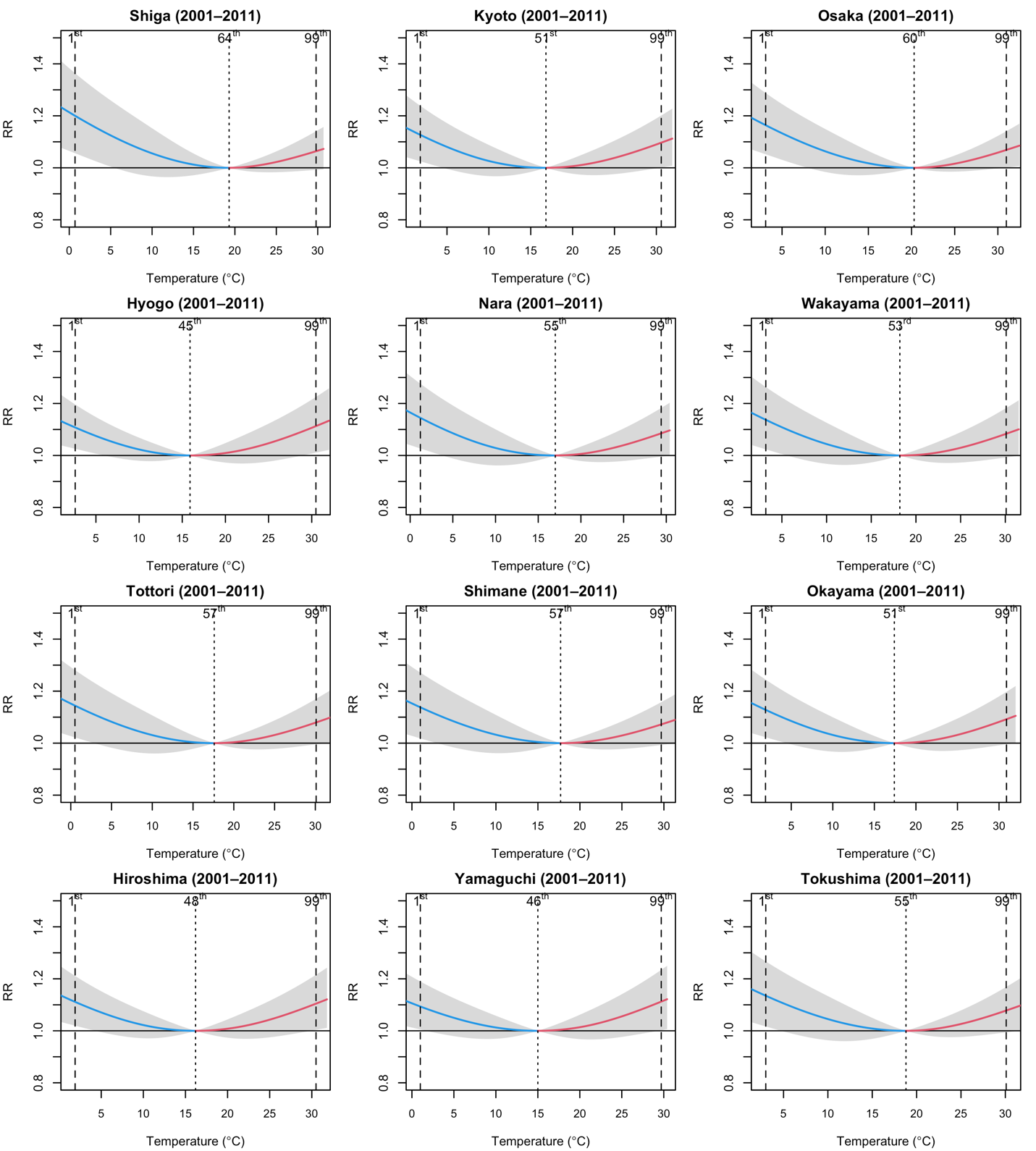


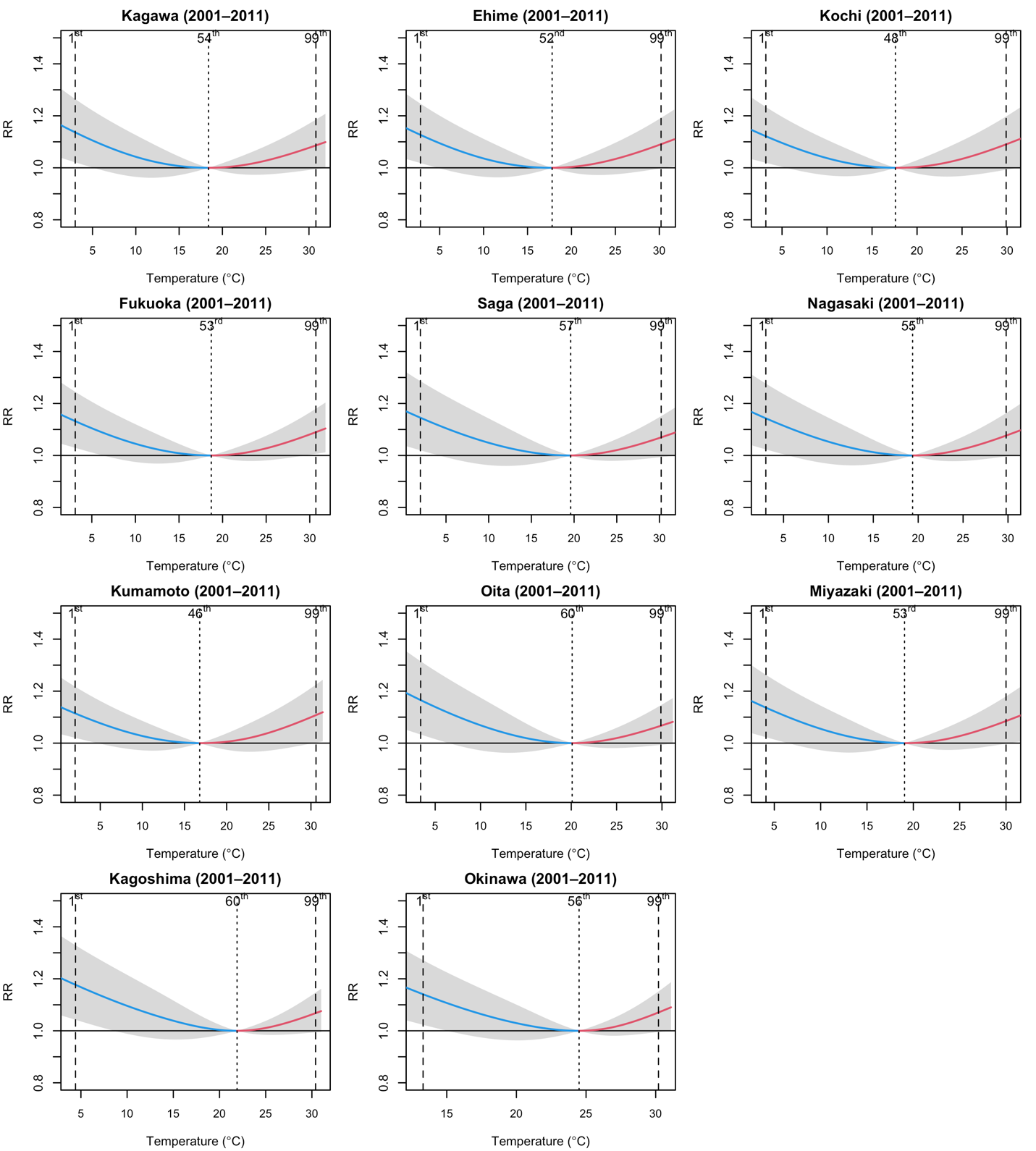


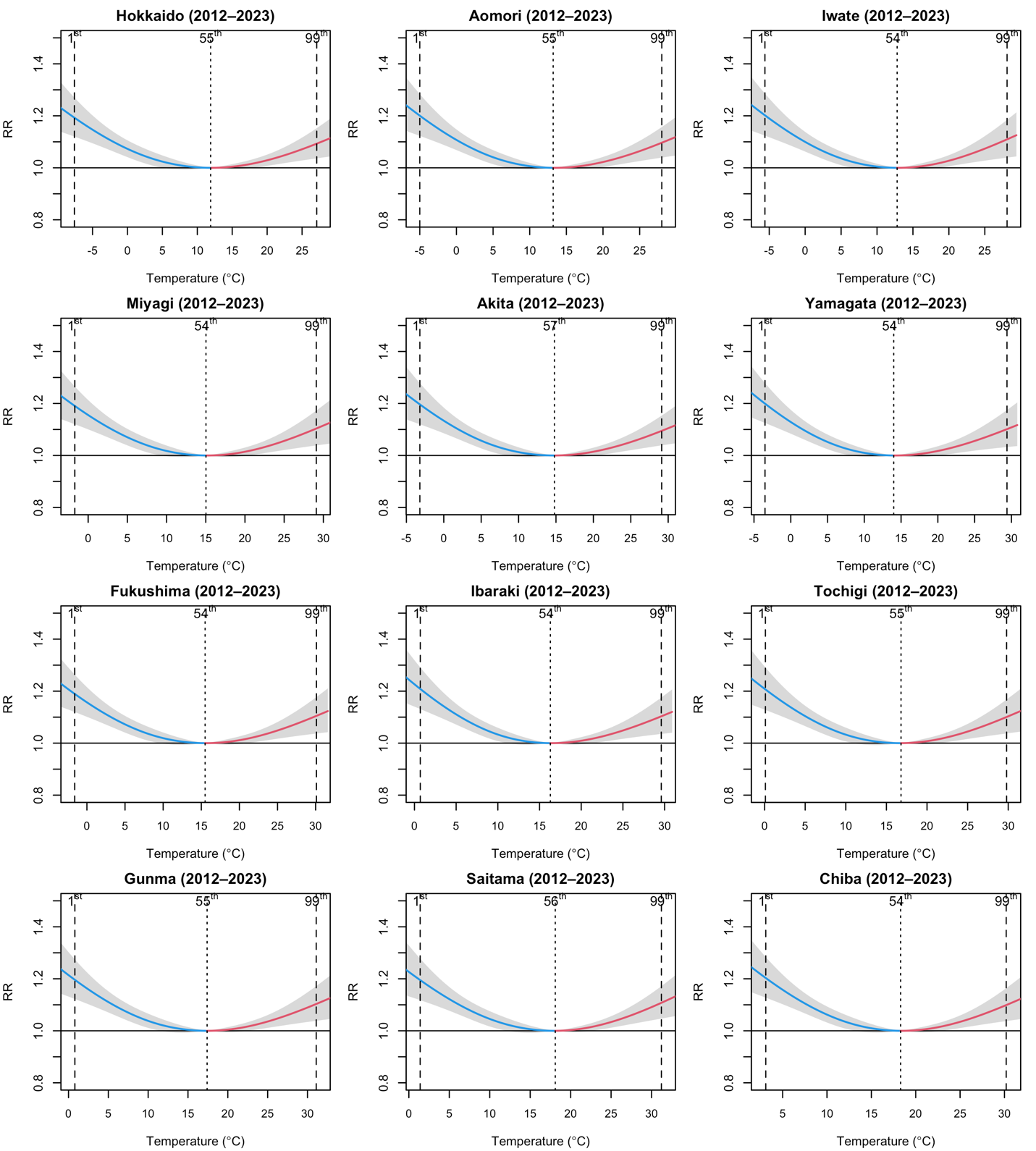


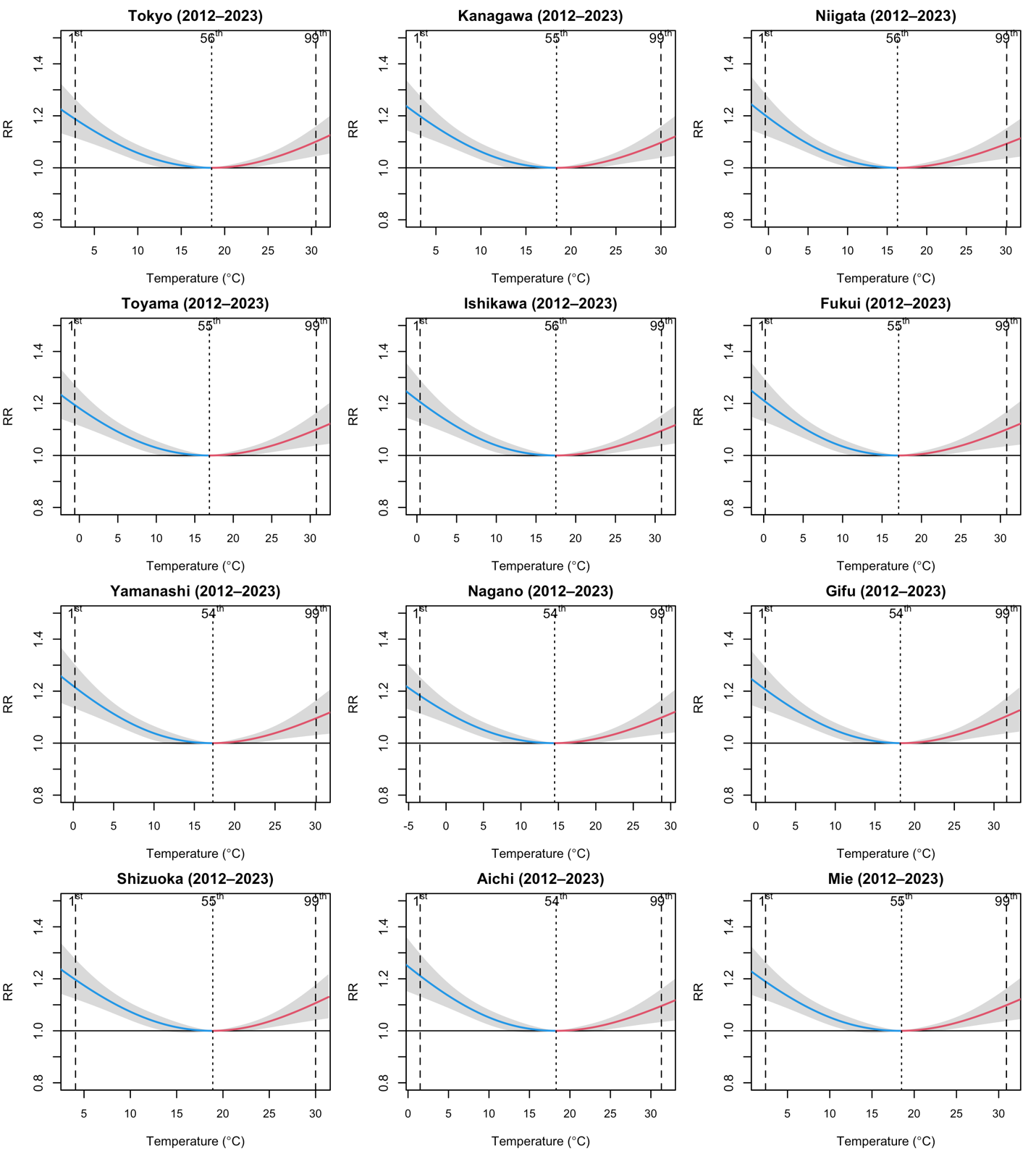


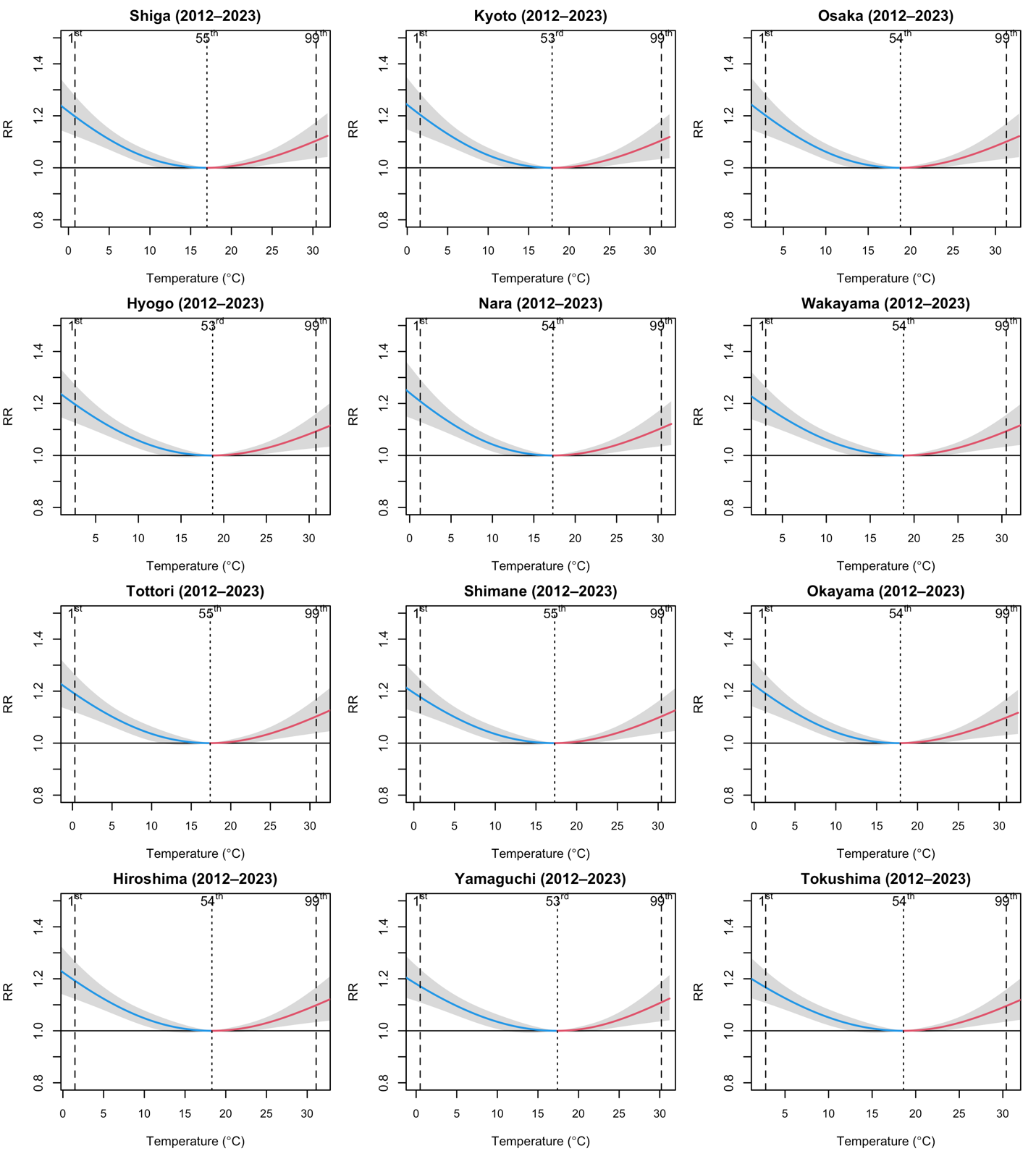


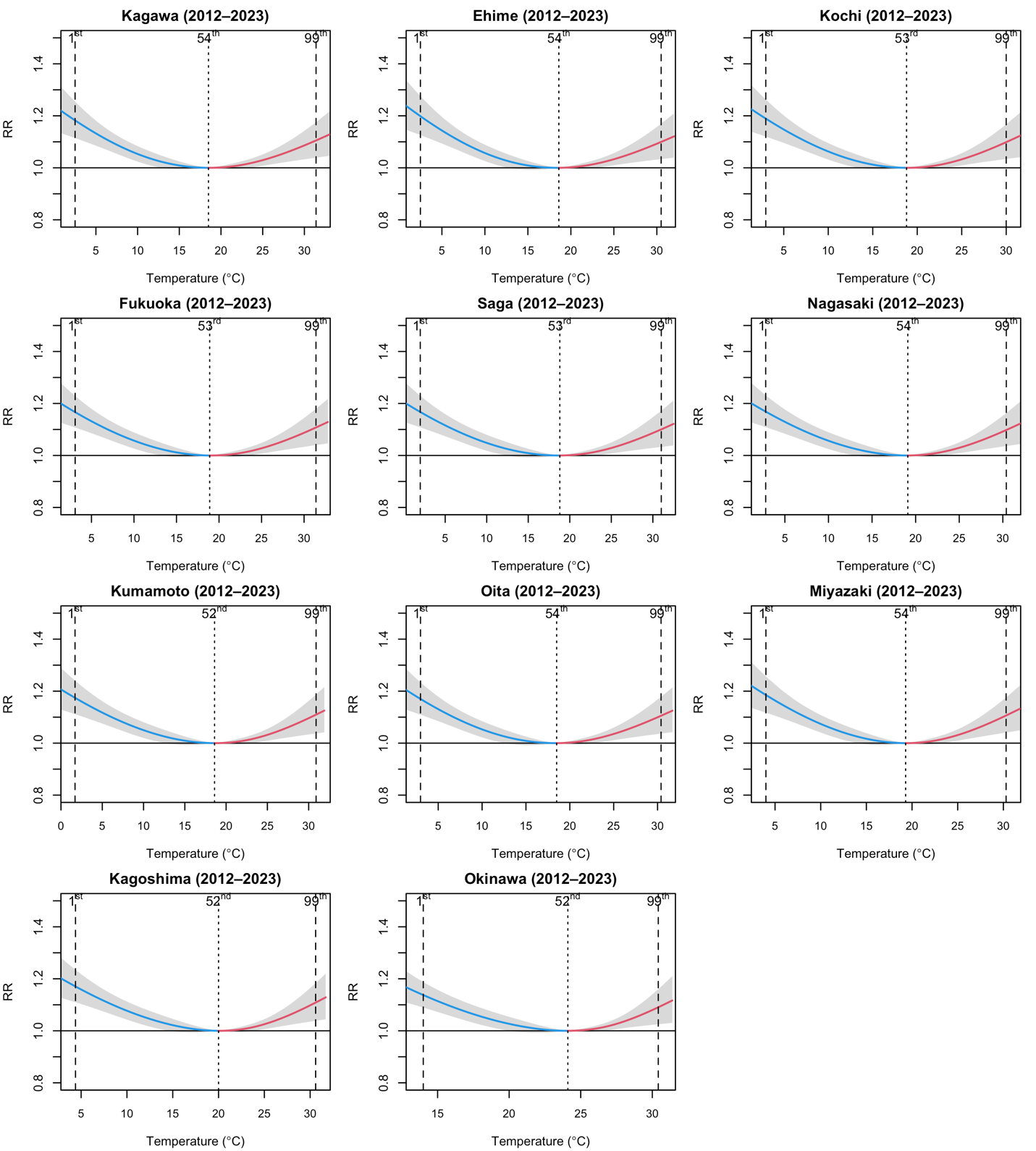


**Figure S6:** Prefecture specific lag-cumulative relative risk (RR) curves for mean temperature and preterm birth stratified by time periods (1979–2023 [entire study period], 1979–1989, 1990–2000, 2001–2011, 2012–2023) using best linear unbiased predictions (BLUPs).


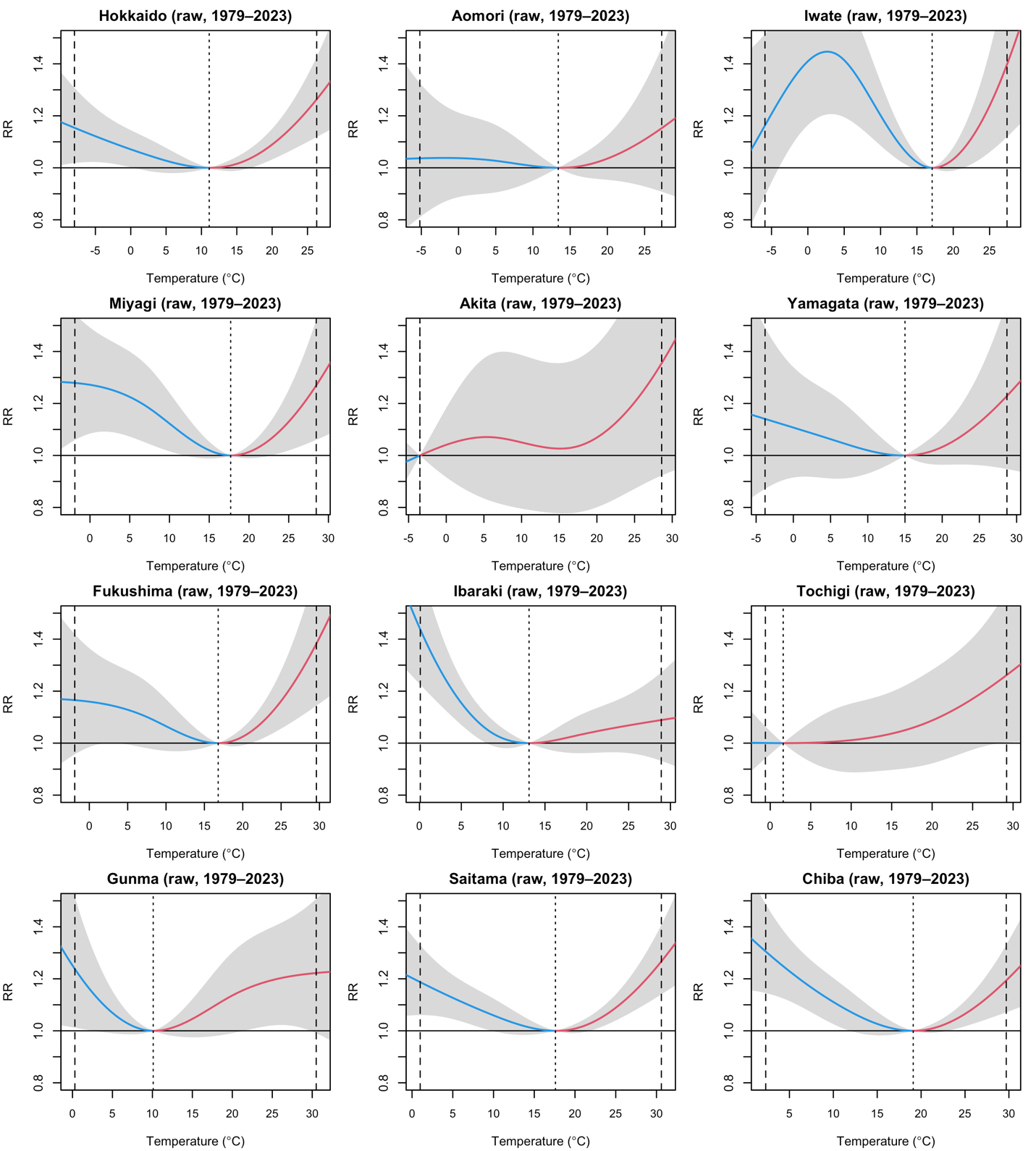


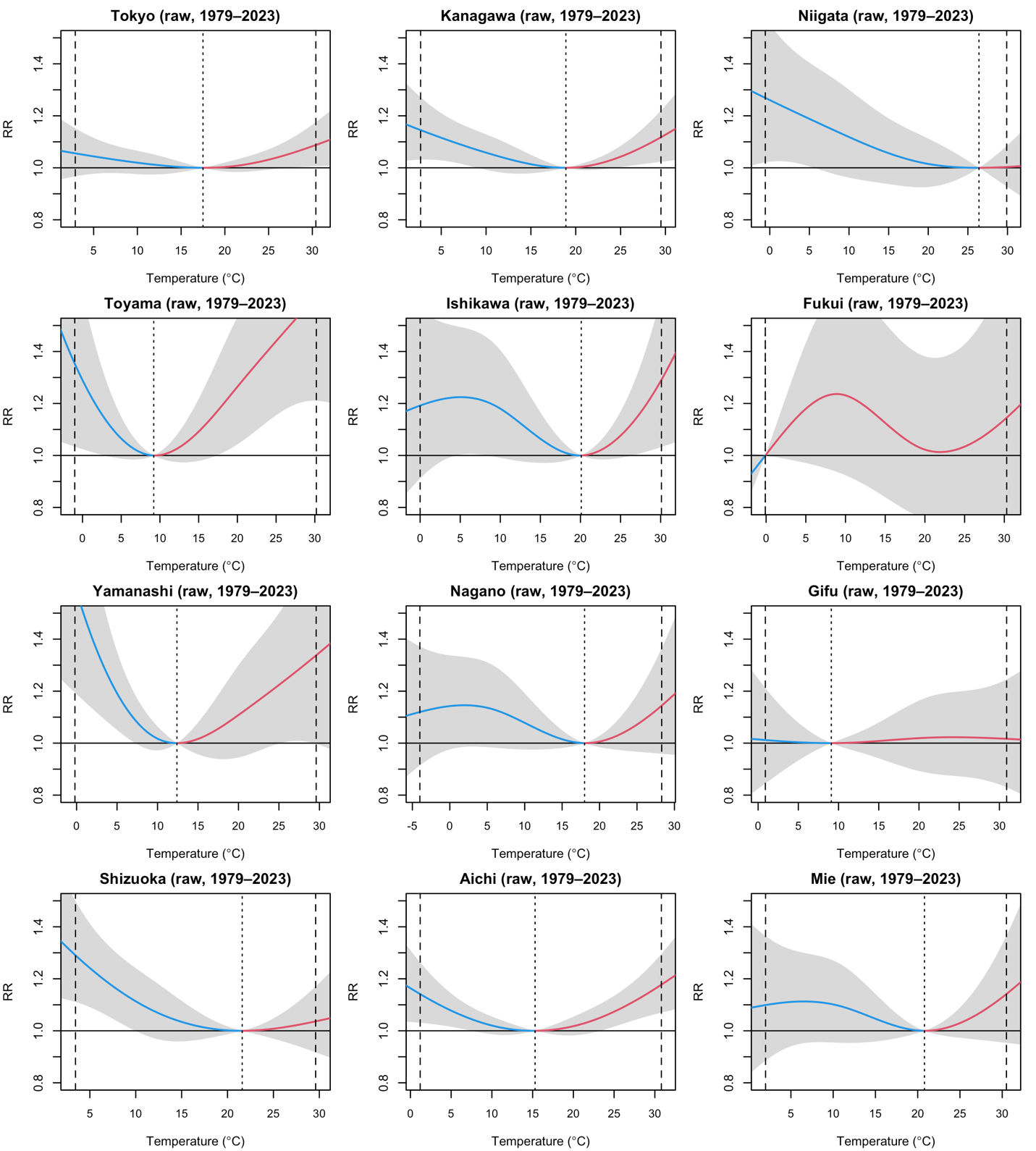


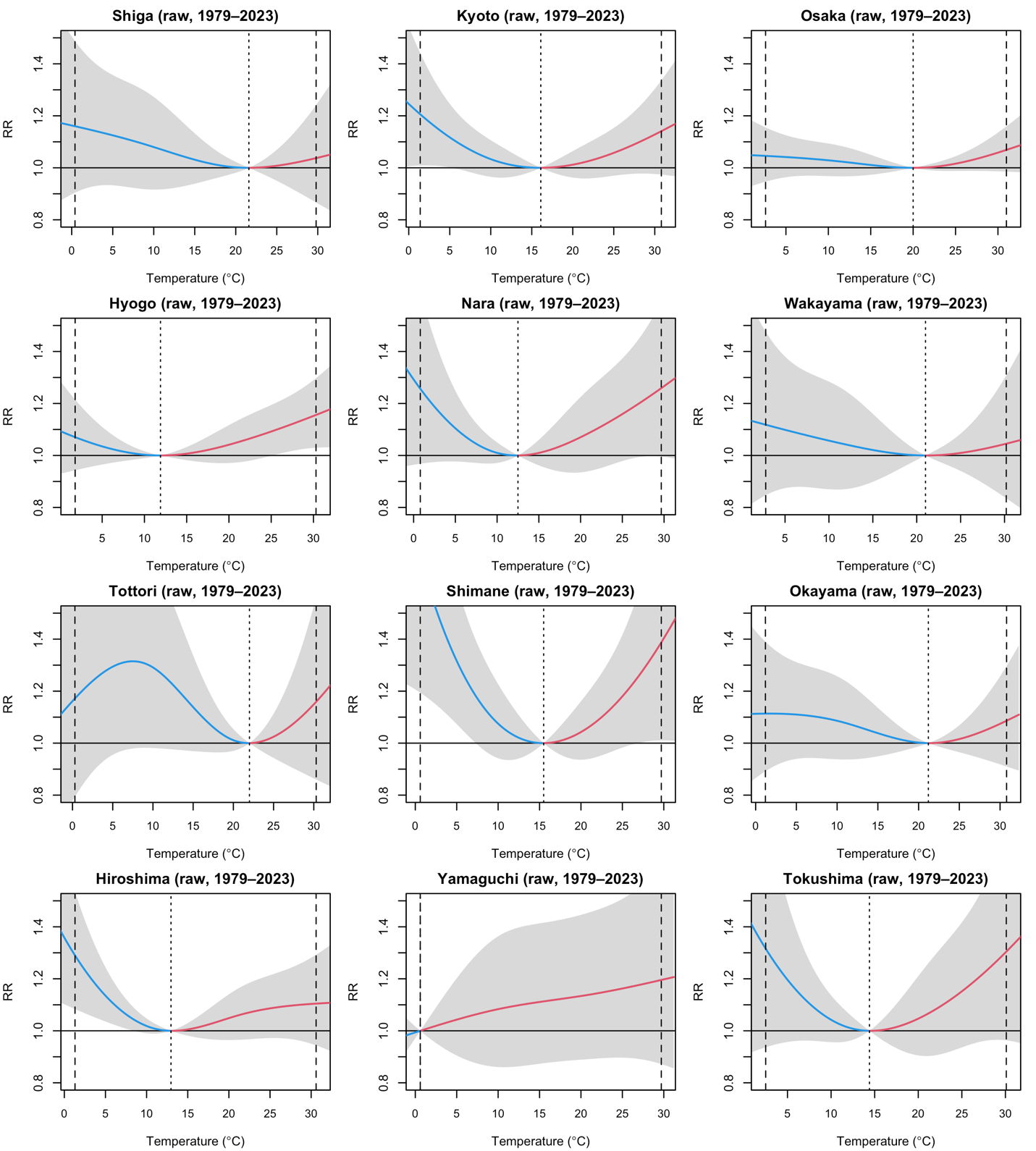


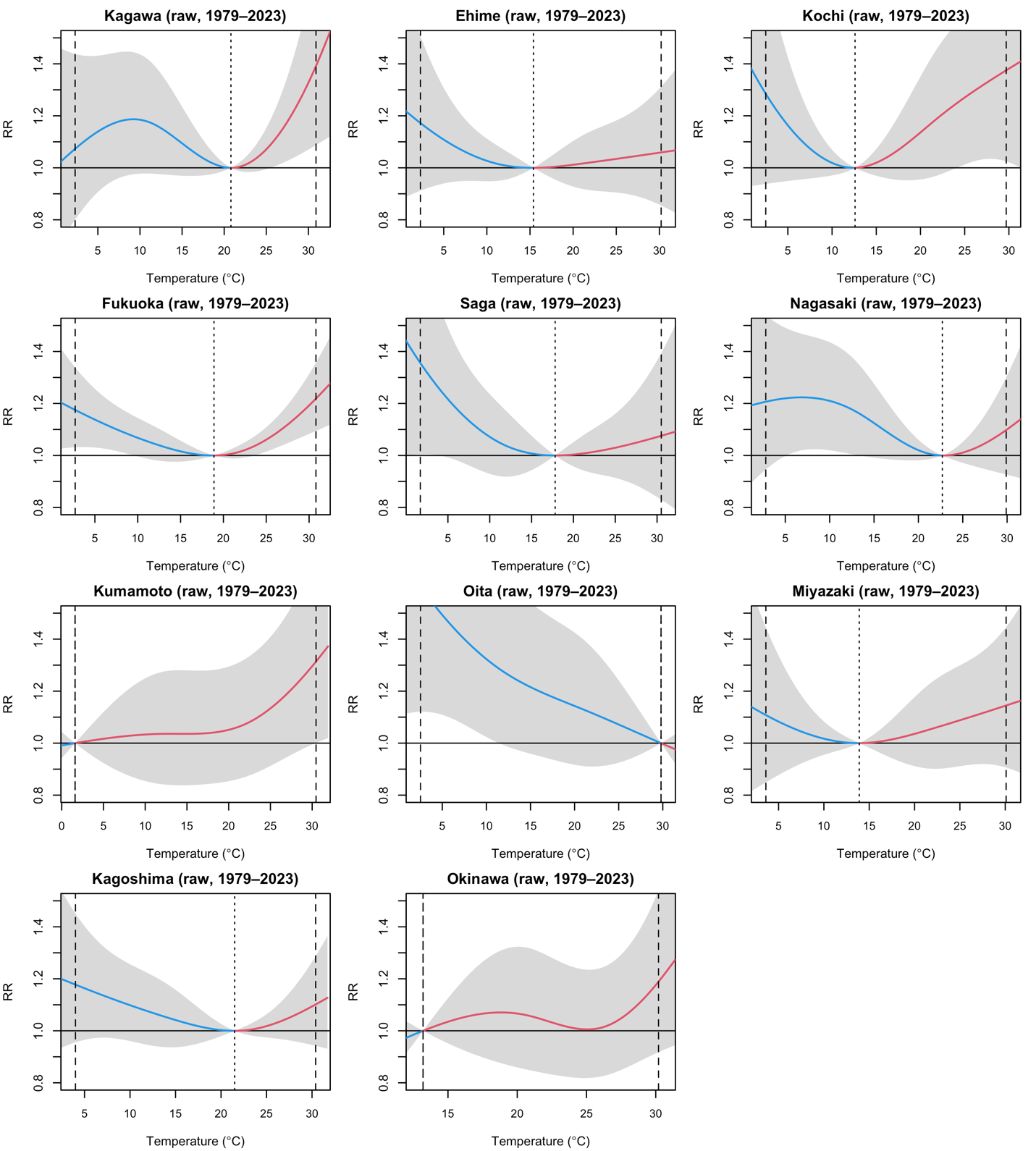


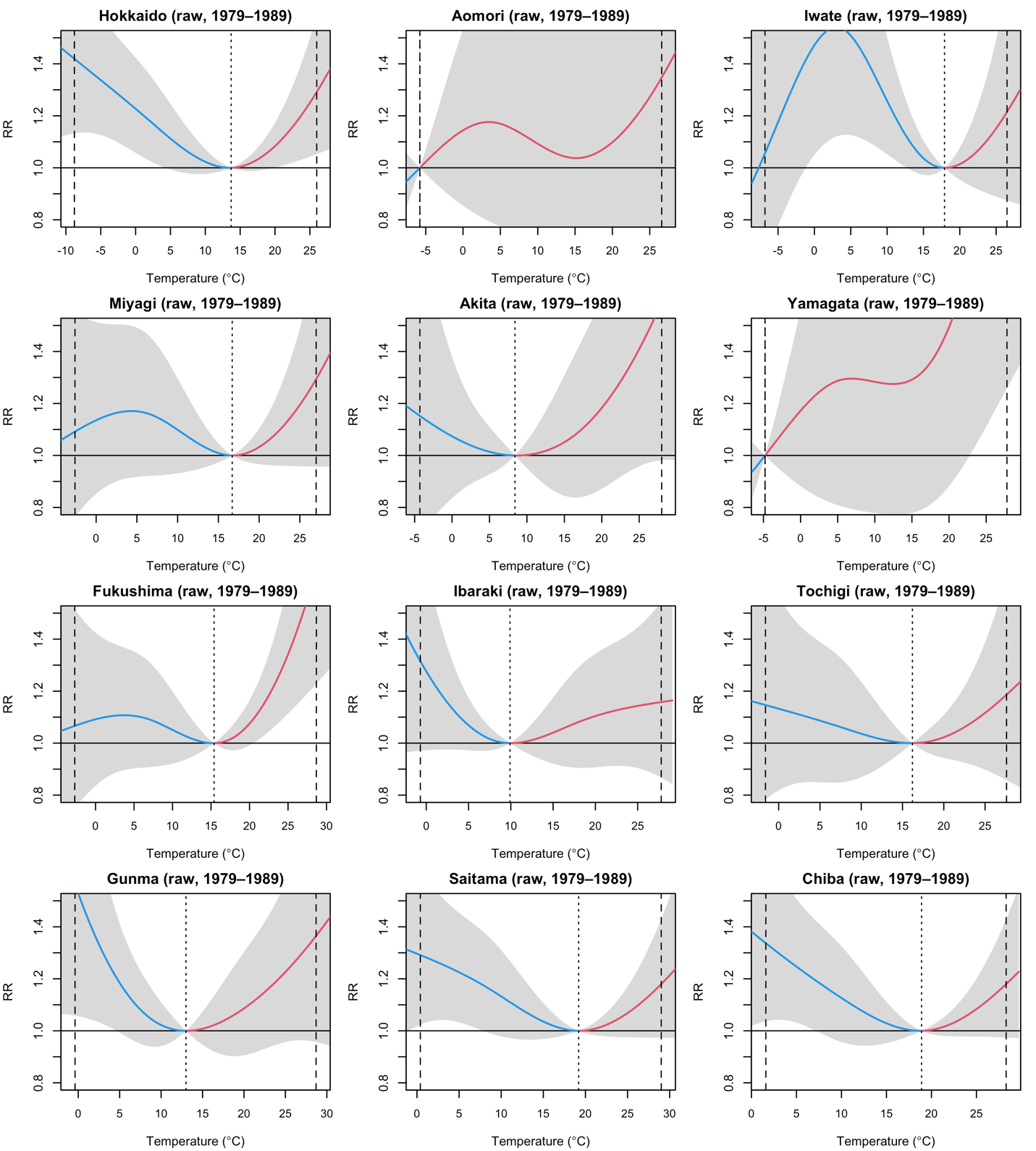


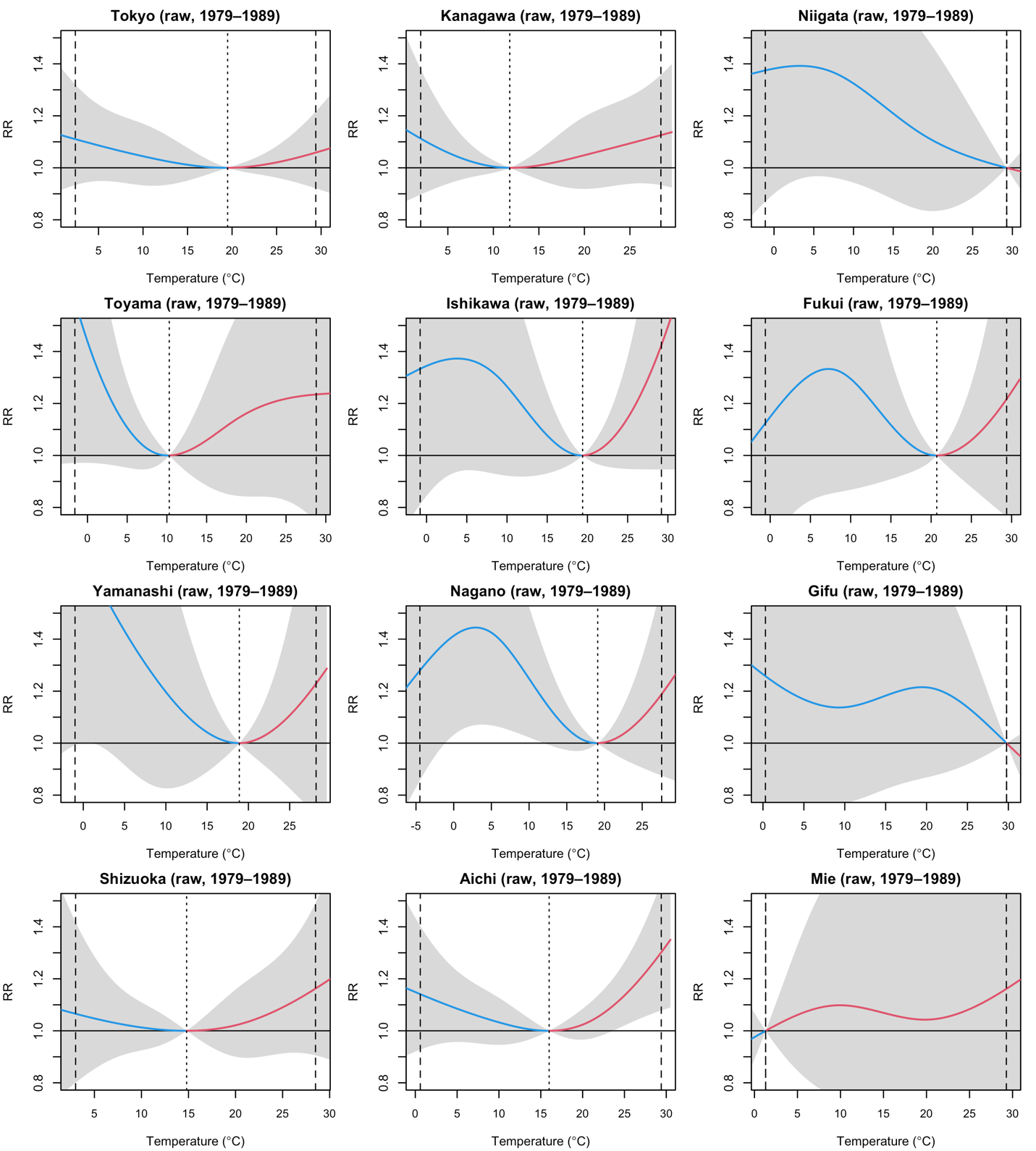


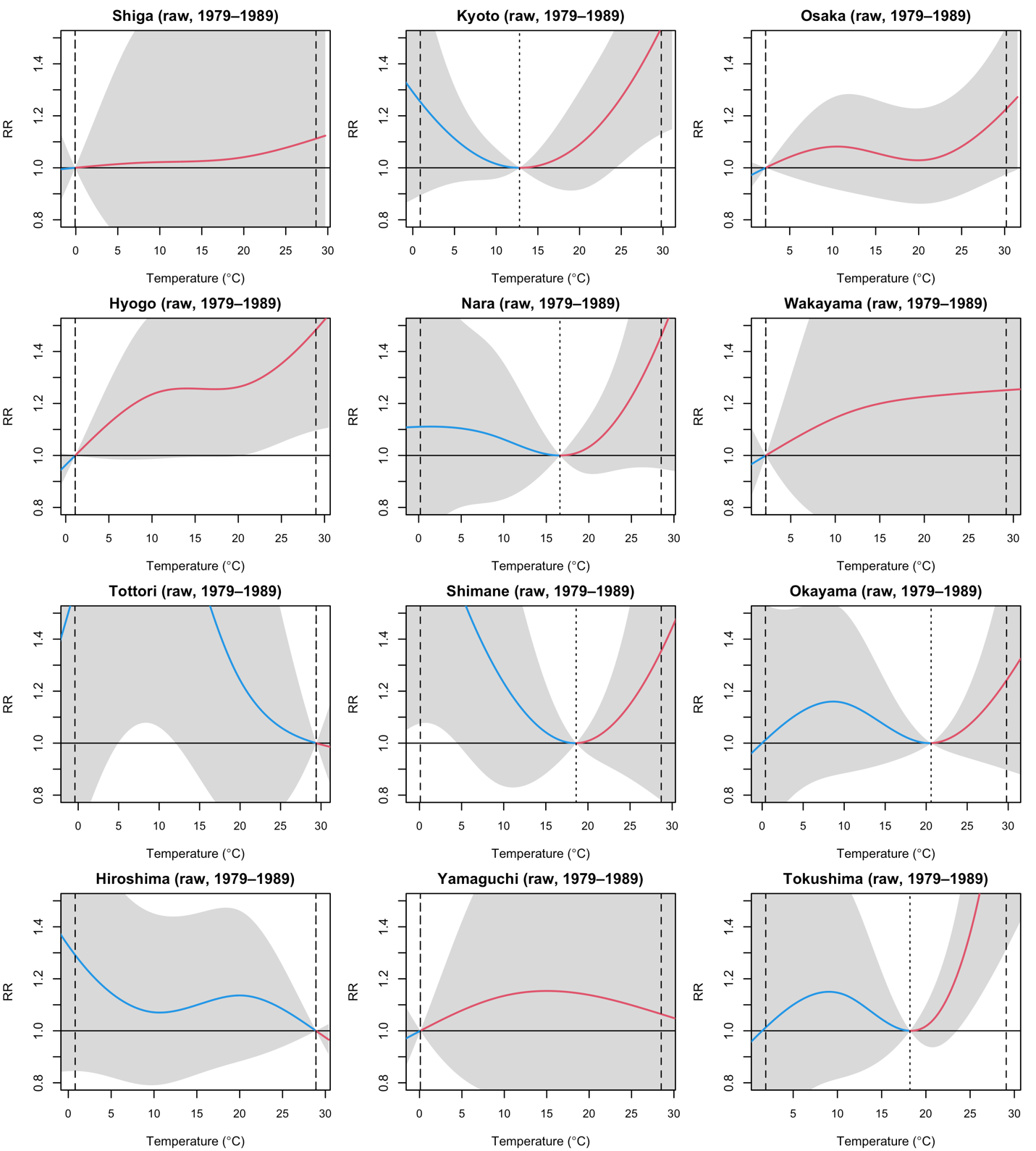


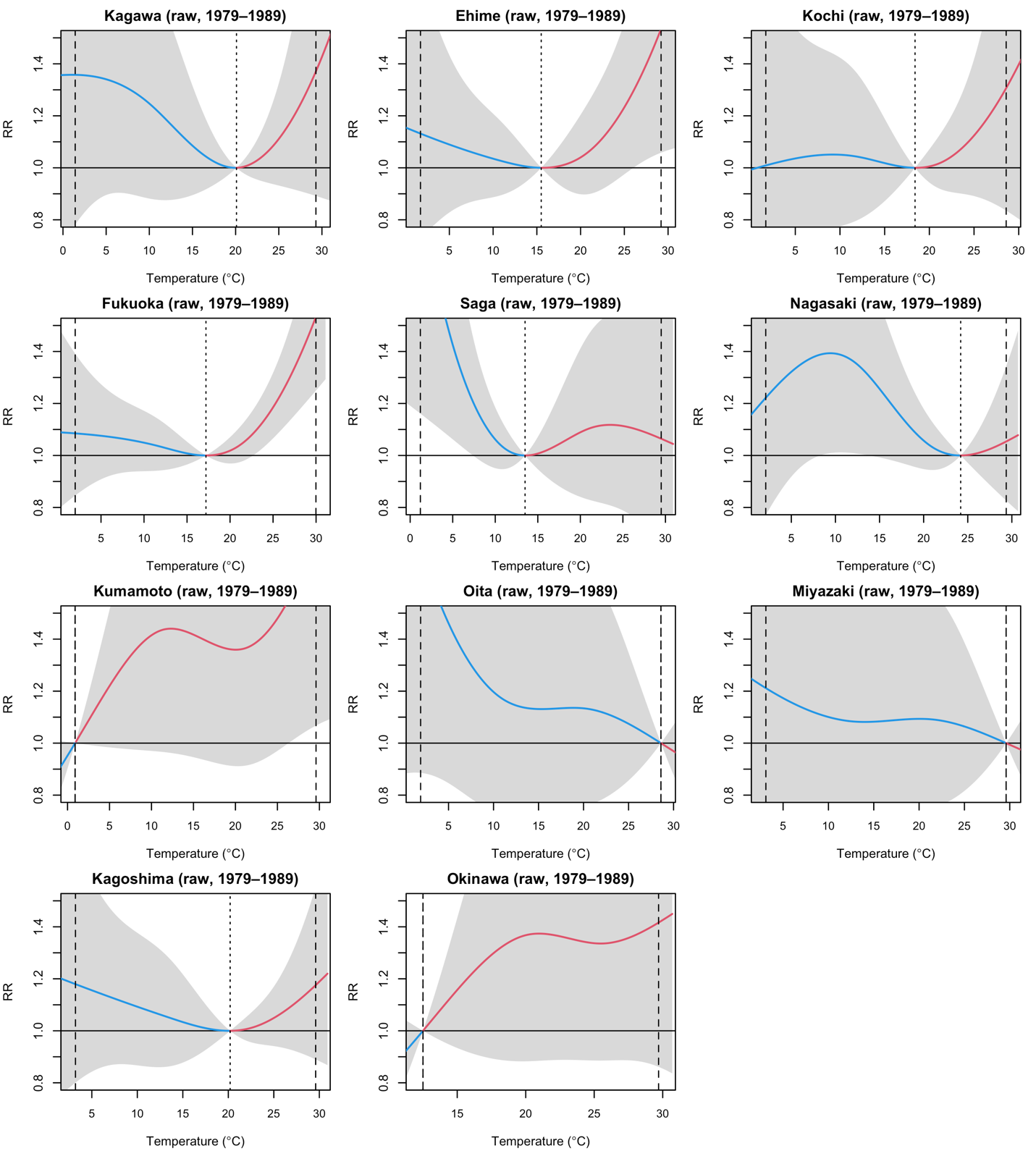


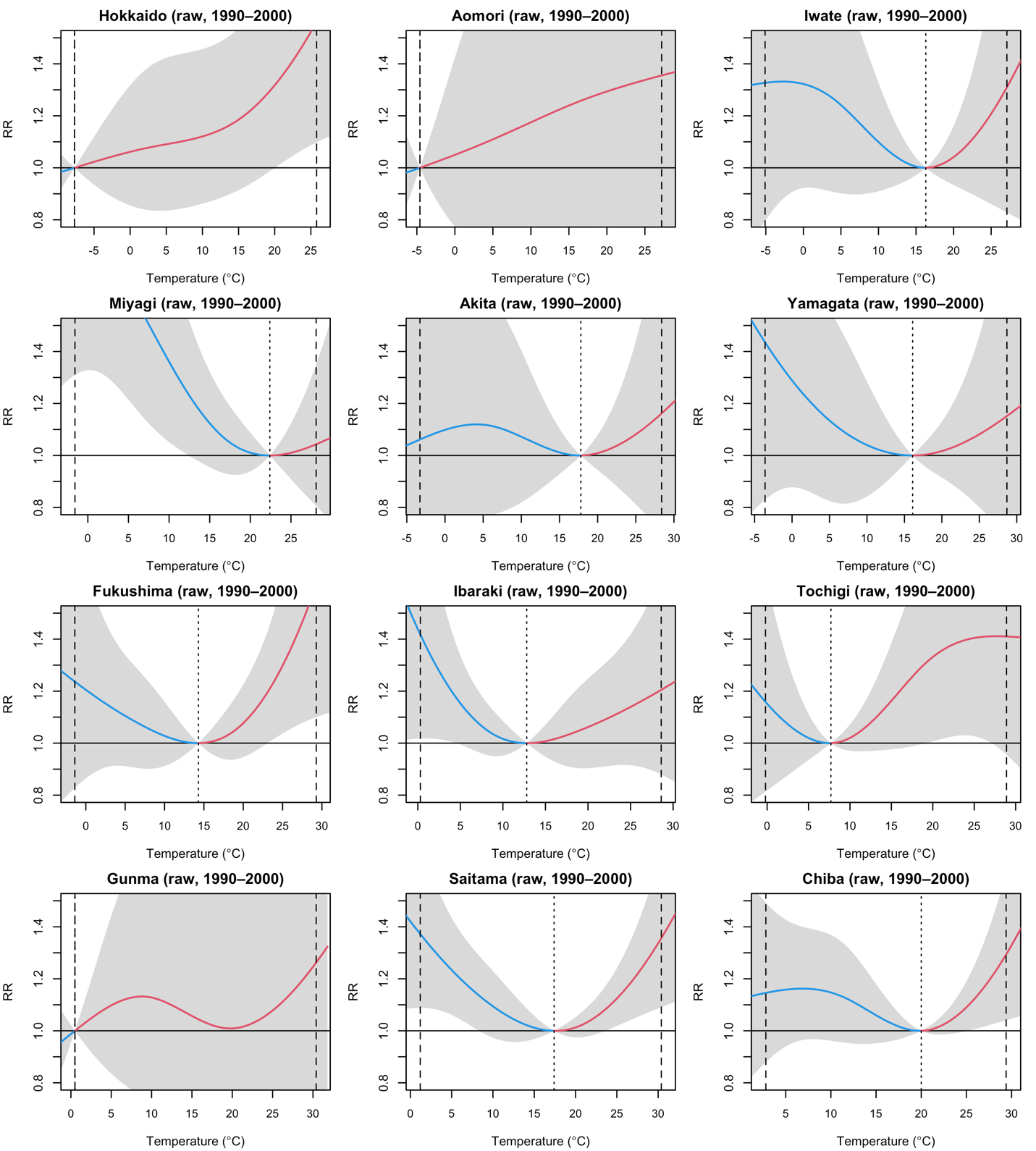


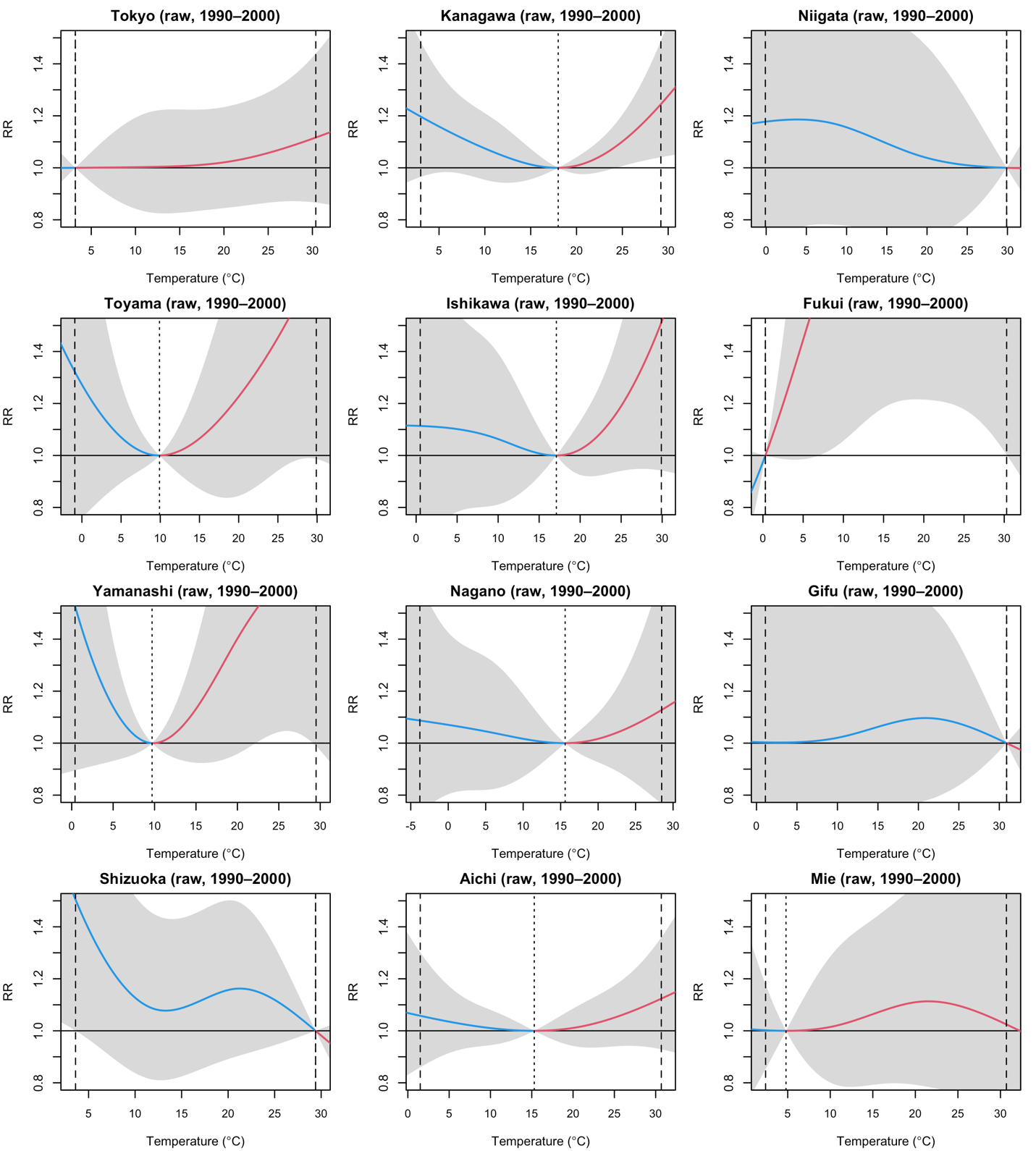


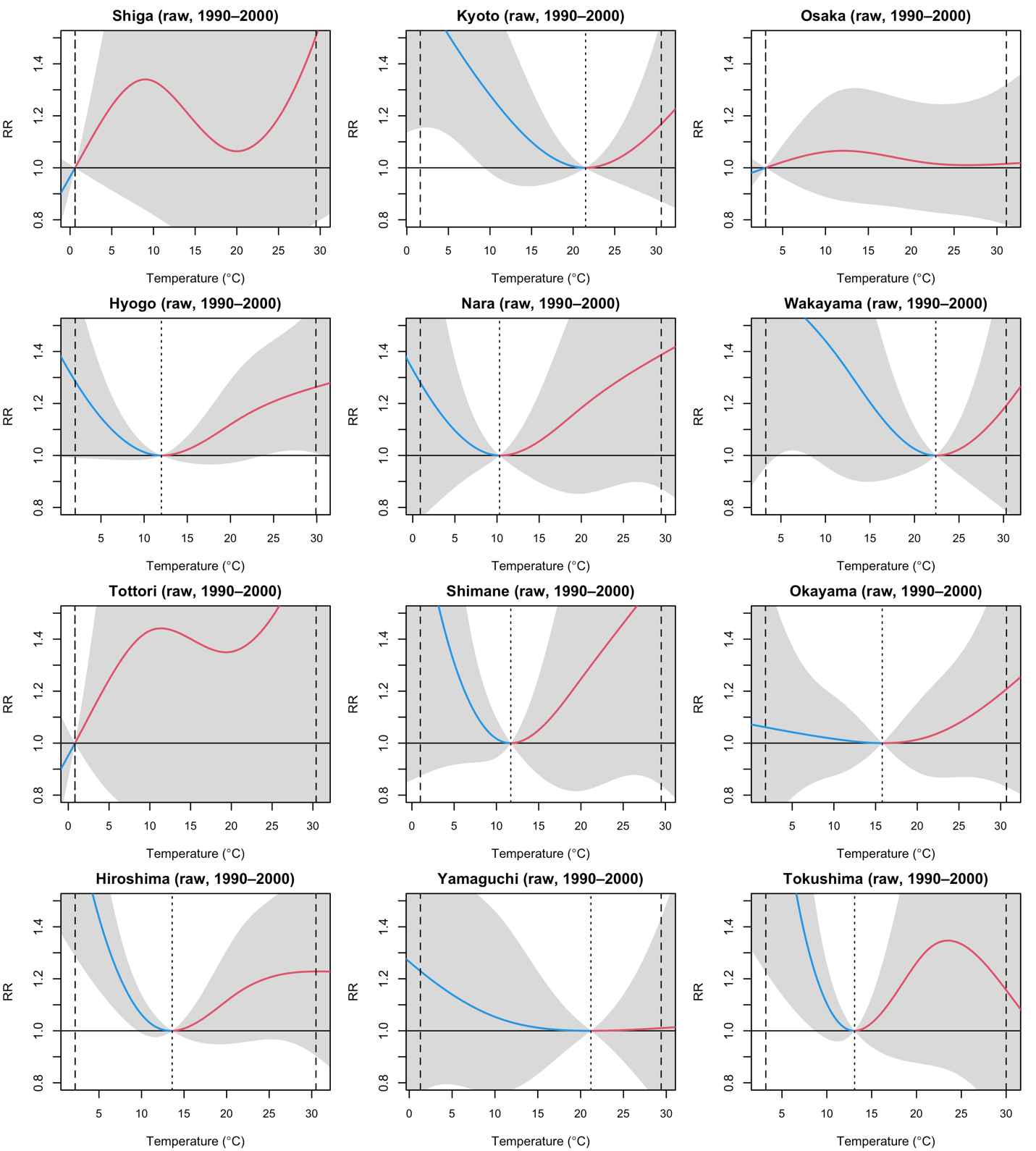


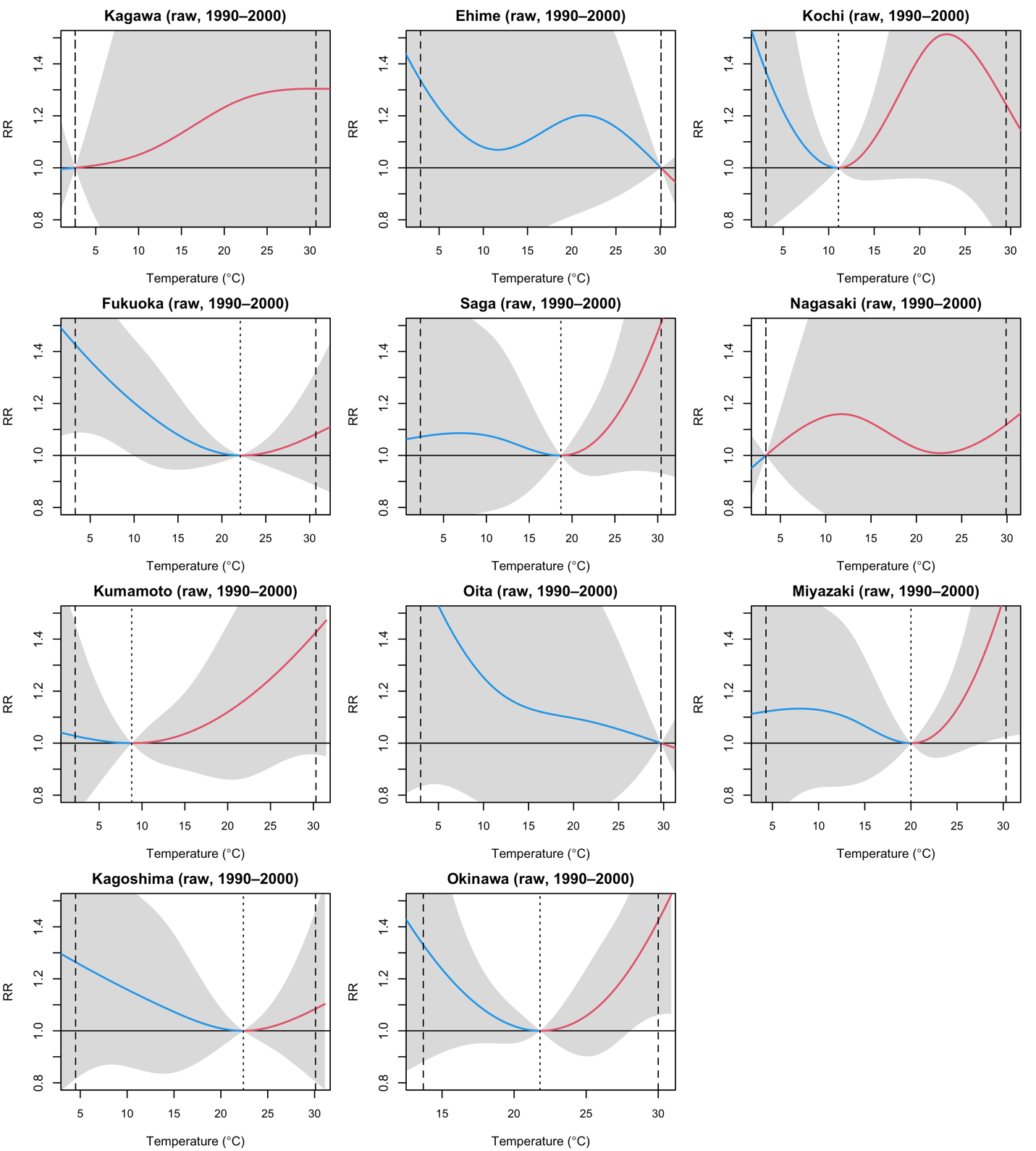


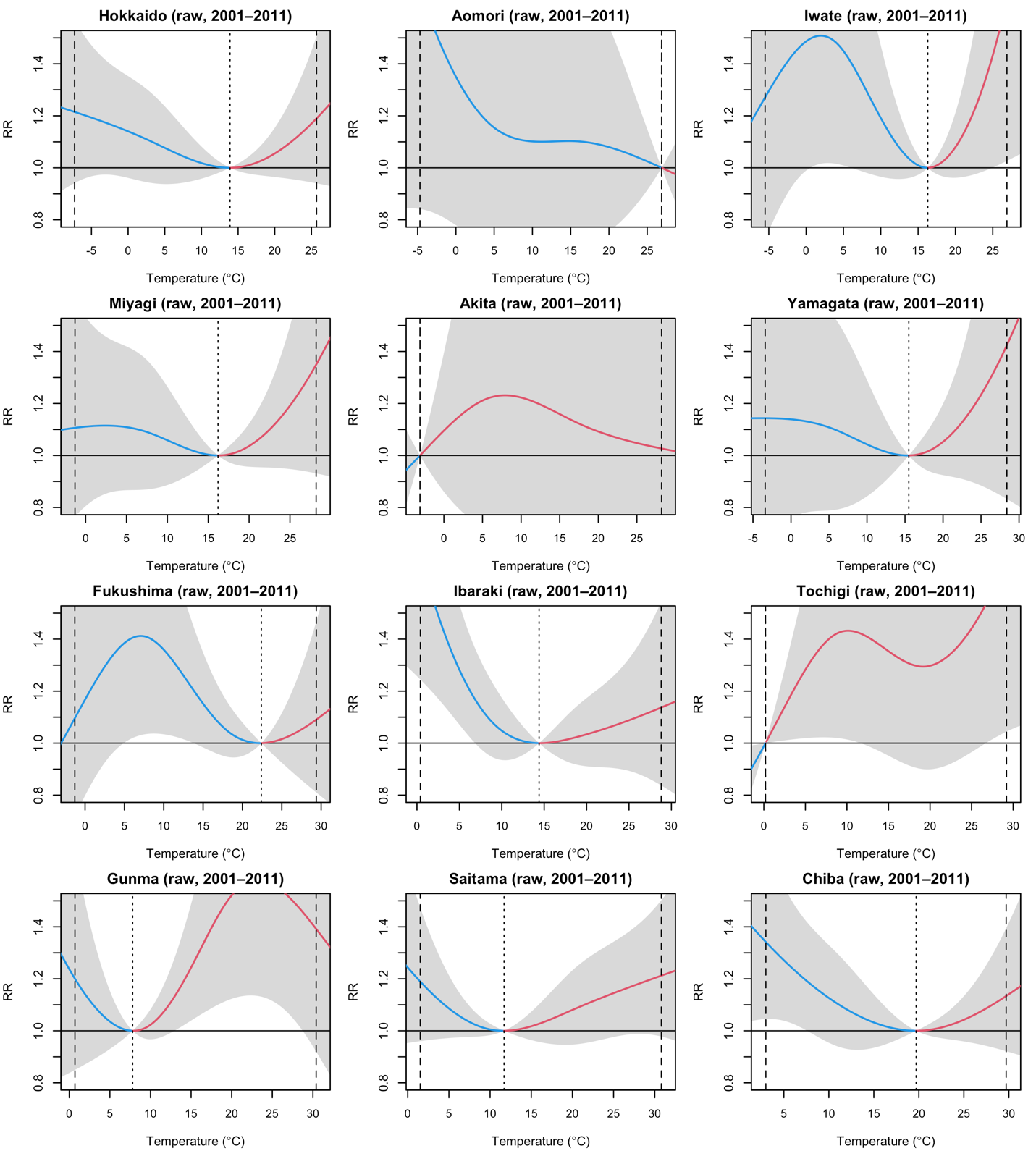


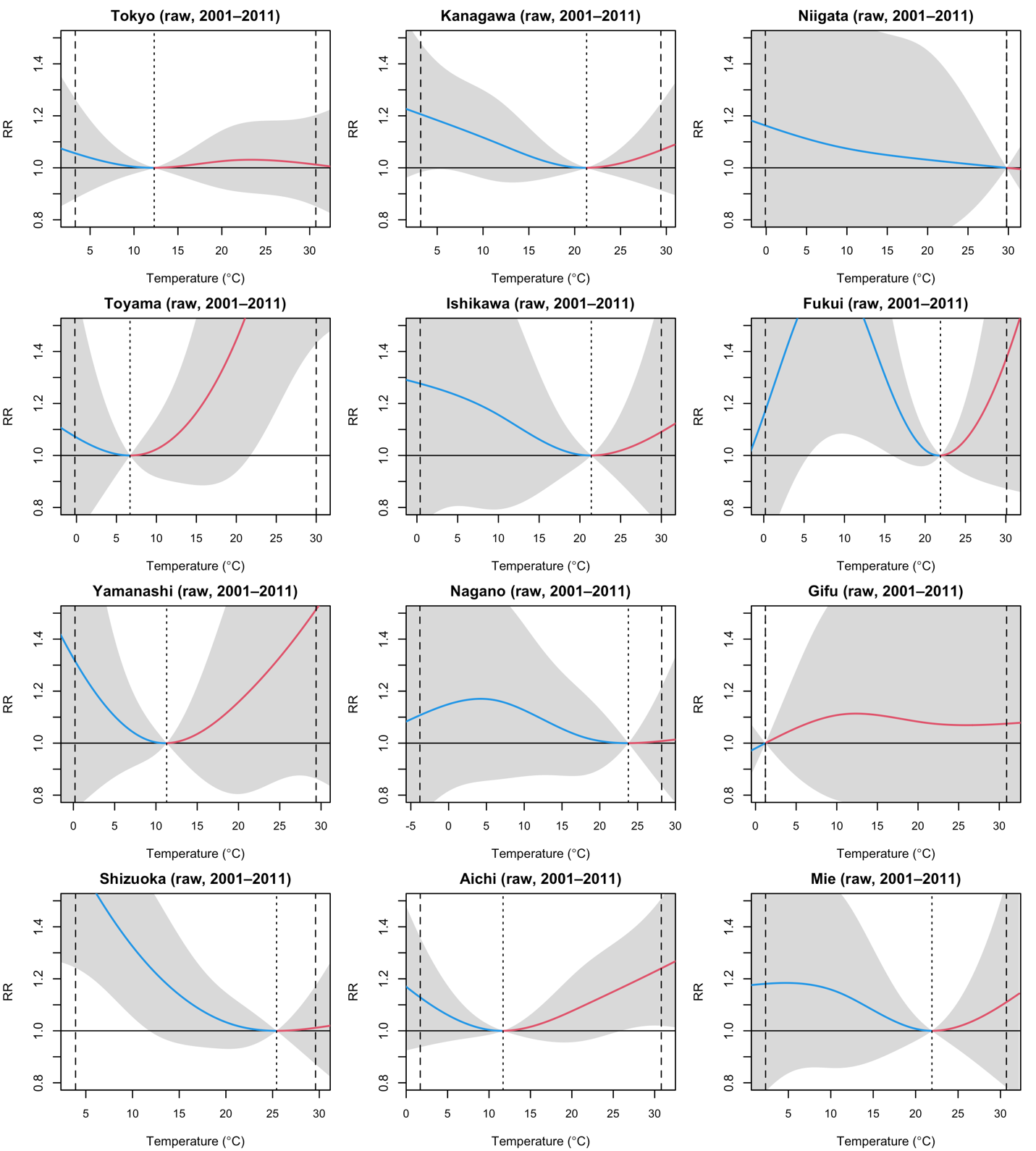


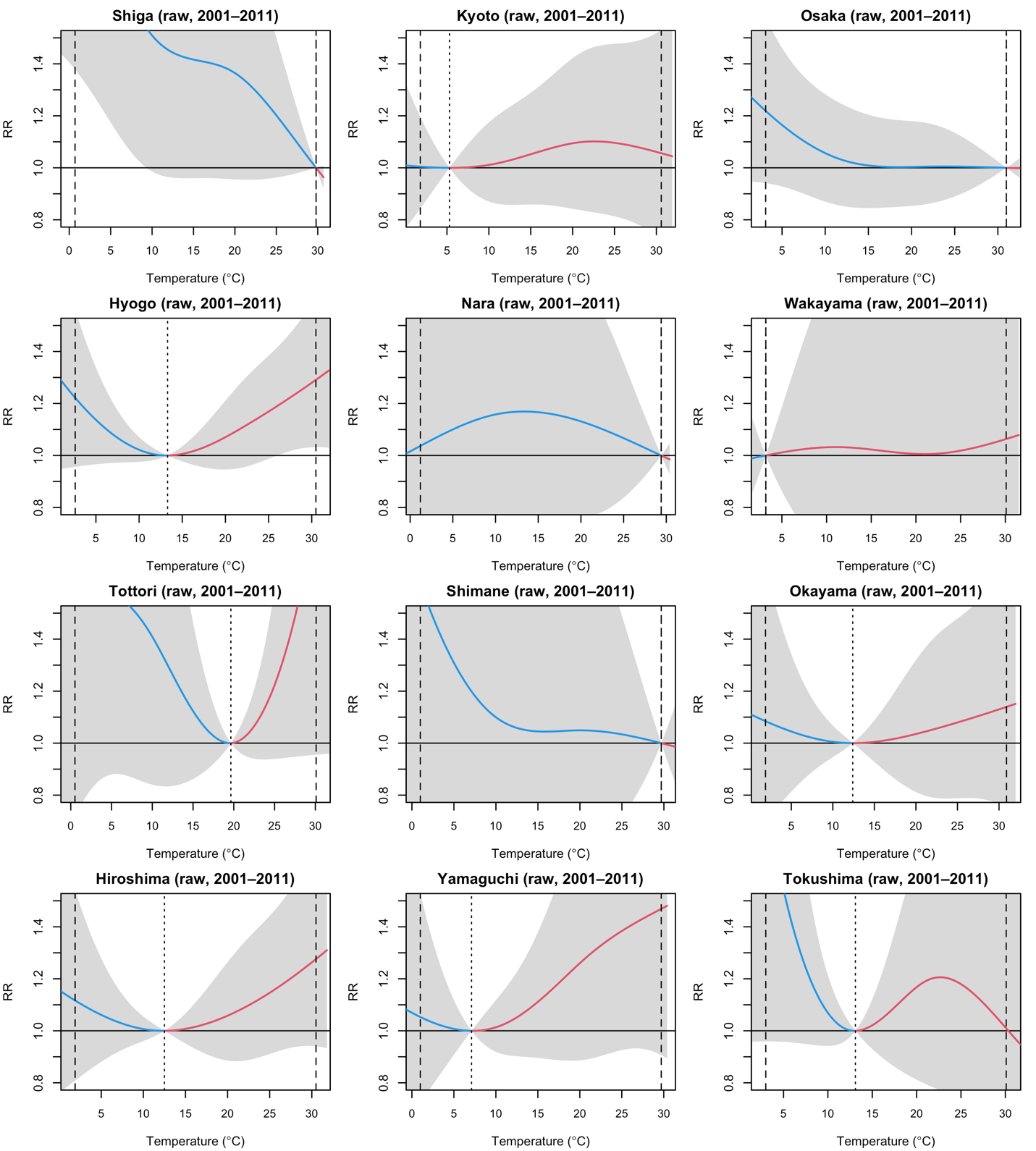


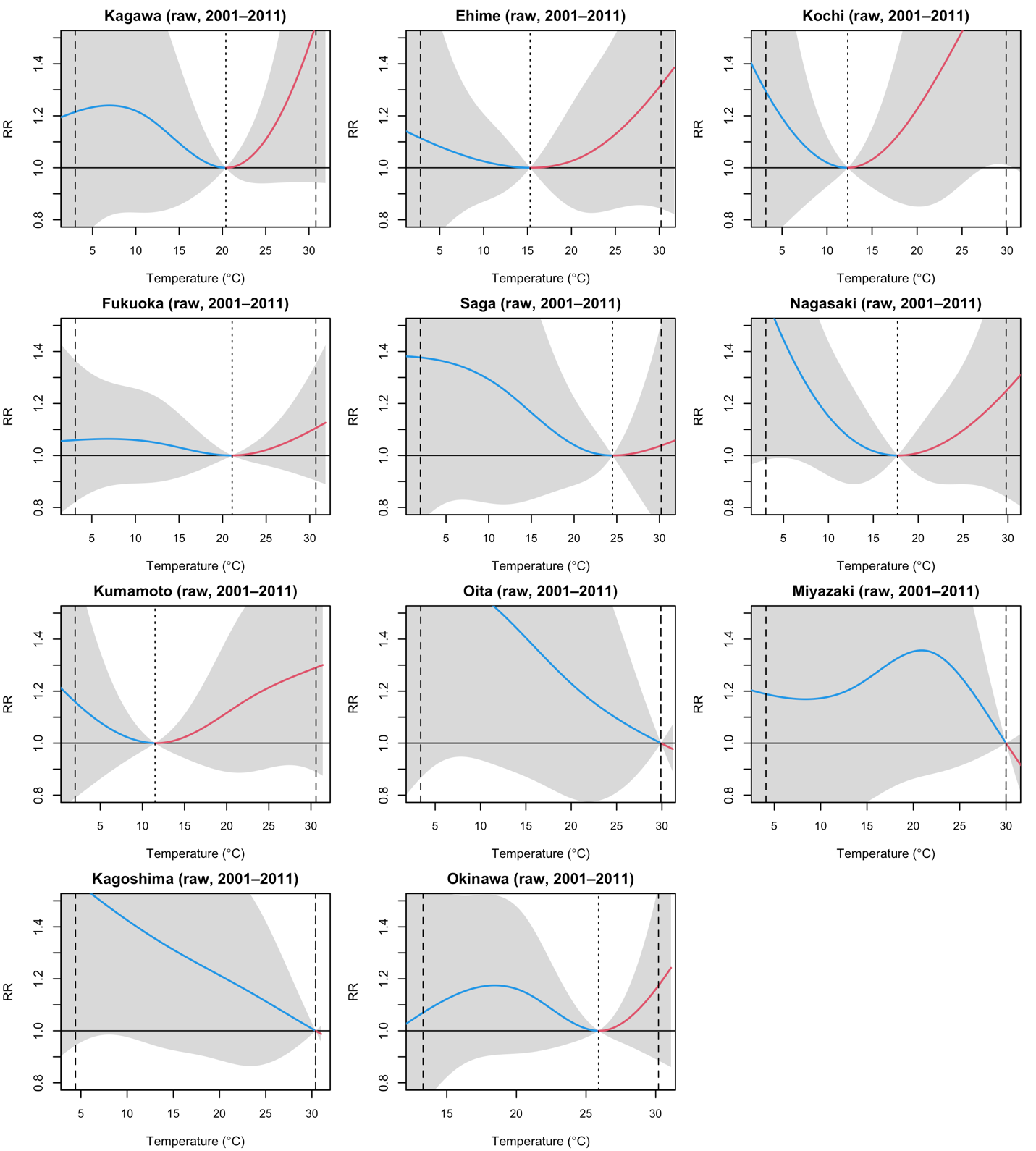


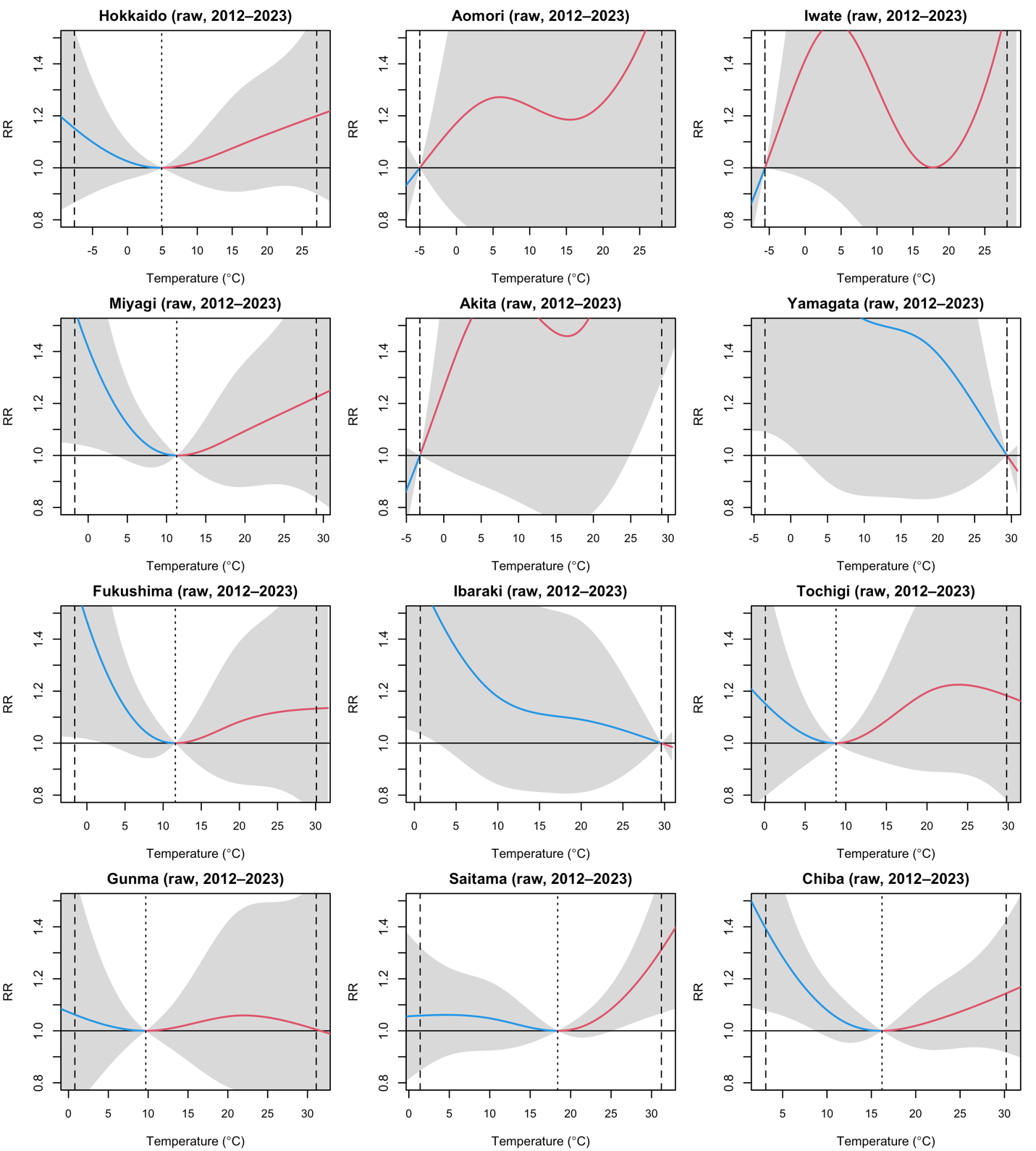


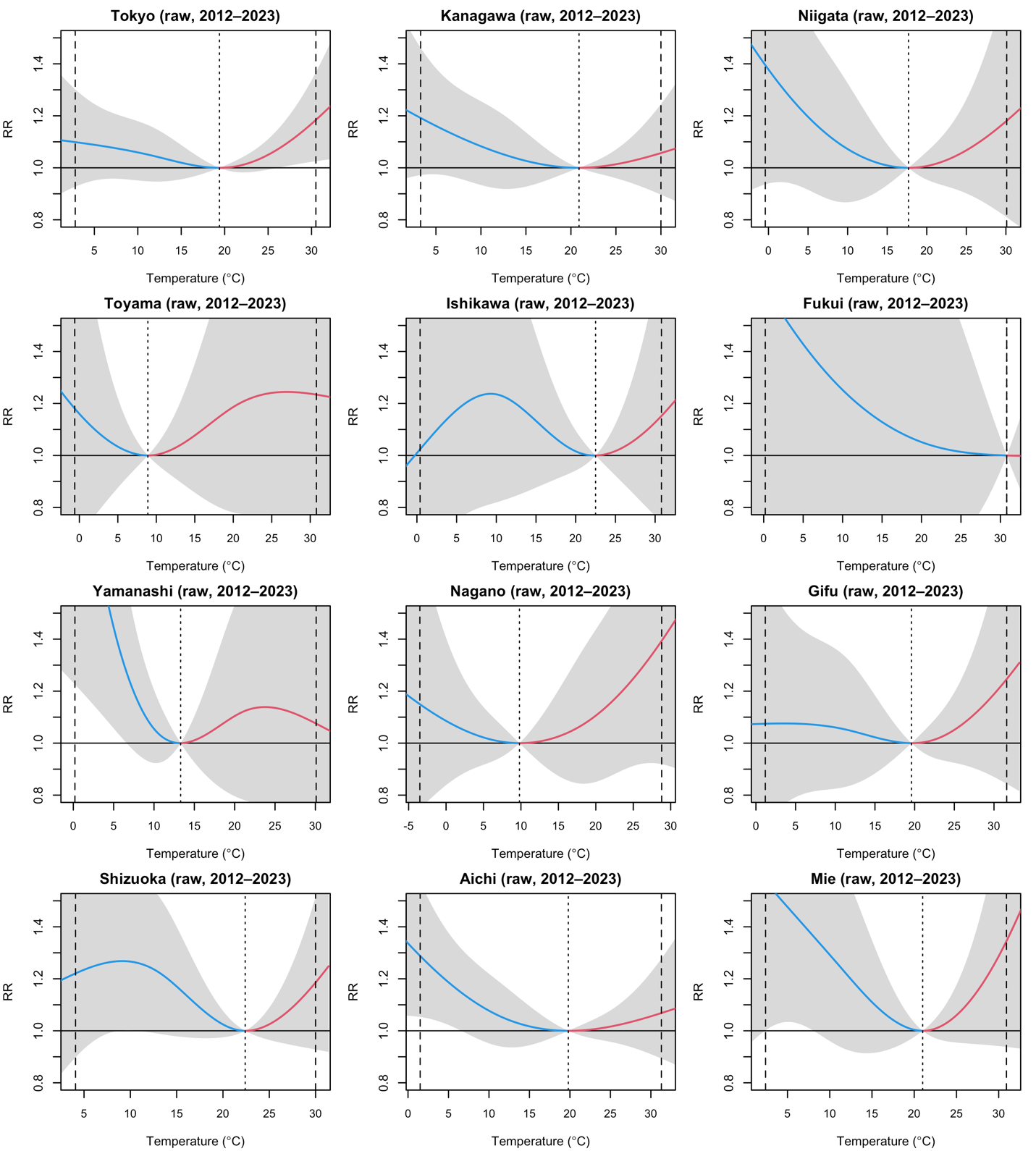


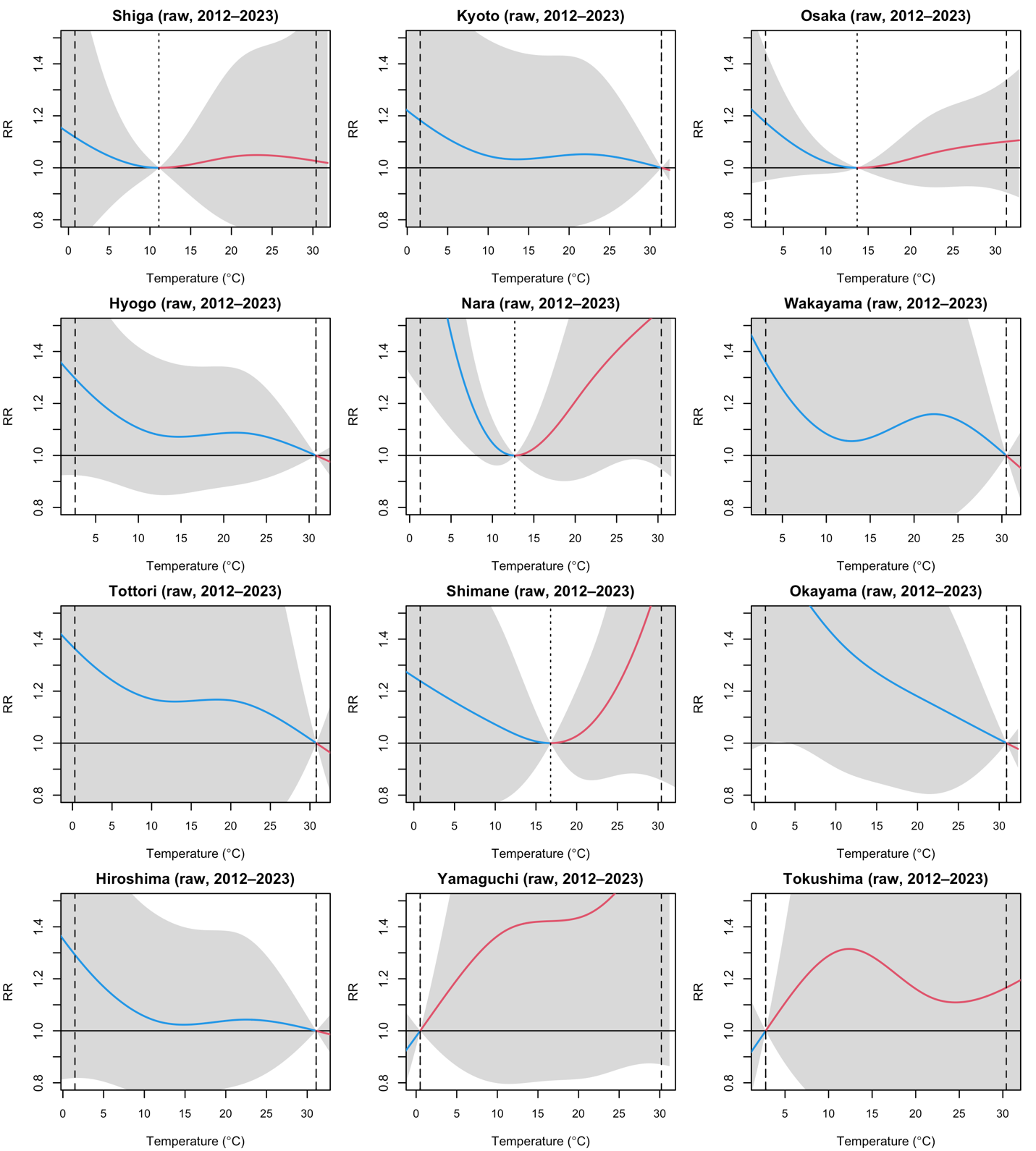


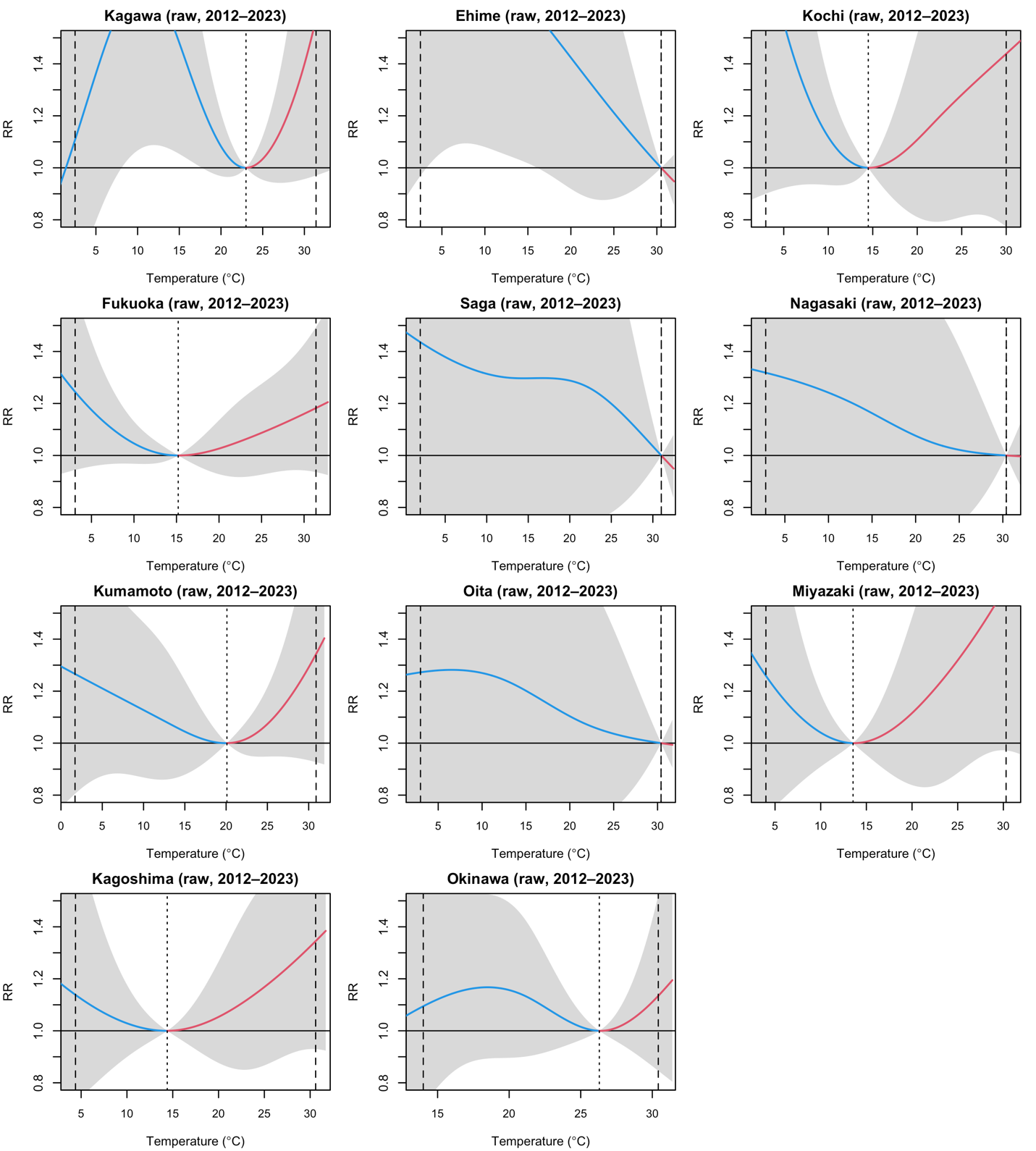


**Figure S7:** Prefecture specific lag-cumulative relative risk (RR) curves for mean temperature and preterm birth stratified by time periods (1979–2023 [entire study period], 1979–1989, 1990–2000, 2001–2011, 2012–2023) without best linear unbiased predictions (BLUPs).
